# The Landscape of Somatic Genetic Alterations in Breast Cancers from *CHEK2* Germline Mutation Carriers

**DOI:** 10.1093/jncics/pkz027

**Published:** 2019-04-27

**Authors:** Diana Mandelker, Rahul Kumar, Xin Pei, Pier Selenica, Jeremy Setton, Sasi Arunachalam, Ozge Ceyhan-Birsoy, David N Brown, Larry Norton, Mark E Robson, Hannah Y Wen, Simon Powell, Nadeem Riaz, Britta Weigelt, Jorge S Reis-Filho

**Affiliations:** 1 Department of Pathology, Memorial Sloan Kettering Cancer Center, New York, NY; 2 Department of Radiation Oncology, Memorial Sloan Kettering Cancer Center, New York, NY; 3 Department of Medicine, Memorial Sloan Kettering Cancer Center, New York, NY

## Abstract

Pathogenic germline variants in checkpoint kinase 2 (*CHEK2*), which plays pivotal roles in DNA damage response and cell cycle regulation, confer an increased breast cancer (BC) risk. Here, we investigated the phenotypic and genomic characteristics of 33 BCs from *CHEK2* germline mutation carriers (16 high-risk variants and 17 low-risk p.Ile157Thr variants). *CHEK2*-associated BCs from patients with high-risk germline variants were largely hormone receptor-positive (87%, 13/15), and 81% (13/16) exhibited loss of heterozygosity (LOH) of the *CHEK2* wild-type allele. Conversely, *CHEK2*-associated BCs from patients with the low-risk p.Ile157Thr variant displayed less-frequent loss of heterozygosity (5/17, 29%) and higher levels of CHEK2 protein expression than those with high-risk germline variants. *CHEK2*-associated BCs lacked a dominant mutational signature 3, a genomics feature of homologous recombination DNA repair deficiency (HRD). Our findings indicate that *CHEK2*-associated BCs are generally hormone receptor-positive and lack HRD-related mutational signatures, recapitulating the features of *ATM*-associated BCs. Specific *CHEK2* germline variants may have a distinct impact on tumor biology.

Checkpoint kinase 2 (CHEK2) is a serine-threonine kinase that is activated by double-strand DNA breaks and regulates cell cycle progression (1). *CHEK2* germline mutations are associated with an increased risk of breast cancer (BC) with an odds ratio of approximately 1.5–3.0 and an absolute risk of up to 37% for developing a BC by the age of 70 years (1–7). Founder mutations in *CHEK2* have been identified in multiple populations, and meta-analyses have shown that *CHEK2* truncating variants confer a higher BC risk than some missense variants, including *CHEK2* p.Ile157Thr (1–4,8). We sought to define the phenotypic characteristics and somatic genetic alterations of BCs with germline *CHEK2* variants by pooling whole-exome sequencing (WES) data from The Cancer Genome Atlas (TCGA) (9) and targeted sequencing data from Memorial Sloan Kettering-Integrated Mutation Profiling of Actionable Cancer Targets (MSK-IMPACT; Supplementary Figure 1 and Table 1, available online).

We identified 16 patients harboring a high-risk (ie, frameshift, nonsense, splice site, and high-risk missense) germline variant in *CHEK2* (n = 5 from TCGA and n = 11 from MSK-IMPACT; Figure 1). The methods are detailed in the Supplementary Methods (available online). The median age of BC diagnosis was 48.5 (range = 36–64) years (Supplementary Table 1, available online). Histologically, 11, 4, and 1 high-risk–variant *CHEK2*-associated BCs were invasive ductal, lobular (ILC), and mixed invasive carcinomas, respectively (Figure 1A, Supplementary Table 1, available online). Consistent with previous reports (5,6), all but two (13/15) high-risk-variant *CHEK2*-associated BCs with available hormone receptor data were estrogen receptor (ER)-positive, and three were human epidermal growth factor receptor 2 (*HER2*)-positive. High-risk–variant *CHEK2*-associated BCs harbored alterations affecting genes recurrently altered in ER-positive BCs (9,10), such as *PIK3CA* (44%), *GATA3* (25%), and *CDH1* mutations (20%, all but one ILCs), and *HER2* amplification (20%; Figure 1A, Supplementary Figure 2 and Table 2, available online). *TP53* mutations were found in only 12.5% (2/16) high-risk–variant *CHEK2*-associated BCs, consistent with the *TP53* mutation frequency in ER-positive BCs (9).

**Figure 1.**
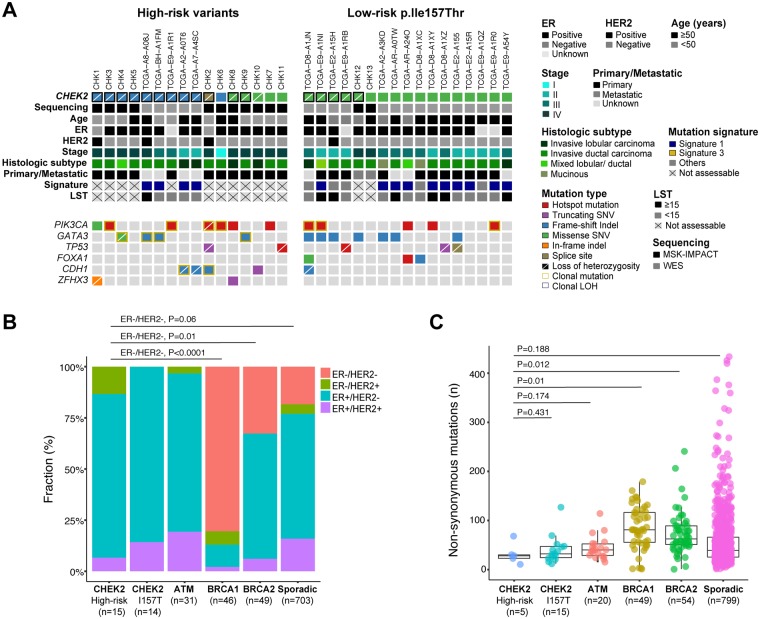
Genomic characterization of checkpoint kinase 2 (*CHEK2*)-associated breast cancers. **A**) Recurrent (present in ≥2 samples) nonsynonymous somatic mutations identified in 16 breast cancers (BCs) from patients with high-risk-variant *CHEK2* germline mutations using targeted massively parallel sequencing (MSK-IMPACT; n = 11) or whole-exome sequencing (WES; n = 5) and 17 from patients with the p.Ile157Thr *CHEK2* germline variant subjected to WES (n = 15) or MSK-IMPACT (n = 2). Phenobar provides information on *CHEK2* germline mutations, clinicopathologic features, dominant mutational signatures, and large-scale state transition (LST) scores. Clonal loss of heterozygosity (LOH) of the *CHEK2* locus and clonal mutations are displayed by black and yellow boxes, respectively. **B**) Estrogen receptor (ER) and human epidermal growth factor receptor 2 (*HER2*) status of High-risk–variant *CHEK2*-associated BCs (this study and TCGA) and *CHEK2*-associated p.Ile157Thr variant (this study and TCGA), and ATM-associated (Weigelt et al. [11] and TCGA) *BRCA1*-associated (TCGA and ICGC), *BRCA2*-associated (TCGA and ICGC), and sporadic (ie, non-*BRCA1/BRCA2/ATM/CHEK2*) BCs (TCGA) (Fisher exact test). **C**) Comparison of the number of nonsynonymous somatic mutations in High-risk–variant *CHEK2*-associated (TCGA), *CHEK2*-associated p.Ile157Thr variant (TCGA), *ATM*-associated (Weigelt et al. [11] and TCGA), *BRCA1*-associated (TCGA and ICGC), *BRCA2*-associated (TCGA and ICGC), and sporadic cancers (ie, non-*BRCA1/BRCA2/ATM/CHEK2*) BCs (TCGA) subjected to WES. *CHEK2*-associated BCs displayed a statistically significantly lower number of nonsynonymous somatic mutations than *BRCA1*- and *BRCA2*-associated BCs (Mann-Whitney U test). Box plot markings from bottom to top: minimum value, first quartile, median, third quartile, maximum value. Indel = small insertion and deletion; MSK-IMPACT = Memorial Sloan Kettering-Integrated Mutation Profiling of Actionable Cancer Targets; SNV = single-nucleotide variant; TCGA = The Cancer Genome Atlas.

Allele-specific copy number analysis revealed bi-allelic inactivation of *CHEK2,* through loss of heterozygosity (LOH) of the wild-type allele, in 81% (13/16) high-risk-variant *CHEK2*-associated BCs, a frequency similar to that reported for *BRCA1* (94%), *BRCA2* (71%), and *ATM* (79%) (11–14). When we compared the high-risk–variant *CHEK2*-associated BCs in this study with those of *BRCA1*- and *BRCA2*-associated BCs from TCGA (n = 41) (9) and the International Cancer Genome Consortium (ICGC; n = 62) (15) (Supplementary Table 3, available online), we found that high-risk–variant CHEK2-associated BCs were statistically significantly less frequently ER-negative/*HER2*-negative (*BRCA1*, *P* < .0001; *BRCA2*, *P* = .01, Fisher exact test, Figure 1B) and harbored a statistically significantly lower number of nonsynonymous mutations (ie, tumor mutation burden, *P* = .01 for *BRCA1* and *BRCA1*, Mann-Whitney U test, Figure 1C) than the 49 *BRCA1*-associated or 54 *BRCA2*-associated BCs analyzed. We also found a significantly lower frequency of *TP53* mutations than the 49 *BRCA1*-associated BCs included in this study (Figure 1, B and C; Supplementary Figures 3–5, available online). Conversely, *ATM*- and high-risk–variant *CHEK2*-associated BCs displayed similar ER and *HER2* status; mutation burden; frequency of *PIK3CA, GATA3*, and *TP53* mutations; and pattern of gene copy number alterations (Figure 1, B and C, Supplementary Figures 3–5, available online) (11).

We then investigated the clinicopathologic and genomic profiles of a set of 17 *CHEK2*-associated BCs from patients carrying the low-risk p.Ile157Thr germline variant, which has been associated with an approximate 1.5-fold increase in BC risk (Figure 1A;Supplementary Tables 1 and 2, Supplementary Figure 6, available online) (3,4). A comparison of these cases with high-risk–variant *CHEK2*-associated BCs revealed a numerically but not statistically significantly later BC onset in *CHEK2* p.Ile157Thr carriers than in *CHEK2* high-risk–variant carriers (58 vs 48.5 years, *P* = .07, Mann-Whitney U test; Supplementary Figure 5A, available online). No significant differences in ER-positivity (100% vs 87%, *P* = .48, Fisher exact test) or genes frequently mutated between low- and high-risk–variant *CHEK2*-associated BCs were detected (Figure 1B;Supplementary Figure 3, available online). Despite these similarities, but consistent with the low penetrance of the *CHEK2* p.Ile157Thr variant, a significant difference in the frequency of LOH of the *CHEK2* wild-type allele was observed between these cases (29%, 5/17) and those harboring a *CHEK2* high-risk germline variant (81%, 13/16, *P* = .005, Fisher exact test). Furthermore, LOH affecting the *CHEK2* locus was present in 41% (n = 302) of 736 TCGA BCs affecting patients who lacked a *CHEK2* germline variant, a frequency similar to that of *CHEK2* LOH in BC patients with the low-risk *CHEK2* p.Ile157Thr germline variant (*P* = .46, Mann-Whitney U test) but statistically significantly lower than that observed in high-risk–variant *CHEK2*-associated BCs (*P* = .002, Mann-Whitney U test; Figure 2A). Further, the CHEK2 protein levels were statistically significantly lower in high-risk–variant *CHEK2*-associated BCs than in those with the p.Ile157Thr germline variant (Figure 2B). Given that some *CHEK2* germline variants may confer a higherrisk of BC susceptibility than the *CHEK2* p.Ile157Thr allele, our findings are consistent with the hypotheses that either, in a subset of BCs, the *CHEK2* p.Ile157Thr variant confers BC susceptibility through a biological mechanism distinct from that of high-risk *CHEK2* mutations or this variant may constitute an incidental finding (ie, sporadic BCs developing in the context of a *CHEK2* p.Ile157Thr germline variant).

**Figure 2.**
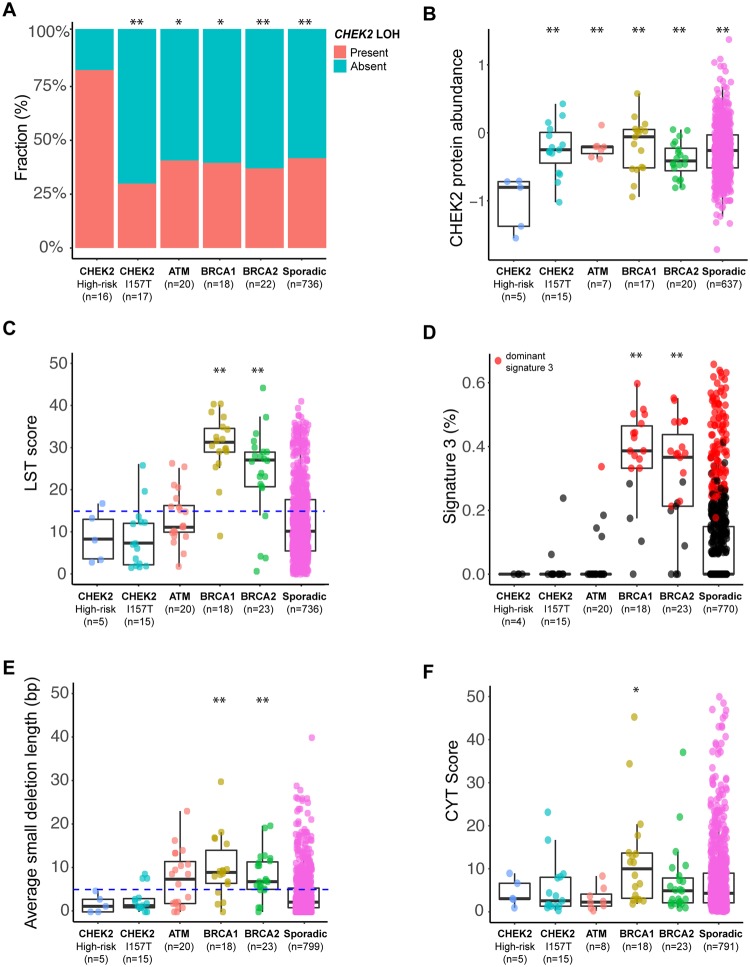
Checkpoint kinase 2 (*CHEK2*) loss of heterozygosity, CHEK2 protein expression, and homologous recombination DNA repair features in *CHEK2*-associated, *ATM*-associated, *BRCA1*-associated. and *BRCA2*-associated breast cancers. **A**) Distribution of loss of heterozygosity (LOH) of the *CHEK2* locus in breast cancers (BCs) from germline carriers of *CHEK2* high-risk variants (this study and TCGA), *CHEK2* Ile157Thr variants (TCGA), pathogenic germline variants of *ATM* (Weigelt et al. [11] plus TCGA), *BRCA1* (TCGA) or *BRCA2* (TCGA), and non-*CHEK2/ATM/BRCA1/BRCA2* (ie, sporadic; TCGA) BCs. **B**) Comparison of the CHEK2 reverse phase protein array (RPPA) data from TCGA between BCs in carriers of high-risk–variant *CHEK2*, *CHEK2* p.Ile157Thr, *ATM*, *BRCA1* and *BRCA2* germline variants, and non-*CHEK2/ATM/BRCA1/BRCA2* (ie, sporadic) BCs. **C**) Large-scale state transition (LST) scores in high-risk–variant *CHEK2*-associated (TCGA), *CHEK2*-associated p.Ile157Thr variant (TCGA), *ATM*-associated (Weigelt et al. [11] and TCGA), *BRCA1*-associated (TCGA), *BRCA2*-associated (TCGA), and sporadic (ie, non-*BRCA1/BRCA2/ATM/CHEK2*) BCs (TCGA) subjected to WES. The blue dashed line indicates the cutoff used to define high LST scores (≥15). **D**) Mutational signature 3 in high-risk-variant *CHEK2*-associated (TCGA), *CHEK2*-associated p.Ile157Thr variant (TCGA), ATM-associated (Weigelt et al. [11] and TCGA), BRCA1-associated (TCGA), BRCA2-associated (TCGA), and sporadic cancers (ie, non-BRCA1/BRCA2/ATM/CHEK2) BCs (TCGA) subjected to WES. The contribution of mutational signature 3 to the mutational repertoire of a given case is shown (percentage). BCs with a dominant signature 3 are depicted in red. **E**) Average small deletion length in base pairs (bp) in high-risk-variant *CHEK2*-associated (TCGA), *CHEK2*-associated p.Ile157Thr variant (TCGA), ATM-associated (Weigelt et al. [11] and TCGA), BRCA1-associated (TCGA), BRCA2-associated (TCGA), and sporadic (ie, non-*BRCA1/BRCA2/ATM/CHEK2*) BCs (TCGA) subjected to WES. The blue dashed line indicates 5 bp, the cutoff for small deletion length found in tumors with defective homologous recombination DNA repair. **F**) Cytolytic activity (CYT) of the immune infiltrate scores in high-risk-variant *CHEK2*-associated (TCGA), *CHEK2*-associated p.Ile157Thr variant (TCGA), *ATM*-associated (TCGA), *BRCA1*-associated (TCGA), *BRCA2*-associated (TCGA), and sporadic (ie, non-*BRCA1/BRCA2/ATM/CHEK2*) BCs (TCGA) subjected to RNA-sequencing. In all panels, *P* values relate to the comparisons between high-risk–variant *CHEK2*-associated BCs and other groups; Box plot markings from bottom to top: minimum value, first quartile, median, third quartile, maximum value. *P < 0.05; **P < 0.01; Mann-Whitney U test. TCGA = The Cancer Genome Atlas; WES = whole-exome sequencing.

Germline mutations affecting several DNA repair-related genes (eg, *BRCA1*, *BRCA2*, *PALB2*) have been associated with an increased BC risk, and these BCs often show genomic features of homologous recombination DNA repair deficiency (HRD) (14,16,17), including high large-scale state transitions (LSTs) scores, a dominant mutational signature 3, and long small deletion lengths. At variance with *BRCA1*- and *BRCA2*-associated BCs, but akin to *ATM*-associated BCs, only one (20%) of the TCGA high-risk–variant *CHEK2*-associated BCs displayed a high LST score (Figure 2C) and none of the five *CHEK2*-associated breast cancers subjected to WES harbored a dominant mutational signature 3 (Figure 2D;Supplementary Figure 7, available online), consistent with the results of Polak et al. (16) and Riaz et al. (14). In addition, the length of small deletions was statistically significantly smaller in high-risk–variant *CHEK2*-associated BCs than in *BRCA1*- and *BRCA2*-associated BC (Figure 2E). These observations are also consistent with recent functional genomics findings suggesting that *CHEK2* loss-of-function may not mediate response to poly adenosine diphosphate ribose polymerase inhibitors, an HR-directed therapy (18). Finally, high-risk–variant *CHEK2*-associated BCs displayed a lower level of cytolytic activity of the immune infiltrate cytolytic activity (CYT) score [19]) than *BRCA1*-associated BCs (Figure 2F), akin to *ATM*-associated BCs (11).

Taken together, our findings and those by Massink et al. (20) indicate that *CHEK2*-associated BCs are phenotypically and genomically distinct from *BRCA1*- and *BRCA2*-associated BCs, but similar to *ATM*-associated BCs in that these tumors are preferentially ER positive, lack genomic features suggestive of HRD, and rarely harbor *TP53* mutations. Akin to *ATM*-associated BCs (11), either the mechanism by which *CHEK2* loss of function contributes to BC development may be independent of the HR pathway or the genomics signatures may differ from those caused by the loss of function of canonical HR-related genes. BCs arising in the context of the low-risk *CHEK2* p.Ile157Thr germline variant differ from those in patients with *CHEK2* high-risk variants, with the latter having a higher frequency of LOH of the *CHEK2* wild-type allele and a more profound effect on CHEK2 protein expression. Therefore, the type of germline variant and additional biomarkers may be required for the optimal tailoring of therapies for *CHEK2* BC patients.

## Funding

This study was funded by the Breast Cancer Research Foundation and the Sarah Jenkins Fund. Research reported in this paper was supported in part by a Cancer Center Support Grant of the National Institutes of Health/National Cancer Institute (Grant No. P30CA008748).

## Notes

Affiliations of authors: Department of Pathology, (DM, RK, PS, SA, OC-B, DNB, HYW, BW, JSR-F), Department of Radiation Oncology, (XP, JS, SP, NR), and Department of Medicine (LN, MER), Memorial Sloan Kettering Cancer Center, New York, NY.

DM and RK contributed equally. DM, MER, BW, and JSR-F conceived the study. HYW and JSR-F performed the pathology review. RK, XP, PS, SA, and DNB performed bioinformatics analyses. DM, JS, OC-B, LN, MER, SP, NR, BW, and JSR-F interpreted the data. DM, RK, BW, and JSR-F wrote the first draft of the manuscript, which was edited and approved by all authors.

The content is solely the responsibility of the authors and does not necessarily represent the official views of the National Institutes of Health.

JSR-F reports personal and/or consultancy fees from VolitionRx, Page.AI, Goldman Sachs, Grail, Ventana Medical Systems and Genentech, outside the scope of the submitted work. The remaining authors have no conflicts of interest to declare.

## Supplementary Material

Supplementary_Data_pkz027
