# Supplementary material for: The Landscape of Somatic Genetic Alterations in Breast Cancers from CHEK2 Germline Mutation Carriers
Source: JNCI Cancer Spectr. 2019 Apr 27;3(2):pkz027. doi: 10.1093/jncics/pkz027 (PMC6649818; doi:10.1093/jncics/pkz027)
Supplement: Supplementary_Data_pkz027 [file supplementary_data_pkz027.pdf]

SUPPLEMENTARY MATERIALS

The landscape of somatic genetic alterations in breast cancers from *CHEK2* germline mutation carriers

Mandelker et al.

Supplementary Methods

Supplementary Figures 1-7

Supplementary Tables 1-3

SUPPLEMENTARY METHODS

Cases

Individuals with a *CHEK2* germline mutation were identified from the cohort of patients profiled using the Memorial Sloan Kettering Mutation Profiling of Actionable Cancer Targets (MSK-IMPACT) assay (n=4,740) who consented to germline analysis under an Institutional Review Board (IRB)-approved protocol at MSKCC. None of the CHEK2-associated breast cancers harbored germline alterations in any of 75 other genes associated with hereditary cancer susceptibility, including *BRCA1*, *BRCA2* or *ATM*. All germline *CHEK2* variants were reviewed by a board-certified molecular pathologist (D.M.) and classified according to the American College of Medical Genetics and Genomics (ACMG) criteria (1) as pathogenic (**Supplementary Table 1**). Surgical pathology reports and medical records were reviewed by one of the authors (D.M.) to establish size, grade, stage and hormone receptor status of the tumors included in this study. Estrogen receptor (ER) and HER2 status were assessed by immunohistochemistry and/or fluorescence *in situ* hybridization (FISH), following the American Society of Clinical Oncology/College of American Pathologists (ASCO/CAP) guideline recommendations (2,3). Additionally, breast cancers with *CHEK2* germline variants were obtained from The Cancer Genome Atlas (see below) (4).

Breast cancers from The Cancer Genome Atlas (TCGA) and International Genome Consortium (ICGC)

We obtained whole-exome and RNA-Sequencing data from breast cancers from *CHEK2* germline mutation carriers from The Cancer Genome Atlas (TCGA) breast cancer study (n=20) (4); data were downloaded from the GDC legacy archive website and compared to the results reported in Riaz et al. (5). Of these 20 cases, 5 had frameshift mutations classified as high-risk germline variants, and 15 carried the low-risk p.Ile157Thr missense *CHEK2* germline variant (**Supplementary Table 1**). We also obtained data from BRCA1- and BRCA2-associated breast cancers (**Supplementary Table 3**) and

sporadic breast cancer from TCGA (4) and the International Cancer Genome Consortium (ICGC) (6) as described previously (7), which were used for the comparison with CHEK2-associated breast cancers in this study.

Breast cancer samples with *ATM* germline mutations

We previously reported on the somatic genetics of 32 breast cancers with *ATM* germline pathogenic mutations (n=20, whole-exome sequencing; n=12, MSK-IMPACT sequencing) (7). These 32 ATM-associated breast cancers were used for the comparisons with CHEK2-associated breast cancers included in this study.

DNA extraction and MSK-IMPACT targeted sequencing

DNA was extracted from both formalin-fixed paraffin embedded tumor samples and matched normal blood, as described previously (8), and subjected to targeted massively parallel sequencing using the MSK-IMPACT assay. This assay specifically targets all exonic and selected intronic regions of 410-468 cancer genes, depending on test version, as previously described (8-11).

Bioinformatics analysis

Bioinformatics analyses of sequencing data followed best practice approaches and were essentially conducted as previously described (7,12). In brief, for MSK-IMPACT and whole-exome sequencing, reads were aligned to the reference human genome GRCh37 using the Burrows-Wheeler Aligner (BWA, v0.7.15) (13). Local realignment, duplicate removal and base quality score recalibration were performed using the Genome Analysis Toolkit (GATK, v3.1.1) (14). Somatic single nucleotide variants (SNVs) were identified using MuTect (v1.0) (15), small insertions and deletions (indels) using a combination of Strelka (v2.0.15) (16), VarScan2 (v2.3.7) (17), Lancet (v1.0.0) (18) and Scalpel (v0.5.3) (19), and further curated by manual inspection. SNVs and indels outside of the target regions were filtered out, as were SNVs and indels for which the variant allele fraction (VAF) in the tumor sample was

<5 times that of the paired normal VAF, and SNVs and indels found at >5% global minor allele frequency of dbSNP (build 137), as previously described (7,20). Somatic copy number alterations and loss of heterozygosity (LOH) were obtained using FACETS (21), as previously described (5,7,11,22). Somatic LOH of the wild-type allele of *CHEK2* germline mutations was classified as subclonal if the cancer cell fraction (see below) was lower than the tumor purity (delta ≥0.2) as determined by FACETS, as previously described (7). The cancer cell fractions (CCFs) of all mutations were computed using ABSOLUTE (v1.0.6)(20), as previously described (5,7,11,22). A mutation was classified as clonal if its probability of being clonal was >50% (23) or if the lower bound of the 95% confidence interval of its CCF was >90 (5,7,11,22). Mutations that did not meet the above criteria were considered subclonal (5,7,11,22). A combination of mutation function predictors (24) was employed to define the potential functional impact of each missense SNV, as previously described (5,7,11,22). Mutation hotspots were assigned according to Chang *et al*. (25).

Reverse-phase protein arrays (RPPAs)

RPPA data for TCGA samples were obtained from the TCPA database website (http://tcpaportal.org/tcpa). This database provides the RPPA protein abundance data of nearly 200 proteins and phosphoproteins (26). Level 3 data from the TCPA website were downloaded and used without any further normalization. The relative protein expression values were used to compare the CHEK2 protein levels in CHEK2-, BRCA1-, BRCA2-associated and sporadic BCs.

Assessment of genomics features of homologous recombination (HR) DNA repair deficiency

To define whether a given breast cancer would likely harbor defective HR DNA repair, we assessed large-scale state transition (LST) scores, mutational signatures and average length of small deletions.

LST scores were used calculated as previously described (5,27); in brief, a LST event was defined as a chromosomal breakpoint resulting in allelic imbalance between adjacent regions of at least 10Mb,

obtained after smoothing and filtering small-scale copy number variations (<3 Mb). LST scores were calculated only for samples with whole-exome sequencing data available, and a cut-off of 15 was employed to classify tumors into high LST (≥15) and low LST (<15) tumors as described previously (5,27).

To define the mutational signatures, we assessed the mutational context of synonymous and non-synonymous SNVs in the CHEK2-associated breast cancers subjected to whole-exome sequencing, as previously described (9), using deconstructSigs (28) at default settings with COSMIC signature metrics. Both synonymous and non-synonymous SNVs were used as input to calculate the mutational signatures as described previously (5,6,29). Mutational signatures were defined only for the samples with at least 20 SNVs.

The average length of small deletions in a cancer has been recently shown to correlate with defective HR DNA repair (6,29,30), in that the larger the average deletion length the likelier a tumor is to display defective HR DNA repair. We calculated the average length of small deletions for the breast cancers subjected to whole-exome sequencing in this study, and used a cut-off of 5 to define high (≥5) and low (<5) average deletion length tumor as previously described (6,29,30).

CYT Score

CYT score was used as a parameter to determine the cytolytic activity of the immune infiltrate (CYT). It is calculated as geometric mean expression of two key cytolytic effectors, namely GZMA (Granzyme A) and PRF1 (Perforin 1) (31), which are upregulated upon CD8+ T cell activation. CYT scores were calculated for TCGA samples for which RNA-sequencing data was available as previously described (7).

Statistical analyses

Comparisons of the number of somatic mutations between high-risk variant CHEK2-associated breast cancers and low-risk p.Ile157Thr CHEK2-associated breast cancers and those of ATM-, BRCA1- and BRCA2-associated breast cancers and sporadic (i.e. non-BRCA1/BRCA2/ATM/CHEK2) breast cancers were performed using the Mann-Whitney U test. Frequencies of mutations affecting single genes or the hormone receptor status were compared between high-risk CHEK2-associated, low-risk p.Ile157Thr CHEK2-associated breast cancers and ATM-, BRCA1- and BRCA2-associated breast cancers and sporadic (i.e. non-BRCA1/BRCA2/ATM/CHEK2) breast cancers using Fisher's exact test. Two tailed *P* values <0.05 were considered statistically significant. Statistical analyses were performed using R v3.1.2 and Prism 7.

SUPPLEMENTARY REFERENCES

1. Richards S, Aziz N, Bale S, et al. Standards and guidelines for the interpretation of sequence variants: a joint consensus recommendation of the American College of Medical Genetics and Genomics and the Association for Molecular Pathology. *Genet Med*. 2015;17(5):405-424.
2. Hammond ME, Hayes DF, Dowsett M, et al. American Society of Clinical Oncology/College Of American Pathologists guideline recommendations for immunohistochemical testing of estrogen and progesterone receptors in breast cancer. *J Clin Oncol*. 2010;28(16):2784-2795.
3. Wolff AC, Hammond MEH, Allison KH, et al. Human Epidermal Growth Factor Receptor 2 Testing in Breast Cancer: American Society of Clinical Oncology/College of American Pathologists Clinical Practice Guideline Focused Update. *J Clin Oncol*. 2018;36(20):2105-2122.
4. Cancer Genome Atlas Network. Comprehensive molecular portraits of human breast tumours. *Nature*. 2012;490(7418):61-70.
5. Riaz N, Blecua P, Lim RS, et al. Pan-cancer analysis of bi-allelic alterations in homologous recombination DNA repair genes. *Nat Commun*. 2017;8(1):857.
6. Nik-Zainal S, Davies H, Staaf J, et al. Landscape of somatic mutations in 560 breast cancer whole-genome sequences. *Nature*. 2016;534(7605):47-54.
7. Weigelt B, Bi R, Kumar R, et al. The Landscape of Somatic Genetic Alterations in Breast Cancers From ATM Germline Mutation Carriers. *J Natl Cancer Inst*. 2018; 10.1093/jnci/djy028.
8. Zehir A, Benayed R, Shah RH, et al. Mutational landscape of metastatic cancer revealed from prospective clinical sequencing of 10,000 patients. *Nat Med*. 2017;23(6):703-713.
9. Cheng DT, Mitchell TN, Zehir A, et al. Memorial Sloan Kettering-Integrated Mutation Profiling of Actionable Cancer Targets (MSK-IMPACT): A Hybridization Capture-Based Next-Generation Sequencing Clinical Assay for Solid Tumor Molecular Oncology. *J Mol Diagn*. 2015;17(3):251-264.
10. Schultheis AM, Ng CK, De Filippo MR, et al. Massively Parallel Sequencing-Based Clonality Analysis of Synchronous Endometrioid Endometrial and Ovarian Carcinomas. *J Natl Cancer Inst*. 2016;108(6):djv427.

11. Geyer FC, Li A, Papanastasiou AD, et al. Recurrent hotspot mutations in HRAS Q61 and PI3K-AKT pathway genes as drivers of breast adenomyoepitheliomas. *Nat Commun*. 2018;9(1):1816.

12. DeLair DF, Burke KA, Selenica P, et al. The genetic landscape of endometrial clear cell carcinomas. *J Pathol*. 2017;243(2):230-241.

13. Li H, Durbin R. Fast and accurate short read alignment with Burrows-Wheeler transform. *Bioinformatics*. 2009;25(14):1754-1760.

14. McKenna A, Hanna M, Banks E, et al. The Genome Analysis Toolkit: a MapReduce framework for analyzing next-generation DNA sequencing data. *Genome Res*. 2010;20(9):1297-1303.

15. Cibulskis K, Lawrence MS, Carter SL, et al. Sensitive detection of somatic point mutations in impure and heterogeneous cancer samples. *Nat Biotechnol*. 2013;31(3):213-219.

16. Saunders CT, Wong WS, Swamy S, et al. Strelka: accurate somatic small-variant calling from sequenced tumor-normal sample pairs. *Bioinformatics*. 2012;28(14):1811-1817.

17. Koboldt DC, Zhang Q, Larson DE, et al. VarScan 2: somatic mutation and copy number alteration discovery in cancer by exome sequencing. *Genome Res*. 2012;22(3):568-576.

18. Narzisi G, Corvelo A, Arora K, et al. Genome-wide somatic variant calling using localized colored de Bruijn graphs. *Communications Biology*. 2018;1(1):20.

19. Narzisi G, O'Rawe JA, Iossifov I, et al. Accurate de novo and transmitted indel detection in exome-capture data using microassembly. *Nat Methods*. 2014;11(10):1033-1036.

20. Ng CKY, Piscuoglio S, Geyer FC, et al. The Landscape of Somatic Genetic Alterations in Metaplastic Breast Carcinomas. *Clin Cancer Res*. 2017;23(14):3859-3870.

21. Shen R, Seshan VE. FACETS: allele-specific copy number and clonal heterogeneity analysis tool for high-throughput DNA sequencing. *Nucleic Acids Res*. 2016;44(16):e131.

22. Pareja F, Brandes AH, Basili T, et al. Loss-of-function mutations in ATP6AP1 and ATP6AP2 in granular cell tumors. *Nat Commun*. 2018;9(1):3533.

23. Landau DA, Carter SL, Stojanov P, et al. Evolution and impact of subclonal mutations in chronic lymphocytic leukemia. *Cell*. 2013;152(4):714-726.

24. Martelotto LG, Ng CK, De Filippo MR, et al. Benchmarking mutation effect prediction algorithms using functionally validated cancer-related missense mutations. *Genome Biol*. 2014;15(10):484.
25. Chang MT, Bhattarai TS, Schram AM, et al. Accelerating Discovery of Functional Mutant Alleles in Cancer. *Cancer Discov*. 2018;8(2):174-183.
26. Li J, Lu Y, Akbani R, et al. TCPA: a resource for cancer functional proteomics data. *Nat Methods*. 2013;10(11):1046-1047.
27. Popova T, Manie E, Rieunier G, et al. Ploidy and large-scale genomic instability consistently identify basal-like breast carcinomas with BRCA1/2 inactivation. *Cancer Res*. 2012;72(21):5454-5462.
28. Rosenthal R, McGranahan N, Herrero J, et al. DeconstructSigs: delineating mutational processes in single tumors distinguishes DNA repair deficiencies and patterns of carcinoma evolution. *Genome Biol*. 2016;1731.
29. Davies H, Glodzik D, Morganella S, et al. HRDetect is a predictor of BRCA1 and BRCA2 deficiency based on mutational signatures. *Nat Med*. 2017;23(4):517-525.
30. Alexandrov L, Kim J, Haradhvala NJ, et al. The Repertoire of Mutational Signatures in Human Cancer. *bioRxiv*. 2018; 10.1101/322859.
31. Rooney MS, Shukla SA, Wu CJ, et al. Molecular and genetic properties of tumors associated with local immune cytolytic activity. *Cell*. 2015;160(1-2):48-61.

Supplementary Figure 1

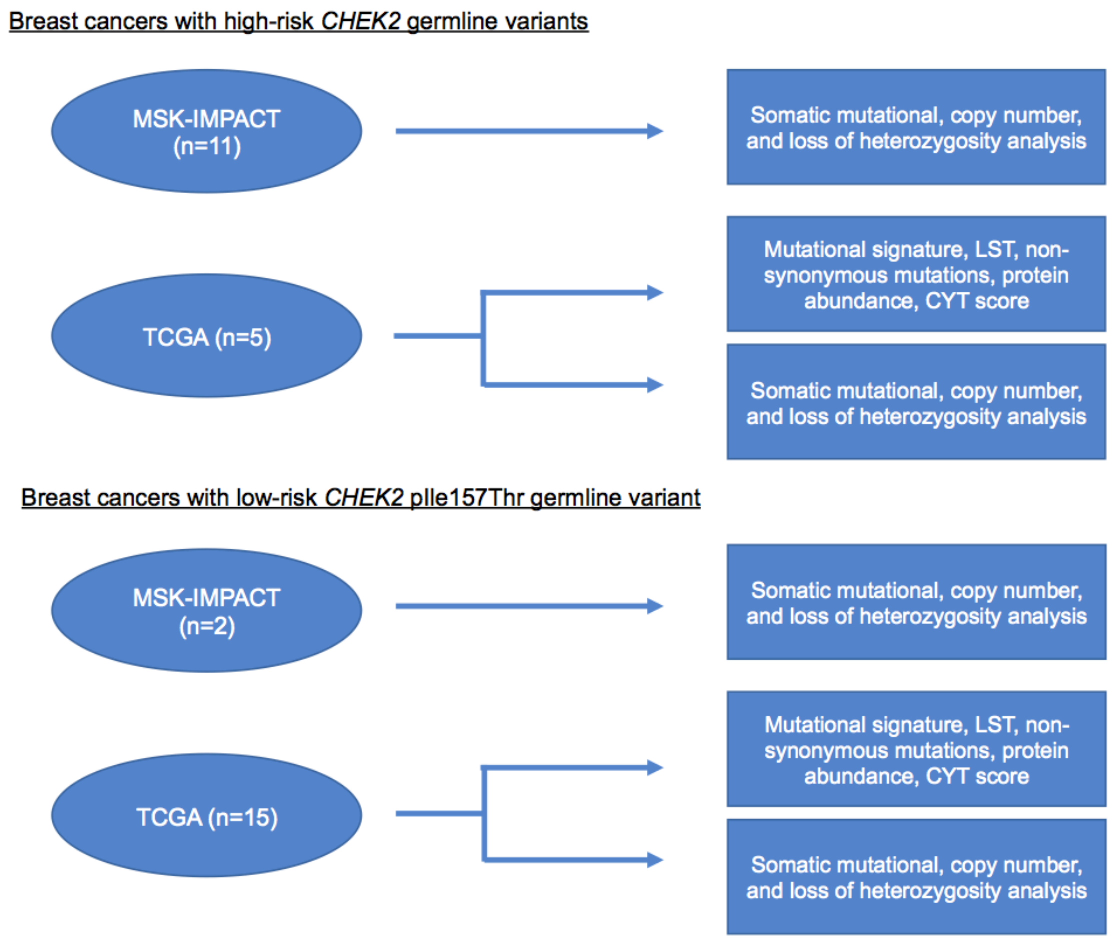

Supplementary Figure 1. Schematic representation of the CHEK2 breast cancers and the analyses performed in this study.

Top, depiction of the high-risk-variant CHEK2-associated breast cancers subjected to MSK-IMPACT sequencing, and to whole-exome sequencing, RNA-sequencing and reverse phase protein arrays (RPPA) from The Cancer Genome Atlas (TCGA) (left), and the respective analyses performed (right). Bottom, depiction of the low-risk p.Ile157Thr variant CHEK2-associated breast cancers subjected to MSK-IMPACT sequencing (top), and to whole-exome sequencing, RNA-sequencing and reverse phase protein arrays (RPPA) from The Cancer Genome Atlas (TCGA) (left), and the respective analyses performed (right).

Supplementary Figure 2

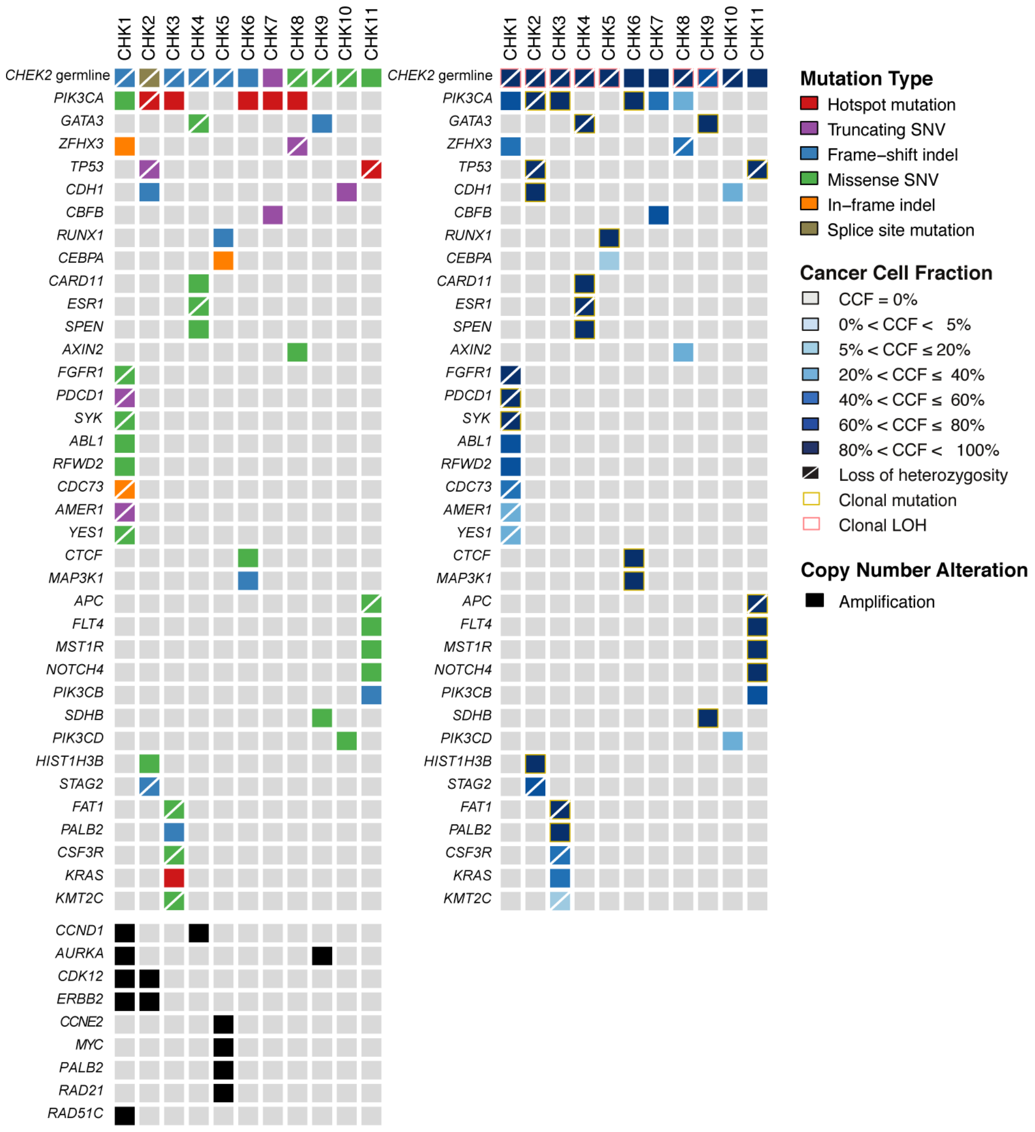

Supplementary Figure 2. Somatic genetic alterations in high-risk variant CHEK2-associated breast cancers subjected to MSK-IMPACT sequencing (n=11).

Non-synonymous somatic mutations and amplifications and homozygous deletions detected by MSK-IMPACT are shown in left panel and cancer cell fraction for each mutation is show in the right panel. Clonal loss of heterozygosity (LOH) of the *CHEK2* wild-type allele and clonal mutations are highlighted by yellow and red boxes, respectively. Indel, small insertion/deletion; SNV, single nucleotide variant.

Supplementary Figure 3

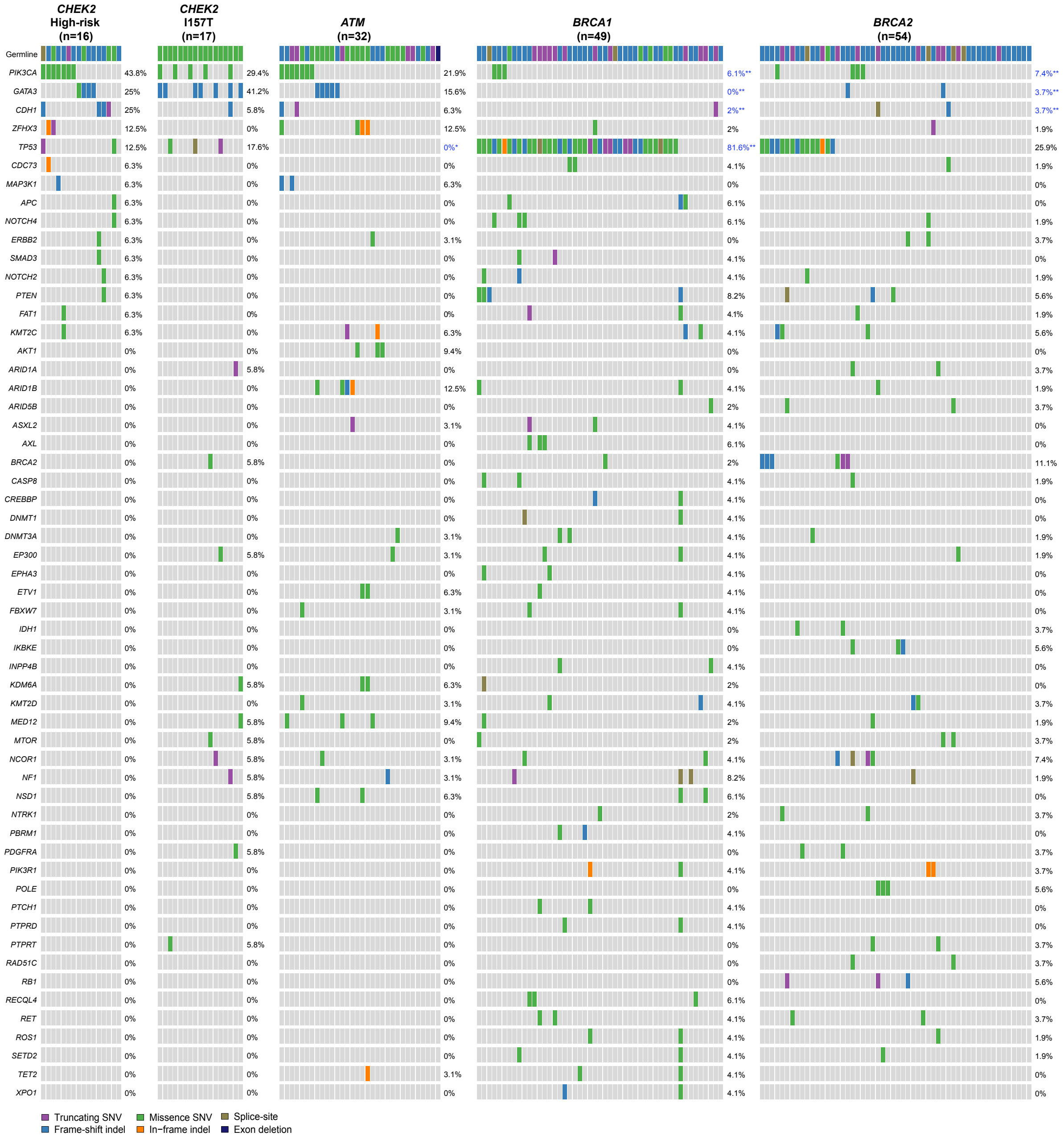

Supplementary Figure 3. Comparison of genes recurrently mutated in high-risk CHEK2-, low-risk p.Ile157Thr CHEK2-, ATM-, BRCA1- and BRCA2-associated breast cancers.
The repertoires of non-synonymous somatic mutations were retrieved for high-risk pathogenic CHEK2- (this study and TCGA), low-risk p.Ile157Thr CHEK2- (this study and TCGA), ATM- (Weigelt et al. and TCGA), BRCA1- (TCGA and ICGC) and BRCA2- (TCGA and ICGC). Only genes recurrently mutated in at least two samples and included in the MSK-IMPACT panel of 410 genes are depicted in the heatmap. Top row represents the germline mutation and color represent type of mutation type as shown in legend. *P < 0.05; **P < 0.01; Two-sided Mann-Whitney U test.

Supplementary Figure 4

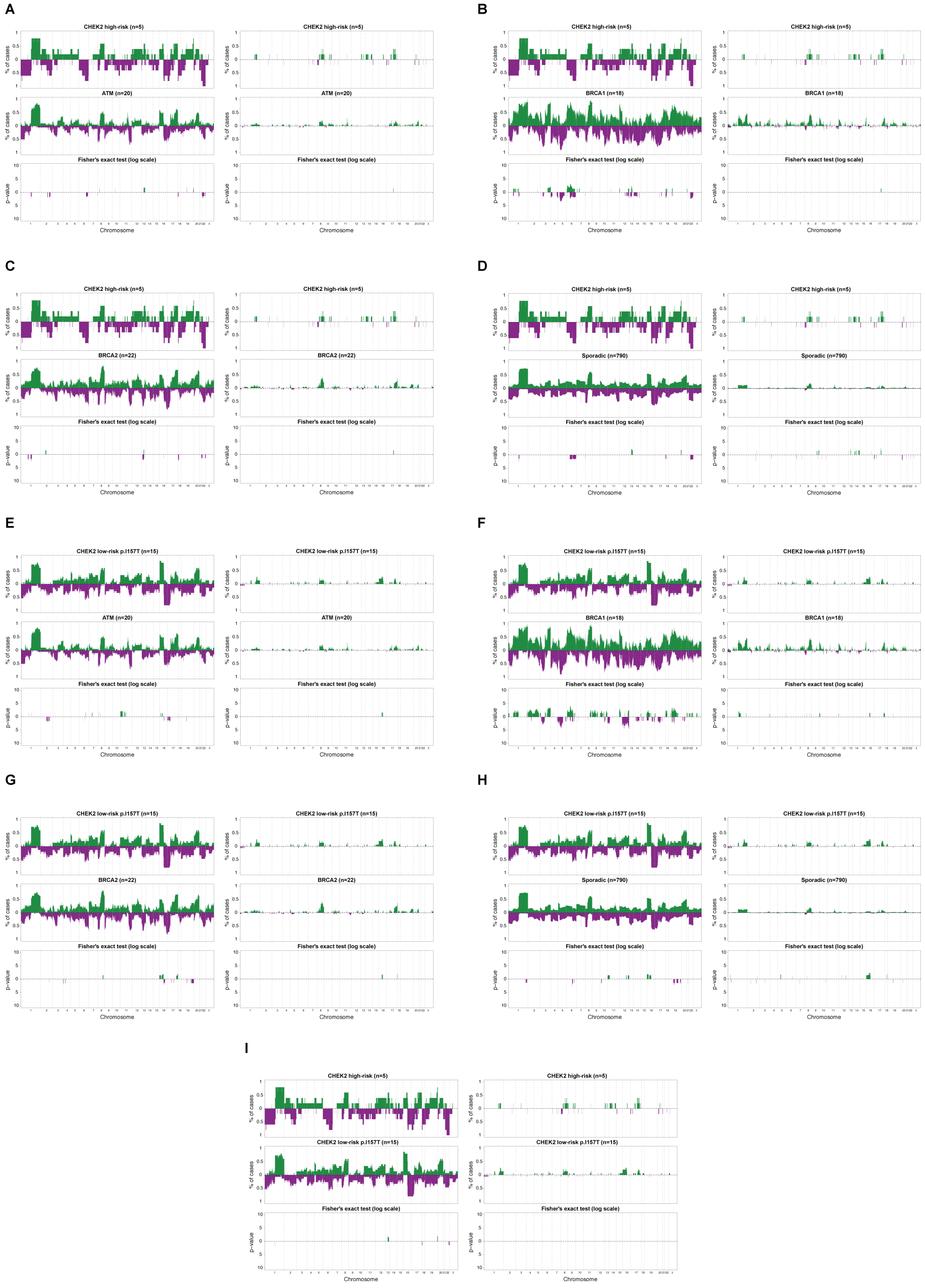

Supplementary Figure 4. Comparison of the frequency of gene copy number alterations in high-risk CHEK2-, low-risk p.Ile157Thr CHEK2-, ATM-, BRCA1- and BRCA2-associated breast cancers and sporadic (i.e. non-BRCA1/BRCA2/ATM/CHEK2) breast cancers.

The frequencies of gene copy number gains and losses (left panels) and amplifications and homozygous deletions (right panels) in high-risk pathogenic-variant CHEK2-associated breast cancers

were compared with those of (A) ATM-, (B) BRCA1-, (C) BRCA2-associated, (D) sporadic breast cancers. The frequencies of gene copy number gains and losses (left panels) and amplifications and homozygous deletions (right panels) in low-risk p.Ile157Thr CHEK2-associated breast cancers were compared with those of (E) ATM-, (F) BRCA1-, (G) BRCA2-associated, (H) sporadic breast cancers. The frequencies of gene copy number gains and losses (left panels) and amplifications and homozygous deletions (right panels) in high-risk-variant CHEK2-associated breast cancers and low-risk p.Ile157Thr CHEK2-associated breast cancers (I). In the frequency graphs, the genomic position is presented on the x-axis, and the frequency of the copy number alterations affecting a specific locus is depicted on the y-axis. In the bottom graph, the $-Log_{10}$ of the P-value is presented according to the genomic position of the gene copy number alteration. Differences were defined using a two-tailed multi-Fisher's exact test.

Supplementary Figure 5

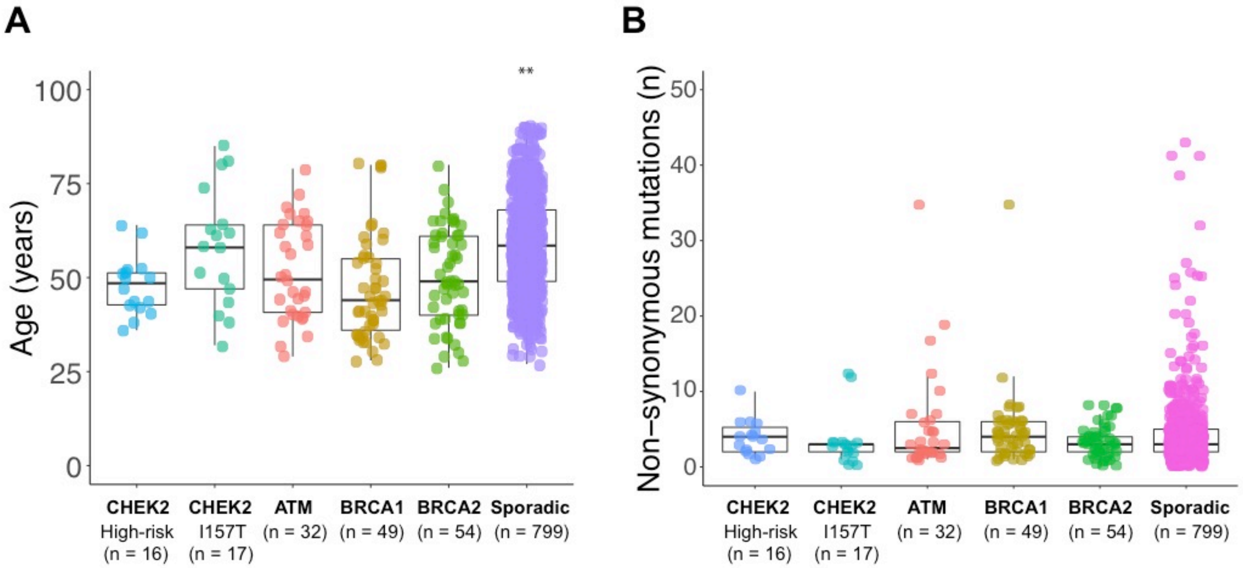

Supplementary Figure 5. Comparative analysis of high-risk CHEK2-, low-risk p.Ile157Thr CHEK2-, ATM-, BRCA1- and BRCA2-associated breast cancers and sporadic (i.e. non-BRCA1/BRCA2/ATM/CHEK2) breast cancers.

Age of onset (A) in high-risk variant CHEK2- (this study and TCGA), low-risk p.Ile157Thr CHEK2- (this study and TCGA), ATM- (Weigelt et al. and TCGA), BRCA1- (TCGA and ICGC) and BRCA2-(TCGA and ICGC) associated breast cancers and sporadic (i.e. non-BRCA1/BRCA2/ATM/CHEK2; TCGA) breast cancers. Number of non-synonymous somatic mutations affecting genes included in 410 genes of MSK-IMPACT assay in high-risk variant CHEK2- (this study and TCGA), low-risk p.Ile157Thr CHEK2- (this study and TCGA), ATM- (Weigelt et al. and TCGA), BRCA1- (TCGA and ICGC) and BRCA2- (TCGA and ICGC) associated breast cancers and sporadic (i.e. non-BRCA1/BRCA2/ATM/CHEK2; TCGA) breast cancers. **P < 0.01; Two-sided Mann-Whitney U test.

Supplementary Figure 6

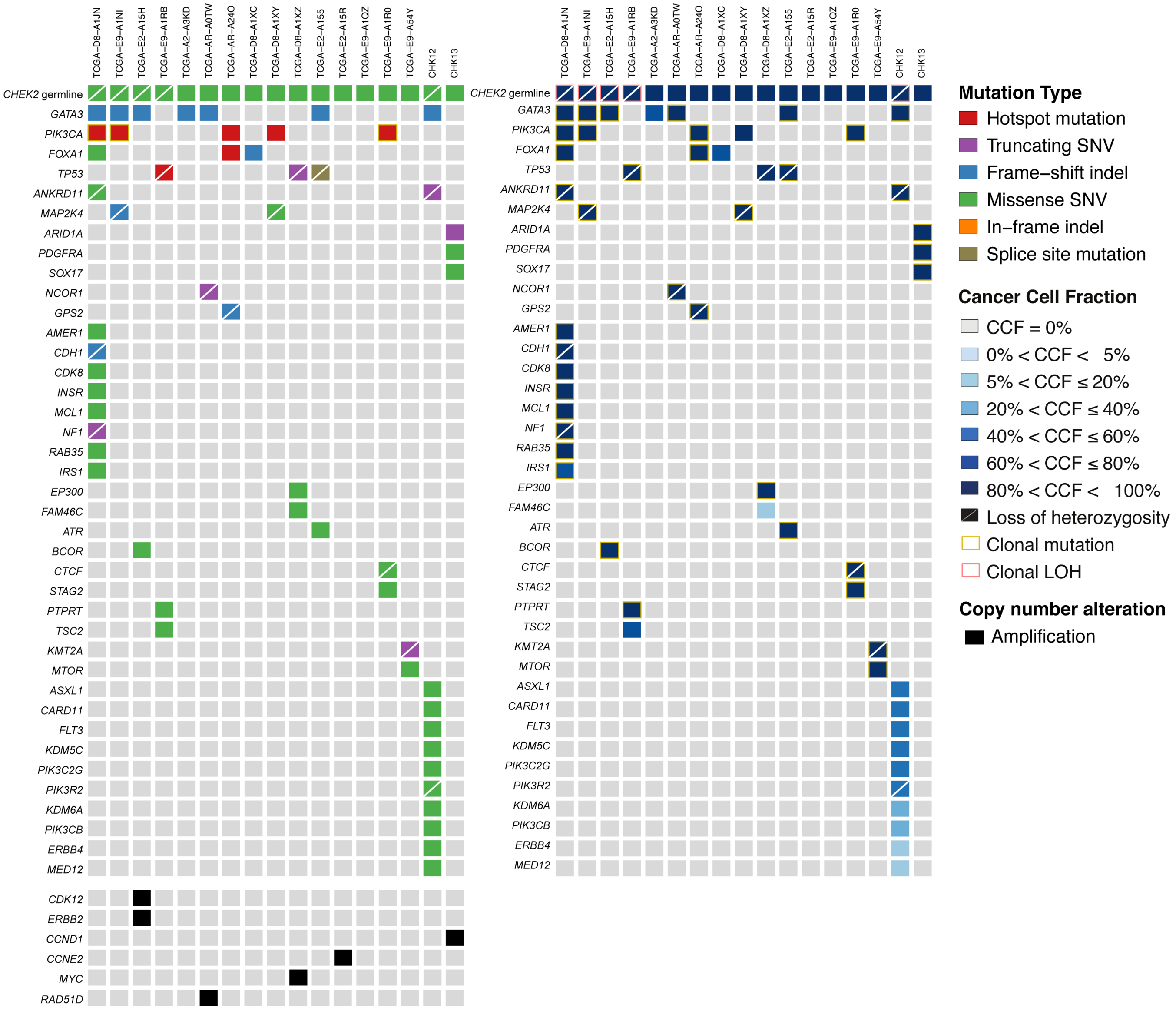

Supplementary Figure 6. Repertoire of somatic genetic alterations found in CHEK2-associated breast cancers harboring the low-risk p.Ile157Thr germline variant.

Non-synonymous mutations and amplification and homozygous deletions in breast cancers from patients with a low-risk p.Ile157Thr germline variant from TCGA (n=15) and sequenced in this study using the MSK-IMPACT assay (n=2). Non-synonymous somatic mutations, amplification and homozygous deletions affecting genes included in the list of 410 genes of MSK-IMPACT are shown in left panel, and the cancer cell fraction for each mutation is show in the right panel. Clonal loss of heterozygosity (LOH) of the *CHEK2* wild-type allele and clonal mutations are highlighted by yellow and red boxes, respectively. Indel, small insertion/deletion; SNV, single nucleotide variant.

Supplementary Figure 7

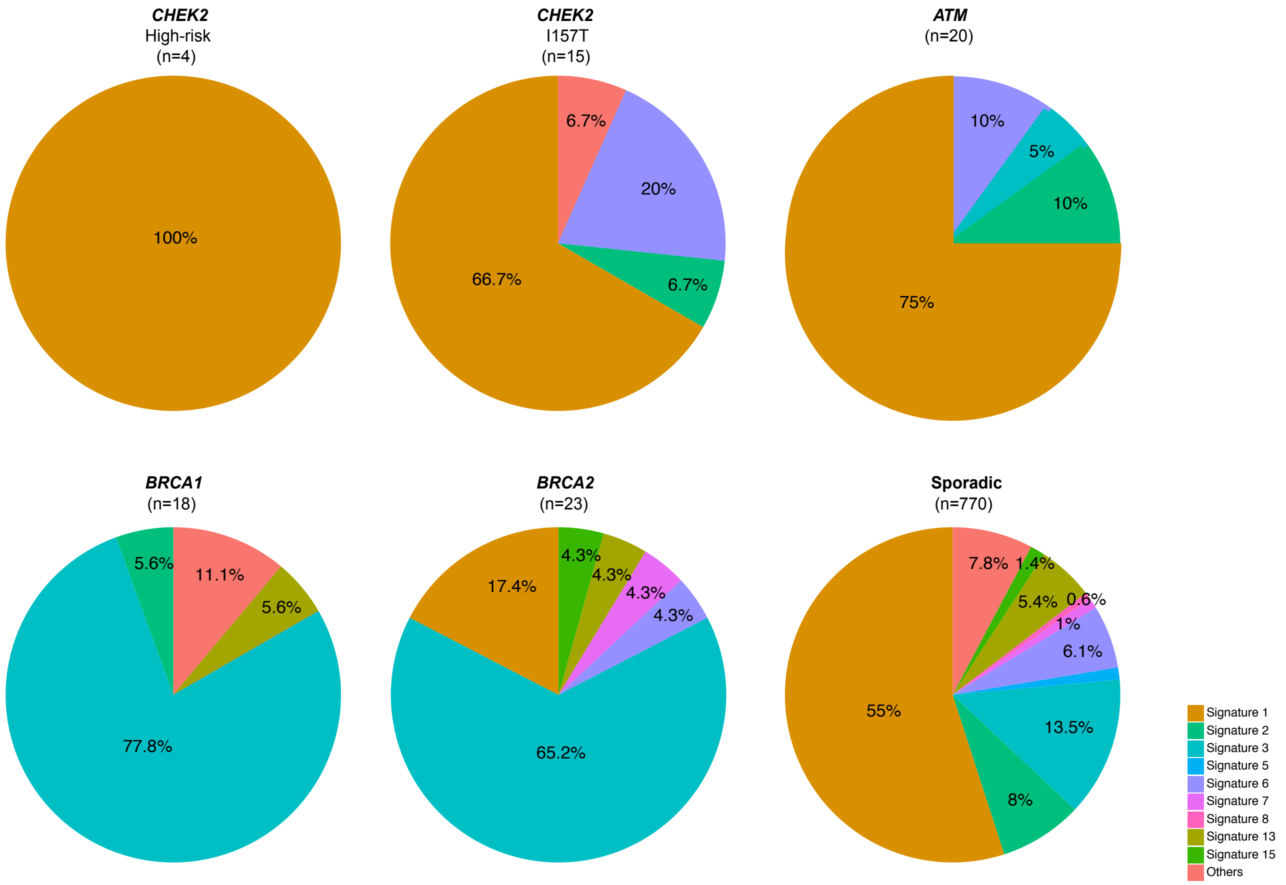

Supplementary Figure 7. Mutational signatures in high-risk-variant CHEK2-, low-risk p.Ile157Thr CHEK2-, ATM-, BRCA1- and BRCA2-associated breast cancers and sporadic (i.e. non-BRCA1/BRCA2/ATM/CHEK2) breast cancers.

Mutational signatures as defined by DeconstructSigs in high-risk-variant CHEK2- (TCGA), low-risk p.Ile157Thr CHEK2- (TCGA), ATM- (Weigelt et al. and TCGA), BRCA1- (TCGA) and BRCA2- (TCGA) associated breast cancers and sporadic (i.e. non-BRCA1/BRCA2/ATM/CHEK2; TCGA) breast cancers subjected to whole-exome sequencing. The dominant mutational signatures found in a given group of breast cancers are shown. Synonymous and non-synonymous mutations were included in the analysis.

SUPPLEMENTARY TABLES

Supplementary Table 1. Clinico-pathologic information of the CHEK2-associated breast cancers analyzed in this study.

ID	Age at Diagnosis (years)	ER	HER2	Stage	Tumor Size (cm)	Histologic Subtype	*CHEK2* Germline mutation	Mutation Type	Sequencing Type
CHK1	36	Negative	Positive	IV	NA	IDC-NST	c.1100delC; p.Thr367Metfs*15	Frameshift Deletion	MSK-IMPACT
CHK2	50	Negative	Positive	IV	1.1	IDC-NST	c.444+1G>A	Splice-site	MSK-IMPACT
CHK3	44	Positive	Negative	IV	1.7	IDC-NST	c.1100delC; p.Thr367Metfs*15	Frameshift Deletion	MSK-IMPACT
CHK4	42	Positive	Negative	IV	2.6	Mixed IDC/ILC	c.1100delC; p.Thr367Metfs*15	Frameshift Deletion	MSK-IMPACT
CHK5	52	Positive	Negative	IV	0.9	IDC-NST	c.1100delC; p.Thr367Metfs*15	Frameshift Deletion	MSK-IMPACT
CHK6	64	Positive	Negative	1A	0.4	IDC-NST	c.1100delC; p.Thr367Metfs*15	Frameshift Deletion	MSK-IMPACT
CHK7	51	Positive	Negative	IV	1.6	IDC-NST	c.283C>T; p.Arg95*	Nonsense	MSK-IMPACT
CHK8	51	Positive	Negative	IV	1.9	ILC	c.1283C>T; p.Ser428Phe	Missense	MSK-IMPACT
CHK9	40	Positive	Negative	IV	2.6	IDC-NST	c.1283C>T; p.Ser428Phe	Missense	MSK-IMPACT
CHK10	47	Positive	Negative	IV	1.3	ILC	c.1283C>T; p.Ser428Phe	Missense	MSK-IMPACT
CHK11	38	Positive	Negative	IV	NA	IDC-NST	c.1283C>T; p.Ser428Phe	Missense	MSK-IMPACT
CHK12	32	Positive	Negative	IV	NA	IDC-NST	c.470T>C; p.Ile157Thr	Missense	MSK-IMPACT
CHK13	62	Positive	Negative	IV	1.6	IDC-NST	c.470T>C; p.Ile157Thr	Missense	MSK-IMPACT
TCGA-BH-A1FM	44	Positive	Negative	IIIA	NA	IDC-NST	c.952delC; p.Arg318fs	Frameshift Deletion	WES (TCGA)
TCGA-A2-A0T6	50	Positive	Negative	IIB	NA	ILC	c.705_706insA; p.Ser235_Glu236fs	Frameshift Deletion	WES (TCGA)
TCGA-A7-A4SC	62	Positive	Negative	IIB	NA	ILC	c.437delC; p.Thr146fs	Frameshift Deletion	WES (TCGA)
TCGA-A8-A08J	52	Positive	Positive	IV	NA	IDC-NST	c.437delC; p.Thr146fs	Frameshift Deletion	WES (TCGA)
TCGA-E9-A1RI	43	NA	Negative	IIIA	NA	IDC-NST	c.437delC; p.Thr146fs	Frameshift Deletion	WES (TCGA)
TCGA-A2-A3KD	47	Positive	Negative	IIIA	NA	Mixed Mucinous	c.470T>C; p.Ile157Thr	Missense	WES (TCGA)
TCGA-AR-A0TW	50	Positive	Negative	IIIA	NA	IDC-NST	c.470T>C; p.Ile157Thr	Missense	WES (TCGA)
TCGA-AR-A24O	43	Positive	Negative	IIIA	NA	IDC-NST	c.470T>C; p.Ile157Thr	Missense	WES (TCGA)
TCGA-D8-A1JN	80	Positive	Negative	IIIC	NA	ILC	c.470T>C; p.Ile157Thr	Missense	WES (TCGA)
TCGA-D8-A1XC	85	Positive	Negative	IIIB	NA	Mixed Mucinous	c.470T>C; p.Ile157Thr	Missense	WES (TCGA)
TCGA-D8-A1XY	74	Positive	Negative	IIA	NA	IDC-NST	c.470T>C; p.Ile157Thr	Missense	WES (TCGA)
TCGA-D8-A1XZ	81	Positive	Negative	IIIA	NA	IDC-NST	c.470T>C; p.Ile157Thr	Missense	WES (TCGA)
TCGA-E2-A155	58	Positive	Negative	IIB	NA	IDC-NST	c.470T>C; p.Ile157Thr	Missense	WES (TCGA)
TCGA-E2-A15H	38	Positive	Positive	IIA	NA	IDC-NST	c.470T>C; p.Ile157Thr	Missense	WES (TCGA)
TCGA-E2-A15R	64	Positive	Negative	IIA	NA	IDC-NST	c.470T>C; p.Ile157Thr	Missense	WES (TCGA)
TCGA-E9-A1NI	51	Positive	Negative	IIA	NA	IDC-NST	c.470T>C; p.Ile157Thr	Missense	WES (TCGA)
TCGA-E9-A1QZ	61	NA	Negative	IIA	NA	IDC-NST	c.470T>C; p.Ile157Thr	Missense	WES (TCGA)
TCGA-E9-A1R0	58	NA	Negative	IIA	NA	IDC-NST	c.470T>C; p.Ile157Thr	Missense	WES (TCGA)
TCGA-E9-A1RB	40	NA	Negative	IIA	NA	IDC-NST	c.470T>C; p.Ile157Thr	Missense	WES (TCGA)
TCGA-E9-A54Y	63	Positive	Negative	IIIA	NA	ILC	c.470T>C; p.Ile157Thr	Missense	WES (TCGA)

ER, estrogen receptor; IDC-NST, invasive ductal carcinoma of no special type; ILC, invasive lobular carcinoma; NA, not available; WES, whole-exome sequencing.

Supplementary Table 2. Non-synonymous somatic mutations identified in CHEK2-associated breast cancers subjected to targeted massively parallel sequencing (MSK-IMPACT) or whole-exome sequencing.

Targeted massively parallel sequencing (MSK-IMPACT)

Sample ID	Chromosome	Position	Reference Allele	Alternate Allele	Gene	Amino Acid Change	Mutation Type	Hotspot	LOH	Tumor MAF	Normal MAF	Tumor Depth (X)	Normal Depth (X)	Cancer Cell Fraction	Probability Mutation is Clonal	95% CI High	95% CI Low	Clonal/ Subclonal	Pathogenicity	Mutation Taster	PROVEAN	FATHMM	CHASM	Cancer Gene Census	Kandoth et. al.	Lawrence et. al.
CHK1	23	63410556	G	A	AMER1	p.R871*	Nonsense_Mutation	FALSE	loh	0.051212938	0.00625	742	640	0.26	0	0.342993451	0.18266129	Subclonal	likely_pathogenic	D	.	.	.	TRUE	FALSE	FALSE
CHK1	18	751762	C	G	YES1	p.R105T	Missense_Mutation	FALSE	loh	0.054216867	0	498	395	0.27	0	0.384139589	0.182805252	Subclonal	likely_pathogenic	D	D	T	Driver	FALSE	FALSE	FALSE
CHK1	1	176050364	G	C	RFWD2	p.R401G	Missense_Mutation	FALSE	.	0.078145695	0.0025	755	400	0.7	0.02327	0.888652568	0.544822038	Subclonal	passenger	D	D	T	Passenger	FALSE	FALSE	FALSE
CHK1	16	72822012	TGCTGCTGCTGCTGTAGTTGCC	T	ZFHX3	p.R3381_Q3387del	In_Frame_Del	FALSE	.	0.081850534	0.039660057	562	353	0.41	0	0.532746625	0.304695139	Subclonal	passenger	N	D	.	.	TRUE	FALSE	TRUE
CHK1	1	193099325	AGAAGACCTG	A	CDC73	p.R87_D90delinsN	In_Frame_Del	FALSE	loh	0.097359736	0	606	313	0.49	0	0.613340343	0.376264288	Subclonal	likely_pathogenic	D	D	.	.	TRUE	FALSE	FALSE
CHK1	9	133760322	C	G	ABL1	p.S882C	Missense_Mutation	FALSE	.	0.108991826	0	734	339	0.65	0.00017	0.797155225	0.525822794	Subclonal	passenger	D	N	T	Passenger	TRUE	FALSE	FALSE
CHK1	8	38287283	A	T	FGFR1	p.V125E	Missense_Mutation	FALSE	loh	0.119897959	0	784	472	0.84	0.30938	0.975582698	0.687256818	Subclonal	passenger	N	N	T	Passenger	TRUE	FALSE	FALSE
CHK1	3	178951957	G	A	PIK3CA	p.M1004I	Missense_Mutation	FALSE	.	0.157289003	0	782	416	0.79	0.02791	0.916872429	0.662427714	Subclonal	likely_pathogenic	D	D	T	Driver	TRUE	TRUE	TRUE
CHK1	2	242793386	G	A	PDCD1	p.R231*	Nonsense_Mutation	FALSE	loh	0.331395349	0	516	384	1	0.03742	1	0.942201095	Clonal	likely_pathogenic	D	.	.	.	FALSE	FALSE	FALSE
CHK1	9	93641094	G	C	SYK	p.M480I	Missense_Mutation	FALSE	loh	0.363387978	0	732	366	1	0.88768	1	0.974166226	Clonal	likely_pathogenic	D	N	D	Driver	TRUE	FALSE	FALSE
CHK10	1	9783230	T	C	PIK3CD	p.I825T	Missense_Mutation	FALSE	.	0.028481013	0.002336449	632	428	0.3	0	0.462814076	0.185337485	Subclonal	likely_pathogenic	D	D	D	Passenger	FALSE	FALSE	FALSE
CHK10	16	68867262	G	T	CDH1	p.G837*	Nonsense_Mutation	FALSE	.	0.029268293	0	615	386	0.31	0	0.475663222	0.190906782	Subclonal	likely_pathogenic	D	.	.	.	TRUE	TRUE	TRUE
CHK11	3	138413709	C	CG	PIK3CB	p.R604Pfs*29	Frame_Shift_Ins	FALSE	.	0.131894484	0.014018692	417	214	0.74	0.10318	0.927002401	0.570921783	Subclonal	passenger	.	.	.	.	FALSE	FALSE	FALSE
CHK11	5	112177466	G	A	APC	p.D2059N	Missense_Mutation	FALSE	loh	0.190944882	0.003448276	508	290	1	0.90815	1	0.837984718	Clonal	likely_pathogenic	N	N	D	Driver	TRUE	TRUE	TRUE
CHK11	5	180045918	C	G	FLT4	p.E951D	Missense_Mutation	FALSE	.	0.192139738	0	229	160	0.88	0.66479	1	0.660498763	Clonal	likely_pathogenic	D	N	D	Passenger	TRUE	FALSE	FALSE
CHK11	6	32169981	G	T	NOTCH4	p.D1209E	Missense_Mutation	FALSE	.	0.195652174	0	368	318	0.9	0.69088	1	0.718098292	Clonal	likely_pathogenic	D	D	D	Passenger	FALSE	FALSE	FALSE
CHK11	3	49924886	A	G	MST1R	p.Y1353H	Missense_Mutation	FALSE	.	0.479487179	0	390	361	1	0.9213	1	0.971415511	Clonal	passenger	D	D	T	Passenger	FALSE	FALSE	FALSE
CHK11	17	7577581	A	G	TP53	p.Y234H	Missense_Mutation	TRUE	loh	0.900262467	0	381	277	1	0	1	0.986974408	Clonal	likely_pathogenic	D	D	D	Passenger	TRUE	TRUE	TRUE
CHK12	2	212286785	C	A	ERBB4	p.A971S	Missense_Mutation	FALSE	.	0.032663317	0	398	232	0.11	0	0.172680634	0.056671135	Subclonal	passenger	D	N	T	Passenger	TRUE	TRUE	FALSE
CHK12	23	70339283	G	T	MED12	p.D54Y	Missense_Mutation	FALSE	.	0.04691358	0.001883239	405	531	0.1	0	0.155786476	0.061941322	Subclonal	passenger	D	D	T	Passenger	TRUE	FALSE	TRUE
CHK12	3	138402603	G	C	PIK3CB	p.S781C	Missense_Mutation	FALSE	.	0.086111111	0	360	315	0.28	0	0.380471454	0.192595501	Subclonal	likely_pathogenic	D	D	D	Passenger	FALSE	FALSE	FALSE
CHK12	23	44921916	A	G	KDM6A	p.R484G	Missense_Mutation	FALSE	.	0.125	0	464	399	0.4	0	0.505100586	0.310762196	Subclonal	passenger	D	D	T	Passenger	TRUE	TRUE	TRUE
CHK12	20	31021452	A	G	ASXL1	p.E484G	Missense_Mutation	FALSE	.	0.131034483	0	435	324	0.55	0	0.696171801	0.42796409	Subclonal	passenger	N	N	T	Passenger	TRUE	TRUE	TRUE
CHK12	19	18273832	T	C	PIK3R2	p.F389L	Missense_Mutation	FALSE	loh	0.146263911	0	629	495	0.47	0	0.562360058	0.383897063	Subclonal	likely_pathogenic	D	D	D	Passenger	FALSE	FALSE	FALSE
CHK12	13	28626758	C	A	FLT3	p.D180Y	Missense_Mutation	FALSE	.	0.147826087	0.006289308	345	318	0.48	0	0.604794519	0.363069104	Subclonal	passenger	D	D	T	Passenger	TRUE	TRUE	TRUE
CHK12	7	2974191	G	C	CARD11	p.Q472E	Missense_Mutation	FALSE	.	0.151138716	0.001904762	483	525	0.49	0	0.594056365	0.38788749	Subclonal	passenger	D	N	T	Passenger	TRUE	FALSE	TRUE
CHK12	23	53223866	G	A	KDM5C	p.R1165C	Missense_Mutation	FALSE	.	0.162751678	0.001919386	596	521	0.52	0	0.621925969	0.430945833	Subclonal	likely_pathogenic	D	D	D	Passenger	TRUE	TRUE	TRUE
CHK12	12	18435596	G	C	PIK3C2G	p.R194T	Missense_Mutation	FALSE	.	0.170542636	0	258	226	0.55	0	0.707950415	0.412503698	Subclonal	passenger	N	N	T	Passenger	FALSE	FALSE	FALSE
CHK12	10	8111502	A	AG	GATA3	p.N331Efs*21	Frame_Shift_Ins	FALSE	.	0.286725664	0	565	435	1	0.6823	1	0.922830111	Clonal	likely_pathogenic	D	.	.	.	TRUE	TRUE	TRUE
CHK12	16	89355041	G	C	ANKRD11	p.Y213*	Nonsense_Mutation	FALSE	loh	0.342066958	0	687	743	1	0.8977	1	0.924171928	Clonal	likely_pathogenic	D	.	.	.	FALSE	FALSE	FALSE
CHK13	1	27105793	G	T	ARID1A	p.E1802*	Nonsense_Mutation	FALSE	.	0.176646707	0	334	292	1	0.85502	1	0.76682308	Clonal	likely_pathogenic	D	.	.	.	TRUE	TRUE	TRUE
CHK13	4	55144110	A	G	PDGFRA	p.I647V	Missense_Mutation	FALSE	.	0.183727034	0	381	302	1	0.8872	1	0.803374078	Clonal	likely_pathogenic	D	N	D	Driver	TRUE	TRUE	FALSE
CHK13	8	55370856	G	C	SOX17	p.G53A	Missense_Mutation	FALSE	.	0.283842795	0	229	185	1	0.96941	1	0.910863741	Clonal	likely_pathogenic	D	N	D	Passenger	FALSE	TRUE	TRUE
CHK2	23	123185226	TACTC	T	STAG2	p.T395Lfs*29	Frame_Shift_Del	FALSE	loh	0.153846154	0	468	346	0.72	0.02006	0.881104903	0.575583854	Subclonal	likely_pathogenic	.	.	.	.	TRUE	TRUE	TRUE
CHK2	3	178936091	G	A	PIK3CA	p.E545K	Missense_Mutation	TRUE	loh	0.319727891	0	294	242	1	0.05925	1	0.920090317	Clonal	likely_pathogenic	D	D	T	Driver	TRUE	TRUE	TRUE
CHK2	6	26031925	G	A	HIST1H3B	p.P122S	Missense_Mutation	FALSE	.	0.341789052	0	749	562	1	0.98471	1	0.974306488	Clonal	passenger	D	.	T	Passenger	TRUE	FALSE	TRUE
CHK2	17	7579521	C	A	TP53	p.E56*	Nonsense_Mutation	FALSE	loh	0.456221198	0	434	295	1	0.96987	1	0.971853707	Clonal	likely_pathogenic	A	.	.	.	TRUE	TRUE	TRUE
CHK2	16	68863561	T	TC	CDH1	p.D768*	Frame_Shift_Ins	FALSE	.	0.524017467	0	687	318	1	0.71018	1	0.984355566	Clonal	likely_pathogenic	.	.	.	.	TRUE	TRUE	TRUE
CHK3	7	151927066	C	T	KMT2C	p.R973K	Missense_Mutation	FALSE	loh	0.03649635	0	274	722	0.09	0	0.164251879	0.045961004	Subclonal	likely_pathogenic	D	N	D	Passenger	TRUE	TRUE	TRUE
CHK3	12	25398284	C	A	KRAS	p.G12V	Missense_Mutation	TRUE	.	0.144486692	0.001745201	263	573	0.51	0	0.679846873	0.376905365	Subclonal	likely_pathogenic	D	D	T	Driver	TRUE	TRUE	TRUE
CHK3	1	36932020	G	C	CSF3R	p.L817V	Missense_Mutation	FALSE	loh	0.188679245	0	265	558	0.48	0	0.610849061	0.370042376	Subclonal	passenger	N	N	T	Passenger	TRUE	FALSE	FALSE
CHK3	3	178952085	A	G	PIK3CA	p.H1047R	Missense_Mutation	TRUE	.	0.383529412	0	425	440	1	0.09827	1	0.968527945	Clonal	likely_pathogenic	D	N	T	Driver	TRUE	TRUE	TRUE
CHK3	16	23641767	CT	C	PALB2	p.E570Rfs*29	Frame_Shift_Del	FALSE	.	0.432258065	0	155	460	1	0.90277	1	0.841195974	Clonal	likely_pathogenic	.	.	.	.	TRUE	FALSE	FALSE
CHK3	4	187627932	T	C	FAT1	p.Y1017C	Missense_Mutation	FALSE	loh	0.472727273	0	275	651	1	0.75892	1	0.922348358	Clonal	passenger	D	D	T	Passenger	TRUE	FALSE	TRUE
CHK4	7	2956952	G	T	CARD11	p.A892E	Missense_Mutation	FALSE	.	0.133676093	0.003367003	389	297	0.88	0.63415	1	0.670472582	Clonal	passenger	D	N	T	Passenger	TRUE	FALSE	TRUE
CHK4	1	16260749	G	A	SPEN	p.V2672M	Missense_Mutation	FALSE	.	0.193661972	0	284	342	0.89	0.65329	1	0.686612955	Clonal	passenger	D	N	T	Passenger	TRUE	FALSE	TRUE
CHK4	10	8115745	G	C	GATA3	p.R364T	Missense_Mutation	FALSE	loh	0.22815534	0	412	487	1	0.89306	1	0.830138022	Clonal	likely_pathogenic	D	D	D	Passenger	TRUE	TRUE	TRUE
CHK4	6	152419926	A	G	ESR1	p.D538G	Missense_Mutation	FALSE	loh	0.24137931	0.012121212	232	330	1	0.88214	1	0.801160439	Clonal	passenger	D	D	T	Passenger	TRUE	FALSE	FALSE
CHK5	19	33793007	TCGC	T	CEBPA	p.G104del	In_Frame_Del	FALSE	.	0.054054054	0	74	29	0.13	0	0.313563373	0.048380751	Subclonal	passenger	N	N	.	.	TRUE	TRUE	TRUE
CHK5	21	36252884	C	CA	RUNX1	p.D160*	Frame_Shift_Ins	FALSE	.	0.435514019	0	535	167	1	0.94905	1	0.916256211	Clonal	likely_pathogenic	D	.	.	.	TRUE	TRUE	TRUE
CHK6	5	56167801	C	CT	MAP3K1	p.T457Yfs*4	Frame_Shift_Ins	FALSE	.	0.129485179	0	641	338	0.9	0.7072	1	0.723284672	Clonal	likely_pathogenic	.	.	.	.	FALSE	TRUE	TRUE
CHK6	5	56161654	A	C	MAP3K1	p.X385_splice	Splice_Site	FALSE	.	0.151696607	0	501	313	1	0.89587	1	0.803808502	Clonal	likely_pathogenic	D	.	.	.	FALSE	TRUE	TRUE
CHK6	3	178952085	A	G	PIK3CA	p.H1047R	Missense_Mutation	TRUE	.	0.157407407	0	540	342	1	0.91474	1	0.831106276	Clonal	likely_pathogenic	D	N	T	Driver	TRUE	TRUE	TRUE
CHK6	16	67650719	C	T	CTCF	p.R342C	Missense_Mutation	FALSE	.	0.279710145	0	690	503	1	0.9819	1	0.966429723	Clonal	passenger	D	D	T	Passenger	TRUE	TRUE	TRUE
CHK7	3	178921553	T	A	PIK3CA	p.N345K	Missense_Mutation	TRUE	.	0.212355212	0	259	310	0.6	0.00001	0.746193552	0.465767357	Subclonal	likely_pathogenic	D	D	T	Driver	TRUE	TRUE	TRUE
CHK7	16	67100699	C	T	CBFB	p.Q133*	Nonsense_Mutation	FALSE	.	0.357594937	0	316	463	0.65	0	0.745211035	0.553137752	Subclonal	likely_pathogenic	A	.	.	.	TRUE	TRUE	TRUE
CHK8	3	178936082	G	A	PIK3CA	p.E542K	Missense_Mutation	TRUE	.	0.027075812	0.001322751	554	756	0.26	0	0.414751101	0.152128547	Subclonal	likely_pathogenic	D	N	T	Driver	TRUE	TRUE	TRUE
CHK8	3	178938934	G	A	PIK3CA	p.E726K	Missense_Mutation	TRUE	.	0.027607362	0.002710027	652	738	0.26	0	0.405561497	0.162014913	Subclonal	likely_pathogenic	D	N	T	Driver	TRUE	TRUE	TRUE
CHK8	17	63533857	C	T	AXIN2	p.E433K	Missense_Mutation	FALSE	.	0.040540541	0	444	610	0.38	0.00001	0.595022018	0.240912552	Subclonal	passenger	D	D	T	Passenger	TRUE	TRUE	TRUE
CHK8	16	72832320	G	A	ZFHX3	p.Q1421*	Nonsense_Mutation	FALSE	loh	0.047169811	0	424	625	0.45	0.00013	0.676344414	0.287762228	Subclonal	likely_pathogenic	A	.	.	.	TRUE	FALSE	TRUE
CHK9	10	8115974	CG	C	GATA3	p.A441Pfs*34	Frame_Shift_Del	FALSE	.	0.270769231	0	325	116	0.94	0.80276	1	0.769955058	Clonal	likely_pathogenic	.	.	.	.	TRUE	TRUE	TRUE
CHK9	1	17355104	A	T	SDHB	p.D138E	Missense_Mutation	FALSE	.	0.296747967	0.002695418	738	371	1	0.94096	1	0.891148483	Clonal	likely_pathogenic	D	D	D	Passenger	TRUE	FALSE	FALSE

Whole-exome sequencing (TCGA)

Sample ID	Chromosome	Position	Reference Allele	Alternate Allele	Gene	Amino Acid Change	Mutation Type	Hotspot	LOH	Tumor MAF	Normal MAF	Tumor Depth (X)	Normal Depth (X)	Cancer Cell Fraction	Probability Mutation is Clonal	95% CI High	95% CI Low	Clonal/ Subclonal	Pathogenicity	Mutation Taster	PROVEAN	FATHMM	CHASM	Cancer Gene Census	Kandoth et. al.	Lawrence et. al.
TCGA-D8-A1XZ	15	50152542	G	A	ATP8B4	p.T1143I	Missense_Mutation	FALSE	loh	0.026109661	0	766	158	0.51	0.00687	0.779755338	0.329656971	Subclonal	passenger	D	D	T	Passenger	FALSE	FALSE	FALSE
TCGA-A8-A08J	3	96945239	G	A	EPHA6	p.D416N	Missense_Mutation	FALSE	.	0.026258206	0.002272727	457	440	0.15	0	0.255190331	0.081916829	Subclonal	likely_pathogenic	D	D	D	Passenger	FALSE	FALSE	FALSE
TCGA-E2-A15R	23	83723787	G	A	HDX	p.S315L	Missense_Mutation	FALSE	.	0.032608696	0	184	189	0.08	0	0.159075516	0.031211085	Subclonal	passenger	N	D	T	Passenger	FALSE	FALSE	FALSE
TCGA-BH-A1FM	3	32031152	G	T	ZNF860	p.R194I	Missense_Mutation	FALSE	.	0.034482759	0	203	99	0.2	0	0.393866006	0.092872178	Subclonal	passenger	D	D	T	Passenger	FALSE	FALSE	FALSE
TCGA-E2-A15R	23	73811648	G	A	RLIM	p.S501L	Missense_Mutation	FALSE	.	0.035714286	0.007142857	140	140	0.08	0	0.186699895	0.032034715	Subclonal	passenger	D	N	T	Passenger	FALSE	FALSE	FALSE
TCGA-E9-A54Y	8	38205328	G	A	WHSC1L1	p.P121L	Missense_Mutation	FALSE	.	0.03902439	0	205	119	0.39	0.01354	0.744495562	0.196929338	Subclonal	passenger	D	N	T	Passenger	TRUE	FALSE	FALSE
TCGA-BH-A1FM	1	220287760	G	A	IARS2	p.W528*	Nonsense_Mutation	FALSE	.	0.040983607	0	244	152	0.32	0.00007	0.566438785	0.170260173	Subclonal	passenger	A	.	.	.	FALSE	FALSE	FALSE
TCGA-E2-A155	8	27147674	G	A	TRIM35	p.R262*	Nonsense_Mutation	FALSE	.	0.040983607	0	610	617	0.1	0	0.143727784	0.063395684	Subclonal	passenger	D	.	.	.	FALSE	FALSE	FALSE
TCGA-E9-A1RB	17	38445779	C	T	CDC6	p.P36L	Missense_Mutation	FALSE	loh	0.043956044	0	91	83	0.18	0.00001	0.440798196	0.069088926	Subclonal	passenger	N	N	T	Passenger	FALSE	FALSE	FALSE
TCGA-E2-A15R	5	158603839	A	G	RNF145	p.M171T	Missense_Mutation	FALSE	.	0.044444444	0	90	91	0.11	0	0.25272269	0.037558091	Subclonal	likely_pathogenic	D	D	T	Driver	FALSE	FALSE	FALSE
TCGA-BH-A1FM	3	142131391	C	T	XRN1	p.D570N	Missense_Mutation	FALSE	.	0.045112782	0.008928571	133	112	0.26	0.00014	0.540074226	0.116931705	Subclonal	passenger	D	D	T	Passenger	FALSE	FALSE	FALSE
TCGA-D8-A1XZ	1	118166421	C	T	FAM46C	p.R311C	Missense_Mutation	FALSE	.	0.045454545	0	88	120	0.17	0	0.400791442	0.06236377	Subclonal	passenger	D	D	T	Passenger	TRUE	FALSE	TRUE
TCGA-D8-A1XZ	4	122085228	G	A	TNIP3	p.T95M	Missense_Mutation	FALSE	.	0.045454545	0	110	106	0.17	0	0.367659931	0.068291577	Subclonal	passenger	N	N	T	Passenger	FALSE	FALSE	FALSE
TCGA-E2-A155	6	107566769	C	T	PDSS2	p.E229K	Missense_Mutation	FALSE	loh	0.046822742	0	897	117	0.54	0.00009	0.714771689	0.394956366	Subclonal	passenger	D	N	T	Passenger	FALSE	FALSE	TRUE
TCGA-E9-A1RI	9	27455217	G	A	MOB3B	p.A111V	Missense_Mutation	FALSE	.	0.048192771	0	83	53	0.21	0.00011	0.517087292	0.082031554	Subclonal	passenger	D	N	.	Passenger	FALSE	FALSE	FALSE
TCGA-BH-A1FM	15	50152594	T	C	ATP8B4	p.R1126G	Missense_Mutation	FALSE	.	0.048484848	0	165	89	0.28	0.00003	0.527790203	0.139105646	Subclonal	passenger	D	D	T	Passenger	FALSE	FALSE	FALSE
TCGA-D8-A1XZ	5	178540927	G	C	ADAMTS2	p.Q1193E	Missense_Mutation	FALSE	.	0.048611111	0.003205128	288	312	0.27	0	0.447051485	0.160842641	Subclonal	passenger	D	N	T	Passenger	FALSE	FALSE	FALSE
TCGA-E9-A54Y	8	38205349	G	A	WHSC1L1	p.S114L	Missense_Mutation	FALSE	.	0.048648649	0	185	103	0.49	0.08811	0.868756529	0.255818628	Subclonal	passenger	N	N	T	Passenger	TRUE	FALSE	FALSE
TCGA-D8-A1XY	3	170141061	G	A	CLDN11	p.G113S	Missense_Mutation	FALSE	.	0.05	0	120	88	0.38	0.03039	0.776223678	0.172341194	Subclonal	likely_pathogenic	D	N	D	Passenger	FALSE	FALSE	FALSE
TCGA-D8-A1XZ	2	191899324	C	G	STAT4	p.X524_splice	Splice_Site	FALSE	.	0.050420168	0	119	131	0.18	0	0.380935271	0.081716466	Subclonal	passenger	D	.	.	.	FALSE	FALSE	FALSE
TCGA-A2-A3KD	1	25558623	C	T	SYF2	p.R35H	Missense_Mutation	FALSE	.	0.051282051	0	117	118	0.22	0	0.452890967	0.097962388	Subclonal	passenger	D	N	T	Passenger	FALSE	FALSE	FALSE
TCGA-D8-A1XY	17	29220855	C	T	ATAD5	p.L1662F	Missense_Mutation	FALSE	.	0.051813472	0	193	98	0.44	0.02283	0.782166792	0.23929283	Subclonal	passenger	N	N	T	Passenger	FALSE	FALSE	FALSE
TCGA-E2-A15H	2	111416077	G	C	BUB1	p.Q476E	Missense_Mutation	FALSE	.	0.054404145	0	386	340	0.25	0	0.369225523	0.159776157	Subclonal	passenger	D	N	T	Passenger	FALSE	FALSE	FALSE
TCGA-D8-A1XY	12	29639257	G	A	OVCH1	p.S306L	Missense_Mutation	FALSE	.	0.054945055	0	91	90	0.36	0.03657	0.782475557	0.153501329	Subclonal	passenger	N	N	D	Passenger	FALSE	FALSE	FALSE
TCGA-D8-A1JN	5	158603804	T	C	RNF145	p.M183V	Missense_Mutation	FALSE	.	0.056179775	0	89	52	0.13	0	0.285910899	0.052573971	Subclonal	passenger	D	N	T	Passenger	FALSE	FALSE	FALSE
TCGA-D8-A1XZ	9	140901231	G	C	CACNA1B	p.E663Q	Missense_Mutation	FALSE	.	0.056179775	0	89	124	0.21	0	0.451268366	0.085541136	Subclonal	likely_pathogenic	D	D	D	Passenger	FALSE	FALSE	FALSE
TCGA-E9-A54Y	8	124792286	C	T	FAM91A1	p.S204F	Missense_Mutation	FALSE	.	0.056451613	0	124	56	0.85	0.64904	0.986892833	0.377528974	Clonal	passenger	D	N	T	Passenger	FALSE	FALSE	FALSE
TCGA-E9-A1RI	11	101362353	C	A	TRPC6	p.Q354H	Missense_Mutation	FALSE	.	0.060606061	0	66	57	0.27	0.00264	0.642487828	0.10447541	Subclonal	passenger	N	N	T	Passenger	FALSE	FALSE	FALSE
TCGA-BH-A1FM	3	132175425	G	A	DNAJC13	p.X393_splice	Splice_Site	FALSE	.	0.061403509	0	228	152	0.35	0.00001	0.572008898	0.208129504	Subclonal	passenger	D	.	.	.	FALSE	FALSE	FALSE
TCGA-D8-A1XC	14	67555762	G	T	GPHN	p.A403S	Missense_Mutation	FALSE	.	0.0625	0	48	66	0.15	0	0.399971114	0.049567884	Subclonal	likely_pathogenic	D	D	.	Driver	TRUE	FALSE	FALSE
TCGA-D8-A1XY	17	9586182	G	A	USP43	p.R383H	Missense_Mutation	FALSE	loh	0.0625	0	128	154	0.28	0.00003	0.532095372	0.141688383	Subclonal	passenger	N	N	T	Passenger	FALSE	FALSE	FALSE
TCGA-E9-A54Y	8	145223294	C	T	MROH1	p.P40L	Missense_Mutation	FALSE	.	0.0625	0	96	65	0.94	0.68801	0.988781969	0.375399823	Clonal	passenger	D	D	T	Passenger	FALSE	FALSE	FALSE
TCGA-D8-A1XZ	12	40716258	G	C	LRRK2	p.G1819R	Missense_Mutation	FALSE	.	0.063063063	0	111	175	0.23	0	0.449863938	0.109550316	Subclonal	passenger	D	D	T	Passenger	FALSE	TRUE	FALSE
TCGA-A7-A4SC	4	9784882	A	G	DRD5	p.Y410C	Missense_Mutation	FALSE	.	0.064516129	0	62	61	0.29	0.00791	0.693859993	0.113961479	Subclonal	passenger	D	N	T	Passenger	FALSE	FALSE	FALSE
TCGA-E2-A155	4	87746702	C	G	SLC10A6	p.A264P	Missense_Mutation	FALSE	.	0.064516129	0	155	185	0.16	0	0.279212124	0.083135774	Subclonal	passenger	D	D	T	Passenger	FALSE	FALSE	FALSE
TCGA-A2-A3KD	20	39977426	G	T	LPIN3	p.Q152H	Missense_Mutation	FALSE	.	0.065217391	0	46	47	0.28	0.01585	0.734696047	0.09571588	Subclonal	passenger	N	N	T	Passenger	FALSE	FALSE	FALSE
TCGA-D8-A1XY	17	60814414	C	T	10-Mar	p.R272Q	Missense_Mutation	FALSE	.	0.06557377	0	61	59	0.56	0.42442	0.964650946	0.214410946	Subclonal	passenger	N	N	T	Passenger	FALSE	FALSE	FALSE
TCGA-BH-A1FM	17	65184443	G	T	HELZ	p.S385Y	Missense_Mutation	FALSE	.	0.065693431	0	137	42	1	0.78133	1	0.487207788	Clonal	passenger	D	D	T	Passenger	FALSE	FALSE	FALSE
TCGA-A8-A08J	12	96389631	C	T	HAL	p.A20T	Missense_Mutation	FALSE	.	0.06779661	0	59	43	0.52	0.2904	0.956712893	0.202882954	Subclonal	passenger	N	N	T	Passenger	FALSE	FALSE	FALSE
TCGA-D8-A1JN	19	38934267	C	T	RYR1	p.R114C	Missense_Mutation	FALSE	loh	0.068181818	0	44	25	0.16	0	0.420157441	0.052436843	Subclonal	likely_pathogenic	D	N	D	Passenger	FALSE	FALSE	FALSE
TCGA-D8-A1JN	3	165548178	C	T	BCHE	p.G215E	Missense_Mutation	FALSE	.	0.069444444	0	72	47	0.16	0	0.350232112	0.06630458	Subclonal	likely_pathogenic	D	D	D	Passenger	FALSE	FALSE	FALSE
TCGA-A8-A08J	2	152980416	T	C	STAM2	p.M405V	Missense_Mutation	FALSE	loh	0.071428571	0	126	137	0.34	0.00025	0.611773829	0.176673302	Subclonal	passenger	D	N	T	Passenger	FALSE	FALSE	FALSE
TCGA-D8-A1JN	18	24081087	C	A	KCTD1	p.G38V	Missense_Mutation	FALSE	loh	0.071428571	0.012195122	112	82	0.09	0	0.174223565	0.043574625	Subclonal	likely_pathogenic	D	D	D	Passenger	FALSE	FALSE	FALSE
TCGA-D8-A1XZ	19	14164464	C	T	PALM3	p.A659T	Missense_Mutation	FALSE	.	0.071428571	0	42	65	0.33	0.06018	0.839554824	0.115152275	Subclonal	passenger	N	N	T	Passenger	FALSE	FALSE	FALSE
TCGA-E9-A54Y	8	38205427	G	A	WHSC1L1	p.S88L	Missense_Mutation	FALSE	.	0.072072072	0	111	89	0.72	0.54437	0.97952743	0.355336721	Clonal	passenger	N	N	T	Passenger	TRUE	FALSE	FALSE
TCGA-D8-A1XY	8	42294888	C	T	SLC20A2	p.R381H	Missense_Mutation	FALSE	.	0.072289157	0	83	47	0.54	0.3168	0.947594641	0.249086976	Subclonal	likely_pathogenic	D	N	D	Passenger	FALSE	FALSE	FALSE
TCGA-A7-A4SC	4	9784853	C	G	DRD5	p.I400M	Missense_Mutation	FALSE	.	0.072463768	0	69	68	0.33	0.00875	0.712968491	0.140607085	Subclonal	passenger	N	N	T	Passenger	FALSE	FALSE	FALSE
TCGA-D8-A1XY	17	39197549	G	C	KRTAP1-1	p.S34C	Missense_Mutation	FALSE	.	0.074074074	0	54	67	0.63	0.51084	0.974360883	0.237022561	Clonal	passenger	N	N	T	Passenger	FALSE	FALSE	FALSE
TCGA-D8-A1XC	1	155160308	G	T	MUC1	p.T324N	Missense_Mutation	FALSE	.	0.075	0	40	19	0.26	0.00575	0.670620189	0.087488019	Subclonal	passenger	N	D	T	Passenger	FALSE	FALSE	FALSE
TCGA-E9-A1QZ	11	92534071	C	A	FAT3	p.A2631E	Missense_Mutation	FALSE	loh	0.076923077	0	39	28	0.58	0.54643	0.971296997	0.191835439	Clonal	passenger	N	N	T	Passenger	FALSE	FALSE	FALSE
TCGA-E9-A1R0	17	47783568	G	T	SLC35B1	p.Q113K	Missense_Mutation	FALSE	.	0.076923077	0	39	45	0.63	0.6035	0.975734147	0.203648402	Clonal	passenger	D	D	T	Passenger	FALSE	FALSE	FALSE
TCGA-AR-A0TW	17	25639270	C	G	WSB1	p.P381A	Missense_Mutation	FALSE	loh	0.077777778	0	90	34	0.4	0.02864	0.778140029	0.195632617	Subclonal	passenger	D	D	T	Passenger	FALSE	FALSE	FALSE
TCGA-D8-A1XZ	20	34505578	G	T	PHF20	p.M666I	Missense_Mutation	FALSE	.	0.078125	0	64	73	0.36	0.02279	0.778945959	0.155911955	Subclonal	passenger	D	N	T	Passenger	FALSE	FALSE	FALSE
TCGA-AR-A0TW	12	65564409	G	T	LEMD3	p.G345W	Missense_Mutation	FALSE	.	0.078947368	0	38	15	0.49	0.38291	0.955263855	0.166376948	Subclonal	passenger	D	N	T	Passenger	FALSE	FALSE	FALSE
TCGA-E9-A1RB	10	76748828	C	G	KAT6B	p.Q863E	Missense_Mutation	FALSE	.	0.079545455	0	88	63	0.57	0.32321	0.949559331	0.274759561	Subclonal	passenger	D	D	T	Passenger	TRUE	FALSE	FALSE
TCGA-D8-A1JN	13	25058867	C	T	PARP4	p.V458I	Missense_Mutation	FALSE	.	0.079646018	0	113	59	0.19	0	0.331572076	0.094419923	Subclonal	passenger	N	N	T	Passenger	FALSE	FALSE	FALSE
TCGA-E9-A1RB	16	2105460	T	C	TSC2	p.L180P	Missense_Mutation	FALSE	.	0.080882353	0	136	88	0.66	0.41512	0.964852442	0.36588384	Subclonal	passenger	D	D	T	Passenger	TRUE	FALSE	FALSE
TCGA-BH-A1FM	4	113461095	C	G	C4orf21	p.R2032S	Missense_Mutation	FALSE	.	0.081632653	0	147	109	0.47	0.01753	0.781481938	0.268324667	Subclonal	likely_pathogenic	D	D	D	Passenger	FALSE	FALSE	FALSE
TCGA-A8-A08J	12	113424885	G	A	OAS2	p.D74N	Missense_Mutation	FALSE	.	0.082882883	0	555	322	0.72	0.13001	0.931362714	0.542857184	Subclonal	passenger	N	D	T	Passenger	FALSE	FALSE	FALSE
TCGA-AR-A24O	4	169812089	G	T	PALLD	p.A661S	Missense_Mutation	FALSE	loh	0.083333333	0	36	45	0.67	0.58377	0.978017524	0.211896539	Clonal	passenger	D	N	T	Passenger	FALSE	FALSE	FALSE
TCGA-E9-A1NI	5	24488080	G	A	CDH10	p.R687W	Missense_Mutation	FALSE	.	0.083333333	0	36	36	0.45	0.16131	0.942860516	0.155365439	Subclonal	passenger	D	D	T	Passenger	FALSE	FALSE	FALSE
TCGA-A8-A08J	8	56652702	G	A	TMEM68	p.R255C	Missense_Mutation	FALSE	.	0.084158416	0	202	131	0.74	0.4187	0.975147733	0.455983739	Subclonal	passenger	D	D	.	Passenger	FALSE	FALSE	FALSE
TCGA-E9-A54Y	8	38205127	G	C	WHSC1L1	p.S188*	Nonsense_Mutation	FALSE	.	0.084291188	0	261	147	0.84	0.67301	0.987958812	0.546798066	Clonal	likely_pathogenic	A	.	.	.	TRUE	FALSE	FALSE
TCGA-AR-A0TW	20	4778716	CAAGAG	C	RASSF2	p.L24Afs*10	Frame_Shift_Del	FALSE	.	0.084848485	0	165	126	0.36	0.00001	0.570480977	0.210502952	Subclonal	passenger	D	.	.	.	FALSE	FALSE	FALSE
TCGA-E9-A1NI	1	75037073	T	A	C1orf173	p.T1441S	Missense_Mutation	FALSE	.	0.085106383	0	94	57	0.38	0.00214	0.698547905	0.190697707	Subclonal	passenger	N	N	T	Passenger	FALSE	FALSE	FALSE
TCGA-AR-A24O	12	105605050	T	C	APPL2	p.M111V	Missense_Mutation	FALSE	.	0.085365854	0	82	67	0.77	0.6428	0.98329504	0.354887582	Clonal	passenger	D	N	T	Passenger	FALSE	FALSE	FALSE
TCGA-D8-A1XY	20	46311847	C	T	SULF2	p.G319S	Missense_Mutation	FALSE	.	0.085365854	0	82	67	0.64	0.4662	0.969873878	0.307501075	Subclonal	likely_pathogenic	D	D	D	Passenger	FALSE	FALSE	FALSE
TCGA-A8-A08J	20	29891032	T	G	DEFB116	p.S98R	Missense_Mutation	FALSE	.	0.087046632	0	965	781	0.76	0.07375	0.921663743	0.613890821	Subclonal	passenger	N	N	T	Passenger	FALSE	FALSE	FALSE
TCGA-A2-A0T6	8	68062160	T	A	CSPP1	p.N701K	Missense_Mutation	FALSE	.	0.088	0	125	44	0.35	0.00004	0.590229455	0.192896945	Subclonal	passenger	D	N	T	Passenger	FALSE	FALSE	FALSE
TCGA-A7-A4SC	18	55282949	A	T	NARS	p.L89*	Nonsense_Mutation	FALSE	.	0.088607595	0	79	67	0.4	0.01697	0.770424126	0.195107974	Subclonal	passenger	D	.	.	.	FALSE	FALSE	FALSE
TCGA-BH-A1FM	7	94058543	C	A	COL1A2	p.T1252N	Missense_Mutation	FALSE	.	0.088607595	0	79	76	0.51	0.18575	0.91329429	0.247581666	Subclonal	passenger	D	N	T	Passenger	FALSE	FALSE	FALSE
TCGA-E9-A1RB	19	17740110	T	C	UNC13A	p.Q1231R	Missense_Mutation	FALSE	.	0.089285714	0	56	54	0.46	0.17811	0.906630274	0.197641763	Subclonal	passenger	N	N	T	Passenger	FALSE	FALSE	FALSE
TCGA-BH-A1FM	23	54280410	C	G	WNK3	p.E783D	Missense_Mutation	FALSE	.	0.089473684	0	190	113	0.51	0.01392	0.790079652	0.321269851	Subclonal	passenger	N	N	T	Passenger	FALSE	FALSE	FALSE
TCGA-A2-A3KD	11	6424398	G	A	APBB1	p.R361W	Missense_Mutation	FALSE	.	0.089552239	0	67	58	0.47	0.10491	0.896355281	0.217288683	Subclonal	passenger	N	N	T	Passenger	FALSE	FALSE	FALSE
TCGA-D8-A1XY	11	92714892	C	A	MTNR1B	p.T168N	Missense_Mutation	FALSE	.	0.090225564	0	133	117	0.59	0.2144	0.926528186	0.33791937	Subclonal	passenger	D	D	T	Passenger	FALSE	FALSE	FALSE
TCGA-AR-A24O	7	48312670	G	A	ABCA13	p.S1136N	Missense_Mutation	FALSE	.	0.090909091	0	55	50	0.82	0.67327	0.985157724	0.318926345	Clonal	passenger	N	N	D	Passenger	FALSE	FALSE	FALSE
TCGA-E2-A15R	16	28507445	G	C	APOBR	p.E361D	Missense_Mutation	FALSE	.	0.090909091	0	55	46	0.31	0.00232	0.655532	0.130922285	Subclonal	passenger	N	N	T	Passenger	FALSE	FALSE	FALSE
TCGA-E9-A1QZ	3	52469904	C	A	SEMA3G	p.K688N	Missense_Mutation	FALSE	.	0.090909091	0	33	35	0.69	0.62596	0.979103758	0.216586637	Clonal	passenger	N	N	T	Passenger	FALSE	FALSE	FALSE
TCGA-D8-A1XY	7	91570457	C	T	AKAP9	p.A15V	Missense_Mutation	FALSE	.	0.091666667	0	120	113	0.6	0.2655	0.938922891	0.334882882	Subclonal	passenger	D	N	T	Passenger	FALSE	FALSE	FALSE
TCGA-A2-A3KD	14	74988688	C	T	LTBP2	p.R905H	Missense_Mutation	FALSE	.	0.092307692	0	65	44	0.49	0.12605	0.909032648	0.223937721	Subclonal	passenger	N	N	T	Passenger	FALSE	FALSE	FALSE

TCGA-E9-A1NI	10	75560463	T	TC	ZSWIM8	p.Y1701Lfs*3	Frame_Shift_Ins	FALSE	.	0.092592593	0	54	73	0.41	0.03886	0.848560554	0.176357856	Subclonal	passenger	D	.	.	.	FALSE	FALSE	FALSE
TCGA-A2-A0T6	2	9599739	T	A	CPSF3	p.I593K	Missense_Mutation	FALSE	.	0.093023256	0	129	145	0.37	0.00007	0.609410925	0.209449032	Subclonal	passenger	D	N	T	Passenger	FALSE	FALSE	FALSE
TCGA-E9-A1R0	1	39920671	G	T	MACF1	p.A4934S	Missense_Mutation	FALSE	.	0.093023256	0	43	88	0.76	0.68318	0.982697634	0.272174332	Clonal	passenger	D	N	T	Passenger	FALSE	FALSE	FALSE
TCGA-A7-A4SC	17	78449433	A	C	NPTX1	p.V177G	Missense_Mutation	FALSE	.	0.09375	0.010989011	64	91	0.43	0.05127	0.837549067	0.196031227	Subclonal	passenger	D	D	T	Passenger	FALSE	FALSE	FALSE
TCGA-D8-A1XZ	1	63920577	T	A	ITGB3BP	p.N145I	Missense_Mutation	FALSE	.	0.096385542	0	83	120	0.35	0.00083	0.648959667	0.178223537	Subclonal	passenger	N	D	T	Passenger	FALSE	FALSE	FALSE
TCGA-D8-A1XY	17	41346494	C	T	NBR1	p.S551F	Missense_Mutation	FALSE	.	0.096491228	0	114	64	0.82	0.66759	0.986533708	0.442512449	Clonal	passenger	D	D	T	Passenger	FALSE	FALSE	FALSE
TCGA-D8-A1XZ	13	42869865	CTGCTCATATACAGGTATGG	C	AKAP11	p.X69_splice	Splice_Site	FALSE	.	0.098765432	0.004484305	162	223	0.36	0	0.559375311	0.221215815	Subclonal	passenger	D	.	.	.	FALSE	FALSE	FALSE
TCGA-E9-A1QZ	23	130408064	G	A	IGSF1	p.R1295*	Nonsense_Mutation	FALSE	.	0.098901099	0	91	92	0.75	0.65168	0.981336383	0.383714666	Clonal	passenger	D	.	.	.	FALSE	FALSE	FALSE
TCGA-A2-A3KD	1	158724608	G	A	OR6K6	p.M1?	Translation_Start_Site	FALSE	.	0.1	0	70	31	0.63	0.33408	0.96611652	0.301456115	Subclonal	passenger	N	T	T	Passenger	FALSE	FALSE	FALSE
TCGA-D8-A1XY	11	47204094	C	T	PACSIN3	p.R24H	Missense_Mutation	FALSE	.	0.1	0	40	34	0.65	0.53178	0.976011304	0.244406309	Clonal	passenger	D	D	T	Passenger	FALSE	FALSE	FALSE
TCGA-E9-A1NI	22	18022285	T	TC	CECR2	p.N800Qfs*5	Frame_Shift_Ins	FALSE	loh	0.1	0	20	27	0.34	0.09212	0.903692168	0.098055038	Subclonal	likely_pathogenic	D	.	.	.	FALSE	FALSE	FALSE
TCGA-AR-A0TW	1	227235647	G	C	CDC42BPA	p.S990*	Nonsense_Mutation	FALSE	.	0.101886792	0	265	110	0.73	0.4089	0.963544561	0.504014259	Subclonal	passenger	A	.	.	.	FALSE	FALSE	FALSE
TCGA-D8-A1JN	14	33292059	C	G	AKAP6	p.I1680M	Missense_Mutation	FALSE	.	0.102040816	0	49	33	0.24	0.00002	0.502802531	0.100560055	Subclonal	passenger	D	N	T	Passenger	FALSE	FALSE	FALSE
TCGA-E9-A1R0	19	16040359	T	C	CYP4F11	p.Y84C	Missense_Mutation	FALSE	.	0.102040816	0	49	108	0.84	0.72368	0.985782529	0.32433292	Clonal	passenger	N	D	D	Passenger	FALSE	FALSE	FALSE
TCGA-E2-A15R	19	18376110	T	TG	KIAA1683	p.Q747Pfs*18	Frame_Shift_Ins	FALSE	.	0.102150538	0.035928144	186	167	0.24	0	0.359966811	0.152439928	Subclonal	passenger	.	.	.	.	FALSE	FALSE	FALSE
TCGA-A8-A08J	17	65105763	ATTCAG	A	HELZ	p.P1318Lfs*4	Frame_Shift_Del	FALSE	loh	0.102661597	0	789	231	1	0.96867	1	0.92265351	Clonal	likely_pathogenic	D	.	.	.	FALSE	FALSE	FALSE
TCGA-D8-A1XZ	8	69021753	T	A	PREX2	p.L1014Q	Missense_Mutation	FALSE	.	0.103174603	0	126	66	0.89	0.64717	0.989914825	0.495891222	Clonal	passenger	D	D	T	Passenger	TRUE	FALSE	FALSE
TCGA-BH-A1FM	4	184182548	G	T	WWC2	p.S591I	Missense_Mutation	FALSE	.	0.103448276	0	87	80	0.59	0.30395	0.946882952	0.313436125	Subclonal	passenger	D	N	T	Passenger	FALSE	FALSE	FALSE
TCGA-E9-A1R0	3	47032777	G	A	NBEAL2	p.V209I	Missense_Mutation	FALSE	.	0.103448276	0	87	183	0.85	0.74549	0.987501881	0.422202669	Clonal	passenger	D	N	T	Passenger	FALSE	FALSE	FALSE
TCGA-E9-A54Y	17	78115160	C	G	EIF4A3	p.L110F	Missense_Mutation	FALSE	.	0.103448276	0	29	21	0.93	0.66298	0.986121193	0.258145616	Clonal	passenger	D	D	T	Passenger	FALSE	FALSE	FALSE
TCGA-D8-A1JN	13	39438637	T	A	FREM2	p.F2626Y	Missense_Mutation	FALSE	.	0.103896104	0	77	64	0.24	0	0.442268204	0.120997823	Subclonal	passenger	D	N	T	Passenger	FALSE	FALSE	FALSE
TCGA-E9-A1QZ	12	110006636	G	C	MMAB	p.P77A	Missense_Mutation	FALSE	.	0.104477612	0	67	80	0.79	0.69335	0.984469149	0.363703668	Clonal	likely_pathogenic	D	D	D	Passenger	FALSE	FALSE	FALSE
TCGA-E9-A1NI	23	37312610	A	AC	PRRG1	p.P135Tfs*3	Frame_Shift_Ins	FALSE	.	0.106557377	0.02	122	100	0.58	0.07305	0.906536878	0.339652582	Subclonal	passenger	D	.	.	.	FALSE	FALSE	FALSE
TCGA-A2-A3KD	2	128477386	T	G	WDR33	p.Q738P	Missense_Mutation	FALSE	.	0.107142857	0	28	21	0.46	0.21966	0.941966915	0.157379297	Subclonal	likely_pathogenic	D	N	D	Passenger	FALSE	FALSE	FALSE
TCGA-AR-A24O	17	465894	G	T	VPS53	p.P469T	Missense_Mutation	FALSE	loh	0.107142857	0	28	30	0.75	0.63465	0.98178652	0.229841085	Clonal	likely_pathogenic	D	D	T	Driver	FALSE	FALSE	FALSE
TCGA-D8-A1XZ	2	138208561	C	T	THSD7B	p.R1036*	Nonsense_Mutation	FALSE	.	0.107142857	0	84	87	0.39	0.00214	0.694287949	0.206275163	Subclonal	passenger	D	.	.	.	FALSE	FALSE	FALSE
TCGA-AR-A0TW	23	7868810	T	A	PNPLA4	p.S227C	Missense_Mutation	FALSE	.	0.109004739	0	211	113	0.78	0.59373	0.98050396	0.520246015	Clonal	passenger	N	D	T	Passenger	FALSE	FALSE	FALSE
TCGA-BH-A1FM	14	91356949	G	A	RPS6KA5	p.P573L	Missense_Mutation	FALSE	loh	0.109090909	0	55	77	0.41	0.04112	0.804139659	0.18865123	Subclonal	passenger	D	D	T	Passenger	FALSE	FALSE	FALSE
TCGA-AR-A0TW	23	7868809	C	T	PNPLA4	p.S227N	Missense_Mutation	FALSE	.	0.110047847	0	209	113	0.79	0.61059	0.981753843	0.524587787	Clonal	passenger	N	N	T	Passenger	FALSE	FALSE	FALSE
TCGA-AR-A24O	3	195968901	A	C	PCYT1A	p.I209S	Missense_Mutation	FALSE	.	0.111111111	0	54	52	1	0.75696	0.989975702	0.393118317	Clonal	passenger	D	D	.	Passenger	FALSE	FALSE	FALSE
TCGA-E9-A1NI	11	133815978	C	CG	IGSF9B	p.H81Afs*5	Frame_Shift_Ins	FALSE	loh	0.111111111	0	18	59	0.38	0.13108	0.928656634	0.10840339	Subclonal	likely_pathogenic	D	.	.	.	FALSE	FALSE	FALSE
TCGA-E2-A15R	12	107126738	C	G	RFX4	p.A512G	Missense_Mutation	FALSE	.	0.112149533	0	107	191	0.27	0	0.435910184	0.150617552	Subclonal	passenger	N	N	T	Passenger	FALSE	FALSE	FALSE
TCGA-D8-A1XZ	20	34242060	C	T	RBM12	p.M395I	Missense_Mutation	FALSE	.	0.112244898	0	98	103	0.52	0.05563	0.864211479	0.293490032	Subclonal	passenger	D	N	T	Passenger	FALSE	FALSE	FALSE
TCGA-E2-A15R	2	233348784	G	T	ECEL1	p.A445D	Missense_Mutation	FALSE	.	0.112359551	0	89	84	0.27	0	0.456746978	0.143127715	Subclonal	passenger	D	N	T	Passenger	FALSE	FALSE	FALSE
TCGA-D8-A1XY	11	5529276	G	T	UBQLN3	p.L505M	Missense_Mutation	FALSE	.	0.114285714	0	35	19	0.75	0.60534	0.981921814	0.269642033	Clonal	passenger	N	N	T	Passenger	FALSE	FALSE	FALSE
TCGA-A8-A08J	2	122122750	G	A	CLASP1	p.R1333W	Missense_Mutation	FALSE	.	0.114754098	0	61	54	0.66	0.39658	0.971784791	0.315599321	Subclonal	passenger	D	D	T	Passenger	FALSE	FALSE	FALSE
TCGA-D8-A1XY	5	77511985	C	T	AP3B1	p.R227H	Missense_Mutation	FALSE	.	0.114754098	0	122	105	0.75	0.56212	0.979074964	0.443144357	Clonal	passenger	D	D	T	Passenger	FALSE	FALSE	FALSE
TCGA-AR-A24O	16	3363109	G	A	ZNF75A	p.D12N	Missense_Mutation	FALSE	.	0.114942529	0	87	75	1	0.79802	1	0.497200292	Clonal	passenger	N	.	.	Passenger	FALSE	FALSE	FALSE
TCGA-A2-A0T6	16	29912801	A	AGGG	ASPHD1	p.G172dup	In_Frame_Ins	FALSE	.	0.115384615	0	26	40	0.46	0.22402	0.940268692	0.157101289	Subclonal	passenger	.	.	.	.	FALSE	FALSE	FALSE
TCGA-E2-A15H	1	240969512	C	A	RGS7	p.L399F	Missense_Mutation	FALSE	.	0.115384615	0	572	350	0.87	0.7297	0.988755418	0.685520116	Clonal	passenger	D	N	T	Passenger	FALSE	FALSE	FALSE
TCGA-BH-A1FM	10	37508553	G	T	ANKRD30A	p.E1249*	Nonsense_Mutation	FALSE	.	0.11627907	0	86	76	0.55	0.16186	0.907876113	0.301847771	Subclonal	passenger	A	.	.	.	FALSE	FALSE	FALSE
TCGA-E9-A54Y	6	33173041	G	C	HSD17B8	p.G105A	Missense_Mutation	FALSE	.	0.117647059	0.019230769	68	52	0.83	0.64781	0.986327065	0.397175597	Clonal	passenger	D	D	T	Passenger	FALSE	FALSE	FALSE
TCGA-E9-A1QZ	2	192701286	G	T	SDPR	p.A214D	Missense_Mutation	FALSE	.	0.118421053	0.017857143	76	56	0.9	0.76452	0.989149444	0.439191326	Clonal	passenger	N	N	T	Passenger	FALSE	FALSE	FALSE
TCGA-E9-A1NI	23	107977802	G	GC	IRS4	p.K592Qfs*12	Frame_Shift_Ins	FALSE	.	0.119402985	0	67	99	0.77	0.41421	0.982990903	0.376380618	Subclonal	passenger	D	.	.	.	FALSE	FALSE	FALSE
TCGA-BH-A1FM	11	18524118	G	T	TSG101	p.Y185*	Nonsense_Mutation	FALSE	.	0.121212121	0	66	86	0.7	0.52447	0.976011204	0.34847531	Clonal	passenger	A	.	.	.	FALSE	FALSE	FALSE
TCGA-BH-A1FM	2	135711146	G	T	CCNT2	p.G374V	Missense_Mutation	FALSE	loh	0.121212121	0	33	58	0.45	0.20945	0.917484923	0.178952617	Subclonal	passenger	D	N	T	Passenger	FALSE	FALSE	FALSE
TCGA-AR-A24O	21	40601356	C	A	BRWD1	p.E1003*	Nonsense_Mutation	FALSE	.	0.121621622	0	74	68	1	0.80822	1	0.492135521	Clonal	passenger	A	.	.	.	FALSE	FALSE	FALSE
TCGA-A2-A0T6	7	130417900	T	G	KLF14	p.T321P	Missense_Mutation	FALSE	.	0.12195122	0.019607843	41	51	0.48	0.15231	0.917240963	0.20785295	Subclonal	passenger	N	.	T	Passenger	FALSE	FALSE	FALSE
TCGA-AR-A24O	14	38061463	T	C	FOXA1	p.I176V	Missense_Mutation	TRUE	.	0.122807018	0	57	46	1	0.79612	1	0.444706295	Clonal	likely_pathogenic	D	N	D	Passenger	TRUE	TRUE	TRUE
TCGA-E9-A1QZ	12	4708239	C	A	DYRK4	p.F202L	Missense_Mutation	FALSE	.	0.123287671	0	73	76	0.93	0.78112	1	0.450969314	Clonal	passenger	D	D	T	Passenger	FALSE	FALSE	FALSE
TCGA-E9-A1NI	20	37146232	A	AC	RALGAPB	p.H382Tfs*2	Frame_Shift_Ins	FALSE	.	0.123376623	0.012048193	154	83	1	0.68346	1	0.611911808	Clonal	passenger	D	.	.	.	FALSE	FALSE	FALSE
TCGA-A2-A0T6	16	81397481	C	A	GAN	p.S390Y	Missense_Mutation	FALSE	loh	0.123595506	0	178	316	0.36	0	0.525995198	0.240309056	Subclonal	passenger	D	.	T	Passenger	FALSE	FALSE	FALSE
TCGA-D8-A1XY	8	3256949	G	T	CSMD1	p.A790E	Missense_Mutation	FALSE	.	0.124087591	0	137	136	0.93	0.77308	1	0.556037764	Clonal	passenger	D	D	T	Passenger	FALSE	FALSE	FALSE
TCGA-E2-A15R	11	94759549	C	A	KDM4E	p.Y276*	Nonsense_Mutation	FALSE	loh	0.125	0.011363636	32	88	0.17	0	0.381071229	0.0646406	Subclonal	likely_pathogenic	D	.	.	.	FALSE	FALSE	FALSE
TCGA-E9-A1NI	20	37203484	T	TC	RALGAPB	p.H1456Pfs*43	Frame_Shift_Ins	FALSE	.	0.125	0	48	70	1	0.57476	1	0.40466401	Clonal	passenger	D	.	.	.	FALSE	FALSE	FALSE
TCGA-E9-A1QZ	17	27239625	G	A	PHF12	p.T655I	Missense_Mutation	FALSE	.	0.125	0	48	44	1	0.73926	1	0.407920411	Clonal	likely_pathogenic	D	N	D	Passenger	FALSE	FALSE	FALSE
TCGA-E9-A54Y	1	43912708	G	T	SZT2	p.R2995L	Missense_Mutation	FALSE	.	0.125	0	24	52	0.5	0.35403	0.955771303	0.17177543	Subclonal	passenger	D	D	.	Passenger	FALSE	FALSE	FALSE
TCGA-A8-A08J	13	46544025	C	T	ZC3H13	p.R885K	Missense_Mutation	FALSE	loh	0.12627551	0	784	693	0.72	0.00484	0.863202895	0.596122549	Subclonal	passenger	D	N	T	Passenger	FALSE	FALSE	FALSE
TCGA-A8-A08J	7	11675946	C	G	THSD7A	p.G278A	Missense_Mutation	FALSE	.	0.126506024	0	166	164	0.73	0.34483	0.967195511	0.476456201	Subclonal	passenger	N	N	T	Passenger	FALSE	FALSE	FALSE
TCGA-D8-A1XC	2	166012326	G	C	SCN3A	p.F373L	Missense_Mutation	FALSE	.	0.126984127	0	63	45	0.3	0.00001	0.551009968	0.154188802	Subclonal	likely_pathogenic	D	D	D	Passenger	FALSE	FALSE	FALSE
TCGA-D8-A1XZ	13	79945155	AACTTCG	A	RBM26	p.R185_S186del	In_Frame_Del	FALSE	.	0.126984127	0	126	190	0.46	0.00098	0.712836289	0.286910436	Subclonal	passenger	N	.	.	.	FALSE	FALSE	FALSE
TCGA-E9-A1NI	7	100205413	G	GC	PCOLCE	p.M392Hfs*81	Frame_Shift_Ins	FALSE	.	0.126984127	0.013513514	63	74	0.82	0.46084	0.985849563	0.394540401	Subclonal	passenger	D	.	.	.	FALSE	FALSE	FALSE
TCGA-D8-A1XY	3	182810273	C	T	MCCC1	p.R66H	Missense_Mutation	FALSE	.	0.12716763	0	173	115	0.96	0.80217	1	0.604597882	Clonal	likely_pathogenic	D	D	D	Passenger	FALSE	FALSE	FALSE
TCGA-AR-A0TW	8	26484767	G	A	DPYSL2	p.A193T	Missense_Mutation	FALSE	loh	0.129032258	0	62	53	0.41	0.01627	0.742453011	0.210056221	Subclonal	likely_pathogenic	D	D	D	Driver	FALSE	FALSE	FALSE
TCGA-BH-A1FM	22	32108301	A	G	PRR14L	p.S1842P	Missense_Mutation	FALSE	loh	0.131578947	0	38	58	0.49	0.22321	0.923555762	0.212859446	Subclonal	passenger	N	D	T	Passenger	FALSE	FALSE	FALSE
TCGA-AR-A24O	13	107145608	G	A	EFNB2	p.P261L	Missense_Mutation	FALSE	.	0.132075472	0	53	47	1	0.81644	1	0.460239894	Clonal	likely_pathogenic	D	D	D	Passenger	FALSE	FALSE	FALSE
TCGA-AR-A0TW	4	80328163	C	T	GK2	p.E398K	Missense_Mutation	FALSE	.	0.133333333	0	225	66	0.82	0.65932	0.985023198	0.576816707	Clonal	likely_pathogenic	D	D	D	Passenger	FALSE	FALSE	FALSE
TCGA-D8-A1XC	12	12630318	G	A	DUSP16	p.R483*	Nonsense_Mutation	TRUE	.	0.133333333	0	30	32	0.32	0.00662	0.709626622	0.125768675	Subclonal	passenger	N	.	.	.	FALSE	FALSE	FALSE
TCGA-E9-A1QZ	7	31735165	T	A	PPP1R17	p.N55K	Missense_Mutation	FALSE	.	0.133333333	0	75	64	1	0.79518	1	0.524052404	Clonal	passenger	N	D	T	Passenger	FALSE	FALSE	FALSE
TCGA-E9-A1RB	11	67050279	C	T	ADRBK1	p.T406M	Missense_Mutation	FALSE	.	0.133333333	0	30	28	0.82	0.64582	0.984682004	0.287241639	Clonal	likely_pathogenic	D	D	T	Driver	FALSE	FALSE	FALSE
TCGA-E9-A1R0	18	21894248	G	A	OSBPL1A	p.R312W	Missense_Mutation	FALSE	.	0.134615385	0	52	83	1	0.82565	1	0.445745715	Clonal	passenger	D	N	T	Passenger	FALSE	FALSE	FALSE
TCGA-BH-A1FM	15	59323545	G	A	RNF111	p.R175K	Missense_Mutation	FALSE	.	0.135135135	0	74	88	0.64	0.37739	0.959256199	0.350150654	Subclonal	passenger	D	N	T	Passenger	FALSE	FALSE	FALSE
TCGA-E2-A15R	9	134354682	G	A	PRRC2B	p.R1601Q	Missense_Mutation	FALSE	.	0.135869565	0	184	163	0.32	0	0.452311322	0.217245266	Subclonal	passenger	D	N	T	Passenger	FALSE	FALSE	FALSE
TCGA-A8-A08J	7	107556063	A	C	DLD	p.Q266P	Missense_Mutation	FALSE	.	0.136904762	0	168	139	0.92	0.71105	1	0.596330024	Clonal	passenger	D	N	T	Passenger	FALSE	FALSE	FALSE
TCGA-E9-A1NI	9	18829842	T	TC	ADAMTSL1	p.Q1375Pfs*53	Frame_Shift_Ins	FALSE	loh	0.137254902	0.012048193	51	83	0.47	0.03833	0.857444894	0.230411297	Subclonal	likely_pathogenic	D	.	.	.	FALSE	FALSE	FALSE
TCGA-D8-A1XZ	8	97620629	G	A	SDC2	p.E125K	Missense_Mutation	FALSE	.	0.137931034	0	145	72	0.92	0.6951	1	0.574653419	Clonal	passenger	N	N	T	Passenger	FALSE	FALSE	FALSE
TCGA-E9-A1QZ	5	34024111	C	A	C1QTNF3	p.V235L	Missense_Mutation	FALSE	.	0.137931034	0	58	53	1	0.8079	1	0.459747917	Clonal	passenger	D	N	T	Passenger	FALSE	FALSE	FALSE
TCGA-A7-A4SC	8	61655528	A	T	CHD7	p.T513S	Missense_Mutation	FALSE	.	0.138888889	0	36	27	0.63	0.40388	0.970828863	0.266150748	Subclonal	passenger	N	N	T	Passenger	FALSE	FALSE	FALSE
TCGA-D8-A1XY	23	38156690	A	G	RPGR	p.S421P	Missense_Mutation	FALSE	.	0.138888889	0	72	58	0.91	0.72974	0.989807169	0.462457955	Clonal	passenger	N	N	T	Passenger	FALSE	FALSE	FALSE
TCGA-E2-A15R	19	2271899	G	C	OAZ1	p.G137A	Missense_Mutation	FALSE	.	0.138888889	0	72	69	0.33	0	0.557769051	0.179058103	Subclonal	passenger	N	.	T	Passenger	FALSE	FALSE	FALSE
TCGA-D8-A1XZ	12	26628350	A	C	ITPR2	p.V2074G	Missense_Mutation	FALSE	.	0.139534884	0.024390244	43	82	0.51	0.1488	0.918445768	0.236402841	Subclonal	likely_pathogenic	D	D	D	Passenger	FALSE	FALSE	FALSE
TCGA-E9-A1RI	1	19549138	A	T	EMC1	p.I856N	Missense_Mutation	FALSE	.	0.139534884	0	43	23	0.62	0.3267	0.966281676	0.283687827	Subclonal	likely_pathogenic	D	D	T	Driver	FALSE	FALSE	FALSE
TCGA-A2-A0T6	1	46740307	A	C	RAD54L	p.N596T	Missense_Mutation	FALSE	.	0.14	0.011494253	50	87	0.55	0.19561	0.935289925	0.27047177	Subclonal	passenger	D	D	T	Passenger	FALSE	FALSE	FALSE
TCGA-AR-A0TW	20	61834086	G	C	YTHDF1	p.I402M	Missense_Mutation	FALSE	.	0.142857143	0	21	17	0.6	0.51055	0.97177515	0.198627342	Clonal	passenger	D	D	T	Passenger	FALSE	FALSE	FALSE
TCGA-D8-A1XZ	11	6891847	C	G	OR10A2	p.L288V	Missense_Mutation	FALSE	.	0.142857143	0	35	78	0.38	0.02383	0.772373148	0.16384581	Subclonal	passenger	N	N	T	Passenger	FALSE	FALSE	FALSE
TCGA-E9-A1QZ	9	111670661	T	C	IKBKAP	p.T462A	Missense_Mutation	FALSE	.	0.142857143	0	56	39	1	0.81589	1	0.468335909	Clonal	passenger	N	N	T	Passenger	FALSE	FALSE	FALSE
TCGA-E9-A1RI	13	20625633	G	A	ZMYM2	p.G785R	Missense_Mutation	FALSE	.	0.142857143	0	77	62	0.63	0.23908	0.952744633	0.357492009	Subclonal	passenger	D	.	T	Passenger	TRUE	FALSE	FALSE
TCGA-A2-A0T6	2	9517076	G	A	ASAP2	p.V596M	Missense_Mutation	FALSE	.	0.143884892	0	139	109	0.57	0.0181	0.829282307	0.371102905	Subclonal	passenger	D	N	T	Passenger	FALSE	FALSE	FALSE
TCGA-AR-A0TW	4	177077232	G	T	WDR17	p.W845C	Missense_Mutation	FALSE	.	0.145299145	0	234	85	0.9	0.78101	1	0.63573702	Clonal	passenger	D	D	T	Passenger	FALSE	FALSE	FALSE
TCGA-BH-A1FM	6	31498921	G	T	DDX39B	p.T344N	Missense_Mutation	FALSE	.	0.145833333	0	48	51	0.69	0.5281	0.976094629	0.331399261	Clonal	passenger	D	D	T	Passenger	FALSE	FALSE	FALSE
TCGA-A8-A08J	20	36993284	A	G	LBP	p.M267V	Missense_Mutation	FALSE	.	0.146258503	0	294	90	1	0.87377	1	0.80147333	Clonal	passenger	N	N	T	Passenger	FALSE	FALSE	FALSE
TCGA-D8-A1XY	22	35948068	A	C	RASD2	p.T264P	Missense_Mutation	FALSE	.	0.148148148	0	27	18	0.97	0.70727	0.987952582	0.315736429	Clonal	passenger	N	N	T	Passenger	FALSE	FALSE	FALSE
TCGA-D8-A1XZ	14	24788604	G	C	ADCY4	p.I924M	Missense_Mutation	FALSE	.	0.148717949	0	195	161	0.99	0.77932	1	0.660107997	Clonal	likely_pathogenic	D	D	D	Passenger	FALSE	FALSE	FALSE
TCGA-D8-A1JN	10	72633267	G	T	SGPL1	p.A407S	Missense_Mutation	FALSE	.	0.15	0	60	28	0.35	0.0001	0.604205965	0.18454041	Subclonal	passenger	D	N	T	Passenger	FALSE	FALSE	FALSE
TCGA-E2-A15H	7	1586811	G	T	TMEM184A	p.P340Q	Missense_Mutation	FALSE	.	0.15	0	20	23	0.69	0.59513	0.978764075	0.219538054	Clonal	passenger	N	N	T	Passenger	FALSE	FALSE	FALSE
TCGA-E9-A1NI	10	8007026	C	T	TAF3	p.S518F	Missense_Mutation	FALSE	.	0.15	0	20	17	0.66	0.35039	0.977339279	0.214397707	Subclonal	passenger	D	N	T	Passenger	FALSE	FALSE	FALSE
TCGA-E9-A1RI	14	64194161	C	G	SGPP1	p.V168L	Missense_Mutation	FALSE	.	0.15	0	20	12	0.67	0.43443	0.977549322	0.215102951	Subclonal	passenger	D	N	T	Passenger	FALSE	FALSE	FALSE
TCGA-E9-A54Y	4	87967857	G	T	AFF1	p.G57V	Missense_Mutation	FALSE	.	0.15	0	20	40	0.6	0.47694	0.972157598	0.200185967	Subclonal	likely_pathogenic	D	D	T	Driver	TRUE	FALSE	FALSE
TCGA-BH-A1FM	17	48600307	T	G	MYCBPAP	p.V465G	Missense_Mutation	FALSE	loh	0.151515152	0.022222222	33	45	0.72	0.57702	0.980054327	0.296033016	Clonal	passenger	N	D	T	Passenger	FALSE	FALSE	FALSE
TCGA-BH-A1FM	1	170961469	C	T	MROH9	p.A398V	Missense_Mutation	FALSE	.	0.152173913	0.025316456	92	79	0.87	0.71895	0.989358666	0.504850294	Clonal	passenger	N	D	T	Passenger	FALSE	FALSE	FALSE
TCGA-E9-A1R0	13	103343304	C	G	METTL21C	p.K47N	Missense_Mutation	FALSE	.	0.152380952	0	105	141	1	0.89189	1	0.639458276	Clonal	passenger	N	N	T	Passenger	FALSE	FALSE	FALSE
TCGA-AR-A24O	17	7217862	CTCTCCTGT	C	GPS2	p.Q48Kfs*46	Frame_Shift_Del	FALSE	loh	0.152941176	0	85	105	1	0.82971	1	0.559433345	Clonal	likely_pathogenic	.	.	.	.	FALSE	FALSE	TRUE
TCGA-AR-A0TW	15	101555571	A	G	LRRK1	p.T525A	Missense_Mutation	FALSE	.	0.154761905	0	84	61	0.8	0.6619	0.984980873	0.463065547	Clonal	passenger	N	N	T	Passenger	FALSE	FALSE	FALSE
TCGA-E9-A1R0	17	56581815	T	C	MTMR4	p.D445G	Missense_Mutation	FALSE	.	0.155555556	0.029850746	45	67	1	0.86058	1	0.475650691	Clonal	likely_pathogenic	D	D	D	Driver	FALSE	FALSE	FALSE
TCGA-D8-A1XY	3	193068972	CAT	C	ATP13A5	p.Y208Cfs*36	Frame_Shift_Del	FALSE	.	0.15625	0	96	56	1	0.84768	1	0.61219683	Clonal	passenger	.	.	.	.	FALSE	FALSE	FALSE
TCGA-E9-A1QZ	17	78073416	C	A	CCDC40	p.Q1091K	Missense_Mutation	FALSE	.	0.15625	0	32	20	1	0.79073	1	0.409539508	Clonal	passenger	N	N	T	Passenger	FALSE	FALSE	FALSE
TCGA-E2-A15H	7	4153004	C	A	SDK1	p.P1173H	Missense_Mutation	FALSE	.	0.156862745	0	102	87	0.72	0.48972	0.96994619	0.445165288	Subclonal	likely_pathogenic	D	D	T	Driver	FALSE	FALSE	FALSE
TCGA-E2-A15H	11	74651907	A	C	XRRA1	p.I6S	Missense_Mutation	FALSE	.	0.157407407	0	108	100	0.88	0.75194	0.989819659	0.53558488	Clonal	passenger	N	N	T	Passenger	FALSE	FALSE	FALSE
TCGA-AR-A0TW	6	51524412	C	A	PKHD1	p.Q3504H	Missense_Mutation	FALSE	.	0.158878505	0	107	57	0.82	0.68393	0.986379531	0.510154415	Clonal	likely_pathogenic	D	D	D	Passenger	FALSE	FALSE	FALSE
TCGA-AR-A0TW	5	140262304	C	T	PCDHA13	p.R151*	Nonsense_Mutation	FALSE	.	0.159090909	0	88	44	0.98	0.8011	1	0.547002543	Clonal	passenger	A	.	.	.	FALSE	FALSE	FALSE
TCGA-AR-A0TW	1	103463920	C	A	COL11A1	p.X715_splice	Splice_Site	FALSE	.	0.16	0	50	33	0.83	0.68649	0.986512973	0.402548924	Clonal	passenger	D	.	.	.	FALSE	FALSE	FALSE
TCGA-E9-A1QZ	23	107823802	A	G	COL4A5	p.I274V	Missense_Mutation	FALSE	.	0.16	0	100	92	1	0.88393	1	0.631977766	Clonal	passenger	N	N	D	Passenger	FALSE	FALSE	FALSE
TCGA-E9-A54Y	20	47589751	G	A	ARFGEF2	p.R532H	Missense_Mutation	FALSE	.	0.16	0	50	64	0.96	0.72836	1	0.442505662	Clonal	passenger	D	D	T	Passenger	FALSE	FALSE	FALSE
TCGA-A7-A4SC	5	133644285	T	G	CDKL3	p.S339R	Missense_Mutation	FALSE	.	0.161290323	0	31	45	0.73	0.51476	0.980885487	0.300648577	Clonal	passenger	N	N	T	Passenger	FALSE	FALSE	FALSE
TCGA-E9-A1R0	2	61575497	G	T	USP34	p.A598E	Missense_Mutation	FALSE	.	0.161290323	0.011235955	31	89	1	0.8524	1	0.408451789	Clonal	passenger	D	N	T	Passenger	FALSE	FALSE	FALSE
TCGA-E2-A155	17	57057854	C	T	PPM1E	p.A577V	Missense_Mutation	FALSE	.	0.161953728	0.005952381	389	168	0.89	0.72803	1	0.694006349	Clonal	passenger	N	N	T	Passenger	FALSE	FALSE	FALSE
TCGA-A7-A4SC	16	68845616	GAC	G	CDH1	p.D288Gfs*4	Frame_Shift_Del	FALSE	loh	0.162790698	0	43	76	0.58	0.30992	0.944805796	0.28221184	Subclonal	likely_pathogenic	.	.	.	.	TRUE	TRUE	TRUE
TCGA-D8-A1JN	16	30732785	G	T	SRCAP	p.A1177S	Missense_Mutation	FALSE	.	0.162790698	0	43	28	0.38	0.00383	0.694562156	0.185503857	Subclonal	likely_pathogenic	D	N	D	Passenger	FALSE	FALSE	FALSE
TCGA-D8-A1JN	16	708927	C	A	WDR90	p.A976D	Missense_Mutation	FALSE	.	0.163265306	0	49	17	0.38	0.00158	0.672571328	0.194442902	Subclonal	passenger	D	N	T	Passenger	FALSE	FALSE	FALSE
TCGA-E9-A54Y	1	158390002	C	A	OR10K2	p.V219F	Missense_Mutation	FALSE	loh	0.163934426	0	61	55	1	0.86894	1	0.580260689	Clonal	passenger	N	N	T	Passenger	FALSE	FALSE	FALSE
TCGA-E2-A15H	1	79115985	C	A	IFI44	p.F35L	Missense_Mutation	FALSE	.	0.164670659	0	334	258	0.92	0.82012	1	0.701784712	Clonal	passenger	N	D	T	Passenger	FALSE	FALSE	FALSE
TCGA-E2-A155	17	64875098	C	T	CACNG5	p.R69W	Missense_Mutation	FALSE	.	0.16509434	0	212	114	0.9	0.76391	1	0.644116414	Clonal	likely_pathogenic	D	D	D	Passenger	FALSE	FALSE	FALSE
TCGA-AR-A0TW	23	135445675	G	T	GPR112	p.K2439N	Missense_Mutation	FALSE	.	0.165680473	0	169	141	0.86	0.72597	0.988785633	0.592431615	Clonal	passenger	N	N	T	Passenger	FALSE	FALSE	FALSE
TCGA-A2-A0T6	11	65339159	G	A	SSSCA1	p.R185H	Missense_Mutation	FALSE	.	0.166666667	0.027027027	30	37	0.66	0.42172	0.974096063	0.277472188	Subclonal	passenger	D	N	T	Passenger	FALSE	FALSE	FALSE
TCGA-AR-A24O	15	65681634	T	G	IGDCC4	p.T842P	Missense_Mutation	FALSE	.	0.166666667	0.058823529	30	17	1	0.85272	1	0.42531893	Clonal	passenger	D	D	T	Passenger	FALSE	FALSE	FALSE
TCGA-BH-A1FM	19	18245637	C	T	MAST3	p.T543M	Missense_Mutation	FALSE	.	0.166666667	0	78	88	0.79	0.62684	0.983852517	0.458596262	Clonal	likely_pathogenic	D	D	T	Driver	FALSE	FALSE	FALSE
TCGA-D8-A1XC	17	45219311	T	C	CDC27	p.I493V	Missense_Mutation	FALSE	.	0.166666667	0	18	19	0.4	0.09984	0.886636916	0.139687616	Subclonal	passenger	D	N	T	Passenger	FALSE	FALSE	TRUE
TCGA-D8-A1XY	5	110679796	A	C	CAMK4	p.K79T	Missense_Mutation	FALSE	.	0.166666667	0	78	81	1	0.82008	1	0.565513259	Clonal	passenger	D	D	T	Passenger	FALSE	FALSE	FALSE
TCGA-E2-A15R	22	44585003	C	G	PARVG	p.A86G	Missense_Mutation	FALSE	.	0.166666667	0.008403361	72	119	0.39	0.00006	0.633643982	0.227952169	Subclonal	passenger	N	N	D	Passenger	FALSE	FALSE	FALSE
TCGA-E9-A1NI	5	176003133	T	TC	CDHR2	p.R383Pfs*6	Frame_Shift_Ins	FALSE	.	0.166666667	0	18	34	0.9	0.47691	0.985796194	0.258313861	Subclonal	passenger	D	.	.	.	FALSE	FALSE	FALSE
TCGA-E9-A1QZ	10	81921705	G	T	ANXA11	p.A389D	Missense_Mutation	FALSE	.	0.166666667	0	18	21	1	0.75833	0.989380845	0.295652933	Clonal	passenger	D	N	T	Passenger	FALSE	FALSE	FALSE
TCGA-E9-A1QZ	16	88677691	CGA	C	ZC3H18	p.N412Qfs*44	Frame_Shift_Del	FALSE	loh	0.166666667	0	12	10	1	0.72378	0.987276725	0.221147097	Clonal	likely_pathogenic	D	.	.	.	FALSE	FALSE	FALSE
TCGA-E9-A1RB	23	128649967	G	A	SMARCA1	p.R145C	Missense_Mutation	FALSE	loh	0.168674699	0	83	93	0.7	0.43415	0.966944696	0.42050813	Subclonal	likely_pathogenic	D	D	D	Passenger	FALSE	FALSE	FALSE
TCGA-AR-A0TW	15	101555577	A	T	LRRK1	p.R527*	Nonsense_Mutation	FALSE	.	0.168831169	0	77	59	0.88	0.73537	0.989324784	0.496112527	Clonal	passenger	A	.	.	.	FALSE	FALSE	FALSE
TCGA-E9-A1R0	14	20927414	C	A	TMEM55B	p.C221F	Missense_Mutation	FALSE	.	0.169230769	0.026548673	65	113	1	0.89802	1	0.589906054	Clonal	passenger	D	N	.	Passenger	FALSE	FALSE	FALSE
TCGA-E9-A1RB	21	31933503	CCA	C	KRTAP19-7	p.L35Rfs*39	Frame_Shift_Del	FALSE	loh	0.172043011	0	93	116	0.71	0.4408	0.967885024	0.44337643	Subclonal	likely_pathogenic	D	.	.	.	FALSE	FALSE	FALSE
TCGA-E9-A54Y	11	35282449	C	T	SLC1A2	p.E573K	Missense_Mutation	FALSE	loh	0.172413793	0	58	87	0.87	0.68811	0.988423986	0.451632395	Clonal	passenger	D	N	T	Passenger	FALSE	FALSE	FALSE
TCGA-D8-A1XY	12	133733005	T	G	ZNF10	p.H391Q	Missense_Mutation	FALSE	.	0.173076923	0	52	41	1	0.80417	1	0.505281335	Clonal	passenger	D	N	T	Passenger	FALSE	FALSE	FALSE
TCGA-A7-A4SC	23	105179171	G	T	NRK	p.G1171V	Missense_Mutation	FALSE	.	0.173333333	0	75	72	0.79	0.54793	0.983363993	0.456999236	Clonal	passenger	N	N	T	Passenger	FALSE	FALSE	FALSE
TCGA-D8-A1XZ	3	129251571	G	A	RHO	p.A298T	Missense_Mutation	FALSE	.	0.173333333	0	75	109	0.63	0.1958	0.939869254	0.375475821	Subclonal	passenger	N	N	T	Passenger	FALSE	FALSE	FALSE
TCGA-D8-A1XZ	23	46434009	G	A	CHST7	p.A215T	Missense_Mutation	FALSE	.	0.173913043	0	23	15	0.81	0.54861	0.984390073	0.288153617	Clonal	passenger	N	N	D	Passenger	FALSE	FALSE	FALSE
TCGA-A7-A4SC	19	58797478	C	T	ZNF8	p.P66S	Missense_Mutation	FALSE	.	0.175675676	0	74	79	0.8	0.56391	0.984364243	0.462280659	Clonal	passenger	D	.	T	Passenger	FALSE	FALSE	FALSE
TCGA-AR-A0TW	16	3432988	T	C	ZSCAN32	p.K441R	Missense_Mutation	FALSE	.	0.175879397	0	199	50	1	0.89525	1	0.727033514	Clonal	passenger	N	N	T	Passenger	FALSE	FALSE	FALSE
TCGA-D8-A1XY	3	11301339	A	G	HRH1	p.M206V	Missense_Mutation	FALSE	.	0.176056338	0	142	164	0.97	0.8236	1	0.635430478	Clonal	passenger	D	D	T	Passenger	FALSE	FALSE	FALSE
TCGA-D8-A1XZ	14	93118595	A	T	RIN3	p.S401C	Missense_Mutation	FALSE	.	0.176470588	0	17	40	0.64	0.43563	0.975678502	0.211473309	Subclonal	passenger	D	N	T	Passenger	FALSE	FALSE	FALSE
TCGA-E2-A155	6	123851667	C	A	TRDN	p.R156S	Missense_Mutation	FALSE	.	0.177394035	0	637	156	0.79	0.09265	0.928528204	0.665031555	Subclonal	passenger	N	N	T	Passenger	FALSE	FALSE	FALSE
TCGA-E2-A15R	7	100634947	C	G	MUC12	p.T368S	Missense_Mutation	FALSE	.	0.177506775	0	738	699	0.6	0	0.691607119	0.505562216	Subclonal	passenger	N	N	T	Passenger	FALSE	FALSE	FALSE
TCGA-A8-A08J	1	28802626	G	A	PHACTR4	p.D487N	Missense_Mutation	FALSE	.	0.178571429	0	168	169	0.85	0.60327	0.987283939	0.593492998	Clonal	passenger	D	D	T	Passenger	FALSE	FALSE	FALSE
TCGA-D8-A1XZ	16	31391336	G	A	ITGAX	p.A1004T	Missense_Mutation	FALSE	.	0.178571429	0	28	34	0.83	0.5681	0.985649382	0.329142483	Clonal	passenger	N	N	T	Passenger	FALSE	FALSE	FALSE
TCGA-AR-A0TW	1	182025629	C	A	ZNF648	p.C506F	Missense_Mutation	FALSE	.	0.179487179	0	39	22	1	0.79356	1	0.451626398	Clonal	likely_pathogenic	D	D	D	Passenger	FALSE	FALSE	FALSE
TCGA-A2-A0T6	15	65681634	T	G	IGDCC4	p.T842P	Missense_Mutation	FALSE	.	0.181818182	0.011235955	22	89	0.72	0.49233	0.980121449	0.266872505	Subclonal	passenger	D	D	T	Passenger	FALSE	FALSE	FALSE
TCGA-A2-A3KD	10	8115732	G	GGC	GATA3	p.I361Afs*44	Frame_Shift_Ins	FALSE	.	0.181818182	0	11	18	0.78	0.5137	0.981709111	0.183528971	Subclonal	likely_pathogenic	D	.	.	.	TRUE	TRUE	TRUE

TCGA-E9-A54Y	20	44756975	T	G	CD40	p.V218G	Missense_Mutation	FALSE	.	0.181818182	0	22	25	1	0.72864	0.989602633	0.336889491	Clonal	passenger	N	N	T	Passenger	FALSE	FALSE	FALSE
TCGA-E9-A54Y	6	42897357	T	TTGC	CNPY3	p.L25dup	In_Frame_Ins	FALSE	.	0.181818182	0.022727273	44	44	1	0.81631	1	0.507217626	Clonal	passenger	N	.	.	.	FALSE	FALSE	FALSE
TCGA-D8-A1XZ	1	44363942	G	C	ST3GAL3	p.E214Q	Missense_Mutation	FALSE	.	0.182926829	0	82	71	1	0.80376	1	0.624086541	Clonal	passenger	D	N	T	Passenger	FALSE	FALSE	FALSE
TCGA-BH-A1FM	16	78420808	G	A	WWOX	p.V190M	Missense_Mutation	FALSE	.	0.183098592	0.020833333	71	96	1	0.80883	1	0.557616984	Clonal	passenger	D	N	T	Passenger	FALSE	FALSE	FALSE
TCGA-D8-A1XY	2	228881454	C	A	SPHKAP	p.R1372S	Missense_Mutation	FALSE	.	0.183098592	0	71	56	1	0.79894	1	0.546146266	Clonal	passenger	N	N	T	Passenger	FALSE	FALSE	FALSE
TCGA-D8-A1XY	2	216262532	C	A	FN1	p.E1130*	Nonsense_Mutation	FALSE	.	0.183908046	0	87	99	1	0.81548	1	0.583294219	Clonal	passenger	A	.	.	.	FALSE	FALSE	FALSE
TCGA-D8-A1JN	15	72873082	C	A	ARIH1	p.A409E	Missense_Mutation	TRUE	.	0.184615385	0	65	34	0.43	0.00075	0.684189823	0.249333535	Subclonal	likely_pathogenic	D	N	D	Passenger	FALSE	FALSE	FALSE
TCGA-A7-A4SC	22	38710134	C	A	CSNK1E	p.R10L	Missense_Mutation	FALSE	loh	0.185185185	0	27	47	0.65	0.51479	0.973308695	0.277570178	Clonal	likely_pathogenic	D	D	T	Driver	FALSE	FALSE	FALSE
TCGA-D8-A1XZ	4	37448786	C	T	KIAA1239	p.R1726W	Missense_Mutation	FALSE	.	0.185185185	0	27	37	0.68	0.43631	0.975972514	0.284924236	Subclonal	passenger	N	N	D	Passenger	FALSE	FALSE	FALSE
TCGA-A8-A08J	19	41863814	T	C	B9D2	p.K68E	Missense_Mutation	FALSE	.	0.1875	0	32	35	1	0.69508	1	0.415020363	Clonal	passenger	D	N	T	Passenger	FALSE	FALSE	FALSE
TCGA-A8-A08J	21	45987772	C	CG	TSPEAR	p.R67Pfs*15	Frame_Shift_Ins	FALSE	.	0.1875	0	16	46	0.89	0.5878	0.985545667	0.257395853	Clonal	passenger	D	.	.	.	FALSE	FALSE	FALSE
TCGA-BH-A1FM	9	140395296	T	C	PNPLA7	p.K535R	Missense_Mutation	FALSE	.	0.1875	0	16	23	1	0.72386	0.988113666	0.280719323	Clonal	likely_pathogenic	D	N	D	Passenger	FALSE	FALSE	FALSE
TCGA-D8-A1XZ	11	55579837	G	C	OR5L1	p.E299Q	Missense_Mutation	FALSE	.	0.1875	0	16	38	0.5	0.3179	0.948511635	0.171919676	Subclonal	passenger	N	N	T	Passenger	FALSE	FALSE	FALSE
TCGA-E2-A15R	15	55731773	G	T	DYX1C1	p.H264N	Missense_Mutation	FALSE	.	0.1875	0.013986014	80	143	0.44	0.00014	0.67495667	0.272810677	Subclonal	passenger	N	N	T	Passenger	FALSE	FALSE	FALSE
TCGA-E9-A1NI	5	167988433	T	A	PANK3	p.I301F	Missense_Mutation	TRUE	.	0.1875	0	32	62	1	0.56659	1	0.403966312	Clonal	likely_pathogenic	D	D	D	Passenger	FALSE	FALSE	FALSE
TCGA-E9-A1R0	1	4832365	C	T	AJAP1	p.R315W	Missense_Mutation	FALSE	.	0.1875	0	16	44	1	0.84742	1	0.314790893	Clonal	passenger	N	D	T	Passenger	FALSE	FALSE	FALSE
TCGA-A8-A08J	16	28507445	G	C	APOBR	p.E361D	Missense_Mutation	FALSE	.	0.188679245	0	53	20	1	0.83894	1	0.580651155	Clonal	passenger	N	N	T	Passenger	FALSE	FALSE	FALSE
TCGA-BH-A1FM	7	88956679	C	T	ZNF804B	p.R91W	Missense_Mutation	FALSE	.	0.188679245	0	53	70	1	0.79866	1	0.517631861	Clonal	passenger	D	D	T	Passenger	FALSE	FALSE	FALSE
TCGA-A7-A4SC	1	197026274	G	T	F13B	p.A347E	Missense_Mutation	FALSE	.	0.189655172	0	58	33	1	0.79632	1	0.568699443	Clonal	passenger	N	N	T	Passenger	FALSE	FALSE	FALSE
TCGA-D8-A1XY	14	93043720	C	T	RIN3	p.R89W	Missense_Mutation	FALSE	.	0.189655172	0	58	71	1	0.79907	1	0.527095302	Clonal	passenger	D	D	T	Passenger	FALSE	FALSE	FALSE
TCGA-E9-A1R0	12	53776179	G	C	SP1	p.A150P	Missense_Mutation	FALSE	.	0.19047619	0	21	41	1	0.86908	1	0.380529358	Clonal	passenger	D	N	T	Passenger	FALSE	FALSE	FALSE
TCGA-D8-A1XZ	11	30926690	C	A	DCDC1	p.A1376S	Missense_Mutation	FALSE	.	0.191780822	0	73	95	0.7	0.33941	0.966409879	0.423098634	Subclonal	passenger	N	.	.	Passenger	FALSE	FALSE	FALSE
TCGA-D8-A1XZ	6	80198927	G	A	LCA5	p.Q369*	Nonsense_Mutation	FALSE	.	0.192090395	0	177	204	0.7	0.10425	0.920680277	0.509670571	Subclonal	passenger	A	.	.	.	FALSE	FALSE	FALSE
TCGA-D8-A1XZ	11	101359736	C	T	TRPC6	p.V409M	Missense_Mutation	FALSE	.	0.192307692	0	26	50	0.7	0.4648	0.978437496	0.293887179	Subclonal	passenger	D	N	T	Passenger	FALSE	FALSE	FALSE
TCGA-E9-A1R0	17	36889548	A	C	CISD3	p.H75P	Missense_Mutation	FALSE	.	0.192307692	0.029411765	26	68	1	0.8849	1	0.433539911	Clonal	passenger	D	.	.	Passenger	FALSE	FALSE	FALSE
TCGA-A8-A08J	1	11888625	C	G	CLCN6	p.N355K	Missense_Mutation	FALSE	.	0.192825112	0	223	210	0.91	0.72906	1	0.675248043	Clonal	likely_pathogenic	D	D	D	Passenger	FALSE	FALSE	FALSE
TCGA-D8-A1XZ	19	14031724	G	C	CC2D1A	p.D544H	Missense_Mutation	FALSE	.	0.193548387	0	31	32	0.9	0.61662	0.988251255	0.378646	Clonal	passenger	D	D	T	Passenger	FALSE	FALSE	FALSE
TCGA-E9-A54Y	11	18195222	C	T	MRGPRX4	p.A140V	Missense_Mutation	FALSE	loh	0.194444444	0	72	91	0.98	0.77611	1	0.548446773	Clonal	passenger	N	D	T	Passenger	FALSE	FALSE	FALSE
TCGA-E9-A1RI	9	17466011	G	T	CNTLN	p.K1188N	Missense_Mutation	FALSE	.	0.195121951	0	41	33	0.87	0.59094	0.988046019	0.418392997	Clonal	passenger	D	N	T	Passenger	FALSE	FALSE	FALSE
TCGA-AR-A0TW	19	53572793	G	A	ZNF160	p.R332W	Missense_Mutation	FALSE	.	0.195652174	0	138	62	1	0.8974	1	0.725444285	Clonal	passenger	N	D	T	Passenger	FALSE	FALSE	FALSE
TCGA-E9-A1QZ	10	71175920	C	T	TACR2	p.V54I	Missense_Mutation	FALSE	.	0.195652174	0	46	38	1	0.8557	1	0.560612448	Clonal	likely_pathogenic	D	N	D	Passenger	FALSE	FALSE	FALSE
TCGA-D8-A1XY	19	19645891	G	A	CTC-260F20.3	p.V122M	Missense_Mutation	FALSE	.	0.196078431	0	51	53	1	0.80228	1	0.518710666	Clonal	passenger	D	N	T	Passenger	FALSE	FALSE	FALSE
TCGA-AR-A0TW	5	153065885	G	A	GRIA1	p.R387Q	Missense_Mutation	FALSE	loh	0.196721311	0	61	38	0.82	0.6879	0.986456069	0.465277027	Clonal	passenger	D	N	T	Passenger	FALSE	FALSE	FALSE
TCGA-BH-A1FM	23	54817311	G	A	ITIH6	p.P192L	Missense_Mutation	FALSE	.	0.2	0	40	58	1	0.79984	1	0.488193243	Clonal	passenger	N	N	T	Passenger	FALSE	FALSE	FALSE
TCGA-E9-A1NI	11	46917494	G	GC	LRP4	p.A375Gfs*24	Frame_Shift_Ins	FALSE	.	0.2	0	15	35	0.88	0.46936	0.98551346	0.257806468	Subclonal	passenger	D	.	.	.	FALSE	FALSE	FALSE
TCGA-E9-A1NI	11	73021526	G	GC	ARHGEF17	p.G618Wfs*4	Frame_Shift_Ins	FALSE	.	0.2	0	15	46	0.88	0.46936	0.98551346	0.257806468	Subclonal	passenger	D	.	.	.	FALSE	FALSE	FALSE
TCGA-A7-A4SC	19	14817558	C	T	ZNF333	p.R162C	Missense_Mutation	FALSE	.	0.204545455	0	44	47	0.93	0.67624	1	0.458272619	Clonal	passenger	N	N	T	Passenger	FALSE	FALSE	FALSE
TCGA-BH-A1FM	11	85966327	G	A	EED	p.D142N	Missense_Mutation	FALSE	.	0.205673759	0.006896552	141	145	1	0.89279	1	0.730664082	Clonal	passenger	D	D	T	Passenger	FALSE	FALSE	FALSE
TCGA-E9-A1NI	7	128610258	A	AG	TNPO3	p.Y848Lfs*8	Frame_Shift_Ins	FALSE	.	0.205882353	0	34	43	0.91	0.52841	0.98902367	0.409617244	Clonal	passenger	D	.	.	.	FALSE	FALSE	FALSE
TCGA-A8-A08J	23	101858458	C	A	ARMCX5	p.H463Q	Missense_Mutation	FALSE	.	0.206293706	0.001757469	572	569	1	0.91347	1	0.890487644	Clonal	passenger	N	N	T	Passenger	FALSE	FALSE	FALSE
TCGA-AR-A24O	12	48482684	C	T	SENP1	p.A94T	Missense_Mutation	FALSE	.	0.20754717	0	106	113	1	0.95943	1	0.77338484	Clonal	passenger	D	N	T	Passenger	FALSE	FALSE	FALSE
TCGA-BH-A1FM	13	103301465	A	C	TPP2	p.N946T	Missense_Mutation	FALSE	.	0.20754717	0.020408163	53	49	1	0.83094	1	0.561932378	Clonal	passenger	D	N	.	Passenger	FALSE	FALSE	FALSE
TCGA-AR-A0TW	5	137476527	C	T	BRD8	p.R1161Q	Missense_Mutation	FALSE	.	0.20952381	0	105	55	1	0.89086	1	0.711618844	Clonal	passenger	N	N	T	Passenger	FALSE	FALSE	FALSE
TCGA-D8-A1XY	3	46560544	G	A	LRRC2	p.P359L	Missense_Mutation	FALSE	.	0.209876543	0	81	99	1	0.8581	1	0.637772587	Clonal	passenger	D	N	T	Passenger	FALSE	FALSE	FALSE
TCGA-AR-A0TW	23	153993215	A	G	DKC1	p.K20E	Missense_Mutation	FALSE	.	0.210084034	0	357	241	1	0.93424	1	0.82237665	Clonal	passenger	N	N	D	Passenger	FALSE	FALSE	FALSE
TCGA-E2-A155	1	17275382	G	A	CROCC	p.E933K	Missense_Mutation	FALSE	.	0.210526316	0	19	24	0.52	0.28092	0.942276309	0.206811773	Subclonal	passenger	D	D	T	Passenger	FALSE	FALSE	FALSE
TCGA-D8-A1XZ	23	70832732	C	T	ACRC	p.S659L	Missense_Mutation	FALSE	.	0.211111111	0	90	107	0.77	0.45783	0.978421994	0.500205574	Subclonal	passenger	D	D	T	Passenger	FALSE	FALSE	FALSE
TCGA-E2-A15H	4	88584456	GC	G	DMP1	p.Q510Kfs*22	Frame_Shift_Del	FALSE	.	0.211111111	0	90	75	0.97	0.82227	1	0.595913018	Clonal	passenger	N	.	.	.	FALSE	FALSE	FALSE
TCGA-AR-A24O	13	113212589	C	T	TUBGCP3	p.V157M	Missense_Mutation	FALSE	.	0.212765957	0	47	47	1	0.93427	1	0.621668317	Clonal	passenger	N	N	T	Passenger	FALSE	FALSE	FALSE
TCGA-D8-A1JN	5	37026380	ACAATGACAAAGAAG	A	NIPBL	p.A1925Nfs*17	Frame_Shift_Del	FALSE	.	0.212765957	0	47	56	0.5	0.02962	0.806762617	0.274884613	Subclonal	passenger	D	.	.	.	FALSE	FALSE	FALSE
TCGA-A8-A08J	19	38911748	G	A	RASGRP4	p.R101C	Missense_Mutation	FALSE	.	0.213114754	0	61	53	1	0.80047	1	0.601495458	Clonal	passenger	D	D	T	Passenger	FALSE	FALSE	FALSE
TCGA-AR-A24O	6	26234971	T	C	HIST1H1D	p.K64R	Missense_Mutation	FALSE	.	0.214285714	0	56	60	1	0.94294	1	0.662655288	Clonal	passenger	D	D	T	Passenger	FALSE	FALSE	FALSE
TCGA-D8-A1XZ	7	101845119	G	A	CUX1	p.G859S	Missense_Mutation	FALSE	.	0.214285714	0	14	25	0.78	0.52827	0.982871708	0.242165365	Clonal	passenger	N	N	T	Passenger	TRUE	FALSE	TRUE
TCGA-E2-A155	3	183967628	G	A	ECE2	p.R49H	Missense_Mutation	FALSE	.	0.214285714	0	14	24	0.53	0.35939	0.956758766	0.182820957	Subclonal	passenger	D	N	T	Passenger	FALSE	FALSE	FALSE
TCGA-E9-A54Y	1	11174931	C	T	MTOR	p.R2368Q	Missense_Mutation	FALSE	.	0.214285714	0	28	45	0.86	0.66794	0.987164541	0.370519144	Clonal	passenger	D	D	T	Passenger	TRUE	TRUE	TRUE
TCGA-E2-A15R	8	62555969	C	T	ASPH	p.D245N	Missense_Mutation	FALSE	.	0.215189873	0	158	94	0.94	0.75576	1	0.665195479	Clonal	passenger	N	N	T	Passenger	FALSE	FALSE	FALSE
TCGA-A7-A4SC	10	89692905	G	A	PTEN	p.R130Q	Missense_Mutation	TRUE	loh	0.218390805	0	87	85	0.77	0.56458	0.978442357	0.501296511	Clonal	likely_pathogenic	A	D	D	Driver	TRUE	TRUE	TRUE
TCGA-A7-A4SC	20	13830252	T	A	SEL1L2	p.X537_splice	Splice_Site	FALSE	.	0.21875	0	32	37	0.99	0.68902	1	0.431411377	Clonal	passenger	D	.	.	.	FALSE	FALSE	FALSE
TCGA-D8-A1XY	11	18566328	T	C	UEVLD	p.Y301C	Missense_Mutation	FALSE	.	0.219047619	0	105	86	1	0.89111	1	0.741493945	Clonal	passenger	D	D	T	Passenger	FALSE	FALSE	FALSE
TCGA-E2-A15H	12	129558738	GC	G	TMEM132D	p.G994Afs*52	Frame_Shift_Del	FALSE	.	0.219047619	0.00625	210	160	1	0.92202	1	0.80542438	Clonal	passenger	D	.	.	.	FALSE	FALSE	FALSE
TCGA-A7-A4SC	12	108011110	C	T	BTBD11	p.R710C	Missense_Mutation	FALSE	.	0.220338983	0	59	64	1	0.74302	1	0.546548545	Clonal	passenger	D	D	T	Passenger	FALSE	FALSE	FALSE
TCGA-D8-A1XY	11	57367733	G	A	SERPING1	p.A145T	Missense_Mutation	FALSE	.	0.220994475	0	181	107	1	0.8301	1	0.824661361	Clonal	passenger	N	N	D	Passenger	FALSE	FALSE	FALSE
TCGA-A2-A0T6	19	58983303	G	T	ZNF324	p.V482L	Missense_Mutation	FALSE	loh	0.222222222	0	9	23	0.88	0.56581	0.983902937	0.196617668	Clonal	passenger	N	N	T	Passenger	FALSE	FALSE	FALSE
TCGA-AR-A0TW	2	198361901	C	A	HSPD1	p.K130N	Missense_Mutation	FALSE	.	0.222222222	0	144	82	1	0.90388	1	0.739612	Clonal	passenger	D	N	T	Passenger	FALSE	FALSE	FALSE
TCGA-E9-A1R0	23	123181291	G	A	STAG2	p.R252Q	Missense_Mutation	FALSE	.	0.222222222	0	63	79	1	0.95335	1	0.688627165	Clonal	likely_pathogenic	D	D	T	Driver	TRUE	TRUE	TRUE
TCGA-AR-A24O	3	178952085	A	T	PIK3CA	p.H1047L	Missense_Mutation	TRUE	.	0.224489796	0	49	35	1	0.9399	1	0.650005757	Clonal	likely_pathogenic	D	N	T	Driver	TRUE	TRUE	TRUE
TCGA-A7-A4SC	1	120465373	G	C	NOTCH2	p.R1630G	Missense_Mutation	FALSE	loh	0.227272727	0	22	23	0.8	0.65305	0.984604898	0.326200612	Clonal	passenger	D	D	T	Passenger	TRUE	FALSE	FALSE
TCGA-AR-A0TW	2	220473425	G	T	STK11IP	p.R586L	Missense_Mutation	FALSE	.	0.227272727	0	22	12	1	0.79118	1	0.397627296	Clonal	passenger	N	D	T	Passenger	FALSE	FALSE	FALSE
TCGA-D8-A1XC	6	137147511	T	G	PEX7	p.H81Q	Missense_Mutation	FALSE	.	0.227272727	0	44	46	0.55	0.05812	0.872706949	0.304018706	Subclonal	passenger	D	D	T	Passenger	FALSE	FALSE	FALSE
TCGA-D8-A1XY	21	47665071	T	G	MCM3AP	p.H1563P	Missense_Mutation	FALSE	.	0.227272727	0.04	22	25	1	0.79758	1	0.407276594	Clonal	passenger	N	N	T	Passenger	FALSE	FALSE	FALSE
TCGA-E9-A1R0	1	155238508	G	T	CLK2	p.Q159K	Missense_Mutation	FALSE	.	0.227272727	0.044444444	22	45	1	0.90427	1	0.453838782	Clonal	passenger	D	N	T	Passenger	FALSE	FALSE	FALSE
TCGA-E9-A1RB	1	151735875	C	A	MRPL9	p.Q27H	Missense_Mutation	FALSE	loh	0.227272727	0	22	10	1	0.87488	1	0.453121796	Clonal	passenger	N	N	T	Passenger	FALSE	FALSE	FALSE
TCGA-E9-A1RB	2	74761688	C	T	LOXL3	p.R598H	Missense_Mutation	FALSE	.	0.229508197	0	61	49	1	0.84805	1	0.609179942	Clonal	passenger	D	D	T	Passenger	FALSE	FALSE	FALSE
TCGA-D8-A1XY	12	51112558	G	T	DIP2B	p.G973V	Missense_Mutation	FALSE	.	0.229927007	0	274	115	1	0.97261	1	0.906789008	Clonal	passenger	D	D	T	Passenger	FALSE	FALSE	FALSE
TCGA-A7-A4SC	19	8807880	C	T	ACTL9	p.R391H	Missense_Mutation	FALSE	.	0.230769231	0	52	62	1	0.75658	1	0.547568416	Clonal	passenger	N	N	T	Passenger	FALSE	FALSE	FALSE
TCGA-AR-A0TW	13	114202762	C	T	TMCO3	p.R639W	Missense_Mutation	FALSE	.	0.230769231	0	26	32	1	0.80617	1	0.439284796	Clonal	passenger	D	D	T	Passenger	FALSE	FALSE	FALSE
TCGA-E9-A1NI	16	4862228	G	GC	GLYR1	p.R381Afs*15	Frame_Shift_Ins	FALSE	.	0.230769231	0	39	44	1	0.82453	1	0.585580985	Clonal	passenger	D	.	.	.	FALSE	FALSE	FALSE
TCGA-E9-A1NI	23	99662746	G	A	PCDH19	p.R284C	Missense_Mutation	FALSE	.	0.230769231	0	39	71	1	0.76422	1	0.564574604	Clonal	passenger	N	D	T	Passenger	FALSE	FALSE	FALSE
TCGA-E9-A1R0	19	49473075	G	T	GYS1	p.A616D	Missense_Mutation	FALSE	.	0.230769231	0	13	51	1	0.86783	1	0.332503658	Clonal	passenger	D	D	T	Passenger	FALSE	FALSE	FALSE
TCGA-E9-A1RB	2	160304785	G	A	BAZ2B	p.S157L	Missense_Mutation	FALSE	.	0.230769231	0	65	84	0.96	0.78628	1	0.557144349	Clonal	passenger	D	N	T	Passenger	FALSE	FALSE	FALSE
TCGA-D8-A1XY	9	115806351	T	A	ZFP37	p.I183F	Missense_Mutation	FALSE	.	0.231404959	0	121	111	1	0.89566	1	0.750093603	Clonal	passenger	N	N	T	Passenger	FALSE	FALSE	FALSE
TCGA-E2-A155	19	46825069	C	T	HIF3A	p.P394L	Missense_Mutation	FALSE	.	0.233333333	0	30	29	0.58	0.24738	0.936846251	0.287076782	Subclonal	passenger	D	D	T	Passenger	FALSE	FALSE	FALSE
TCGA-E2-A15R	15	74032293	T	G	C15orf59	p.T283P	Missense_Mutation	FALSE	.	0.233333333	0.031746032	60	63	0.55	0.02156	0.833117846	0.337508858	Subclonal	passenger	P	N	.	Passenger	FALSE	FALSE	FALSE
TCGA-E9-A54Y	1	35852683	C	A	ZMYM4	p.T639K	Missense_Mutation	FALSE	.	0.233333333	0	30	57	0.94	0.71574	0.98974852	0.4198017	Clonal	passenger	D	N	T	Passenger	FALSE	FALSE	FALSE
TCGA-AR-A24O	1	220091665	G	A	SLC30A10	p.P297L	Missense_Mutation	FALSE	.	0.234375	0	64	49	1	0.94846	1	0.719806661	Clonal	passenger	D	D	T	Passenger	FALSE	FALSE	FALSE
TCGA-BH-A1FM	1	109394679	G	C	AKNAD1	p.A203G	Missense_Mutation	FALSE	.	0.235294118	0	17	39	1	0.75088	0.989982232	0.344295217	Clonal	passenger	N	N	T	Passenger	FALSE	FALSE	FALSE
TCGA-BH-A1FM	10	8111513	T	TG	GATA3	p.D335Gfs*17	Frame_Shift_Ins	FALSE	.	0.235294118	0	102	100	1	0.87442	1	0.687122485	Clonal	likely_pathogenic	.	.	.	.	TRUE	TRUE	TRUE
TCGA-D8-A1XZ	23	105855472	G	A	CXorf57	p.M54I	Missense_Mutation	FALSE	.	0.235294118	0	51	69	0.86	0.62294	0.988490026	0.484588111	Clonal	passenger	N	N	T	Passenger	FALSE	FALSE	FALSE
TCGA-BH-A1FM	5	76028288	C	G	F2R	p.L80V	Missense_Mutation	FALSE	.	0.235772358	0	123	94	1	0.88659	1	0.768907634	Clonal	passenger	N	N	T	Passenger	FALSE	FALSE	FALSE
TCGA-BH-A1FM	5	41049479	C	A	MROH2B	p.E468D	Missense_Mutation	FALSE	.	0.236842105	0	76	59	1	0.88179	1	0.690459346	Clonal	passenger	N	N	T	Passenger	FALSE	FALSE	FALSE
TCGA-E9-A1RB	18	5891633	A	G	TMEM200C	p.S144P	Missense_Mutation	FALSE	.	0.236842105	0	38	43	1	0.82638	1	0.529340744	Clonal	passenger	N	N	.	Passenger	FALSE	FALSE	FALSE
TCGA-AR-A0TW	2	111806831	G	T	ACOXL	p.C469F	Missense_Mutation	FALSE	.	0.237037037	0	135	87	1	0.90798	1	0.760328992	Clonal	passenger	D	D	T	Passenger	FALSE	FALSE	FALSE
TCGA-D8-A1XZ	6	31778018	C	G	HSPA1L	p.E578Q	Missense_Mutation	FALSE	.	0.238095238	0	84	67	0.87	0.64533	0.989502928	0.563089363	Clonal	passenger	D	N	T	Passenger	FALSE	FALSE	FALSE
TCGA-A7-A4SC	3	111805270	C	T	C3orf52	p.P6S	Missense_Mutation	FALSE	.	0.239130435	0	46	63	1	0.76202	1	0.542237452	Clonal	passenger	N	N	T	Passenger	FALSE	FALSE	FALSE
TCGA-A8-A08J	3	5252828	A	G	EDEM1	p.H536R	Missense_Mutation	FALSE	.	0.23943662	0	71	40	1	0.9087	1	0.710942505	Clonal	passenger	D	N	T	Passenger	FALSE	FALSE	FALSE
TCGA-A2-A3KD	3	170201151	G	A	SLC7A14	p.P356L	Missense_Mutation	FALSE	.	0.24	0	50	47	1	0.72652	1	0.542120701	Clonal	likely_pathogenic	D	D	D	Passenger	FALSE	FALSE	FALSE
TCGA-D8-A1XZ	22	41523564	C	T	EP300	p.A327V	Missense_Mutation	FALSE	.	0.24	0	25	44	1	0.70236	1	0.427803267	Clonal	likely_pathogenic	D	N	D	Driver	TRUE	TRUE	TRUE
TCGA-E9-A54Y	9	131347092	A	G	SPTAN1	p.T844A	Missense_Mutation	FALSE	.	0.24	0	75	89	1	0.86076	1	0.661884522	Clonal	passenger	D	N	T	Passenger	FALSE	FALSE	FALSE
TCGA-E9-A1NI	22	50297990	T	TG	ALG12	p.Q386Pfs*9	Frame_Shift_Ins	FALSE	loh	0.24137931	0	29	16	0.58	0.19103	0.93964844	0.290863679	Subclonal	likely_pathogenic	D	.	.	.	FALSE	FALSE	FALSE
TCGA-A2-A3KD	3	8609232	T	C	LMCD1	p.V349A	Missense_Mutation	FALSE	.	0.242424242	0	33	40	1	0.6939	1	0.469303131	Clonal	likely_pathogenic	D	N	D	Passenger	FALSE	FALSE	FALSE
TCGA-E9-A1R0	1	76877751	G	A	ST6GALNAC3	p.G91D	Missense_Mutation	FALSE	.	0.242424242	0	66	107	1	0.95902	1	0.7267041	Clonal	passenger	D	D	T	Passenger	FALSE	FALSE	FALSE
TCGA-E9-A1R0	23	122840715	T	A	THOC2	p.D72V	Missense_Mutation	FALSE	.	0.242718447	0	103	103	1	0.96735	1	0.803093186	Clonal	passenger	D	D	.	Passenger	FALSE	FALSE	FALSE
TCGA-E2-A15R	11	4609000	C	T	OR52I2	p.P320S	Missense_Mutation	FALSE	.	0.243055556	0	576	358	1	0.92917	1	0.869975063	Clonal	likely_pathogenic	D	D	D	Passenger	FALSE	FALSE	FALSE
TCGA-A2-A0T6	13	97485222	C	T	HS6ST3	p.R396C	Missense_Mutation	FALSE	.	0.243243243	0	74	102	0.96	0.73745	1	0.589107969	Clonal	passenger	D	D	T	Passenger	FALSE	FALSE	FALSE
TCGA-AR-A24O	20	40065975	C	G	CHD6	p.X1336_splice	Splice_Site	FALSE	.	0.243589744	0	78	57	1	0.94967	1	0.76755898	Clonal	passenger	D	.	.	.	FALSE	FALSE	FALSE
TCGA-E2-A155	12	90015519	G	T	ATP2B1	p.T465N	Missense_Mutation	FALSE	.	0.243626062	0	353	227	0.6	0	0.716341572	0.494607396	Subclonal	likely_pathogenic	D	D	D	Passenger	FALSE	FALSE	FALSE
TCGA-A7-A4SC	10	71129355	G	A	HK1	p.G288R	Missense_Mutation	FALSE	.	0.244444444	0	45	31	1	0.76947	1	0.54869504	Clonal	likely_pathogenic	D	N	D	Passenger	FALSE	FALSE	FALSE
TCGA-E9-A1R0	1	242383388	C	T	PLD5	p.A213T	Missense_Mutation	FALSE	.	0.244444444	0	45	90	1	0.94844	1	0.650887322	Clonal	passenger	D	N	T	Passenger	FALSE	FALSE	FALSE
TCGA-D8-A1XY	19	51837454	G	A	VSIG10L	p.R804C	Missense_Mutation	FALSE	.	0.244897959	0	49	34	1	0.86752	1	0.611737763	Clonal	passenger	N	D	T	Passenger	FALSE	FALSE	FALSE
TCGA-A8-A08J	2	170367168	G	A	KLHL41	p.D294N	Missense_Mutation	FALSE	.	0.245098039	0	102	91	1	0.85732	1	0.754365646	Clonal	passenger	D	N	T	Passenger	FALSE	FALSE	FALSE
TCGA-E9-A1R0	4	129925014	T	C	SCLT1	p.K103R	Missense_Mutation	FALSE	.	0.245614035	0	171	184	1	0.9743	1	0.871409815	Clonal	passenger	D	N	T	Passenger	FALSE	FALSE	FALSE
TCGA-AR-A24O	23	17705913	C	T	NHS	p.P206L	Missense_Mutation	FALSE	.	0.245901639	0	61	44	1	0.9435	1	0.725061528	Clonal	passenger	D	D	T	Passenger	FALSE	FALSE	FALSE
TCGA-E2-A155	15	102346146	C	T	OR4F6	p.S75F	Missense_Mutation	FALSE	.	0.246458924	0	706	463	0.61	0	0.687872481	0.530480302	Subclonal	passenger	N	D	T	Passenger	FALSE	FALSE	FALSE
TCGA-BH-A1FM	19	13565940	G	T	CACNA1A	p.T127N	Missense_Mutation	FALSE	.	0.246575342	0	73	78	1	0.86434	1	0.654640125	Clonal	passenger	D	D	T	Passenger	FALSE	FALSE	FALSE
TCGA-A2-A0T6	17	37879658	G	A	ERBB2	p.R678Q	Missense_Mutation	TRUE	.	0.247311828	0	93	195	0.98	0.76694	1	0.632961822	Clonal	likely_pathogenic	D	N	T	Driver	TRUE	FALSE	TRUE
TCGA-BH-A1FM	15	89761813	G	A	RLBP1	p.R42C	Missense_Mutation	FALSE	.	0.25	0	24	107	1	0.79285	1	0.43978454	Clonal	passenger	N	N	T	Passenger	FALSE	FALSE	FALSE
TCGA-D8-A1JN	19	51022639	G	T	LRRC4B	p.H111N	Missense_Mutation	FALSE	loh	0.25	0	24	14	0.58	0.35143	0.945387985	0.273661286	Subclonal	likely_pathogenic	D	N	D	Passenger	FALSE	FALSE	FALSE
TCGA-D8-A1JN	9	131860413	G	T	CRAT	p.A448E	Missense_Mutation	FALSE	.	0.25	0.04	32	25	0.58	0.25829	0.927020342	0.30342302	Subclonal	likely_pathogenic	D	N	D	Passenger	FALSE	FALSE	FALSE
TCGA-D8-A1XC	16	28844531	C	T	ATXN2L	p.S604L	Missense_Mutation	FALSE	.	0.25	0	36	18	0.85	0.60195	0.987642881	0.43769724	Clonal	passenger	N	N	T	Passenger	FALSE	FALSE	FALSE
TCGA-D8-A1XC	2	128389268	C	T	MYO7B	p.P1704L	Missense_Mutation	FALSE	.	0.25	0	20	16	0.6	0.29964	0.9595162	0.261523152	Subclonal	passenger	N	D	D	Passenger	FALSE	FALSE	FALSE
TCGA-D8-A1XY	4	115586881	CAT	C	UGT8	p.I338Rfs*7	Frame_Shift_Del	FALSE	.	0.25	0	136	144	1	0.87484	1	0.797737499	Clonal	passenger	.	.	.	.	FALSE	FALSE	FALSE
TCGA-D8-A1XY	7	4189088	G	A	SDK1	p.V1540I	Missense_Mutation	FALSE	.	0.25	0	32	21	1	0.8921	1	0.554000526	Clonal	passenger	N	N	T	Passenger	FALSE	FALSE	FALSE
TCGA-E9-A54Y	3	99513184	G	A	COL8A1	p.G147R	Missense_Mutation	FALSE	.	0.25	0	36	30	1	0.82283	1	0.53691298	Clonal	likely_pathogenic	D	D	D	Passenger	FALSE	FALSE	FALSE
TCGA-E2-A155	12	90015552	T	C	ATP2B1	p.N454S	Missense_Mutation	FALSE	.	0.252293578	0	218	157	0.63	0.00009	0.77306523	0.488740956	Subclonal	likely_pathogenic	D	D	D	Passenger	FALSE	FALSE	FALSE
TCGA-E2-A15H	23	39923040	G	A	BCOR	p.S1223L	Missense_Mutation	FALSE	.	0.255506608	0.004048583	227	247	1	0.9341	1	0.823909539	Clonal	likely_pathogenic	D	N	T	Driver	TRUE	FALSE	TRUE
TCGA-E9-A1RB	1	196711113	A	G	CFH	p.Y1022C	Missense_Mutation	FALSE	loh	0.255555556	0	90	50	1	0.95679	1	0.795217725	Clonal	passenger	N	D	T	Passenger	FALSE	FALSE	FALSE
TCGA-E2-A15H	23	79990688	G	C	BRWD3	p.P308R	Missense_Mutation	FALSE	.	0.257575758	0	132	127	1	0.91403	1	0.759433867	Clonal	likely_pathogenic	D	D	T	Driver	FALSE	FALSE	FALSE
TCGA-AR-A0TW	12	9096396	C	A	M6PR	p.X151_splice	Splice_Site	FALSE	.	0.259259259	0	27	19	1	0.84553	1	0.495693411	Clonal	passenger	D	.	.	.	FALSE	FALSE	FALSE
TCGA-D8-A1XZ	9	88694123	C	T	GOLM1	p.R38Q	Missense_Mutation	FALSE	.	0.259259259	0	27	28	0.95	0.65561	1	0.424949391	Clonal	passenger	D	D	T	Passenger	FALSE	FALSE	FALSE
TCGA-E9-A1NI	20	31671213	A	AC	BPIFB4	p.V74Sfs*9	Frame_Shift_Ins	FALSE	.	0.259259259	0	27	21	1	0.83331	1	0.554338002	Clonal	passenger	D	.	.	.	FALSE	FALSE	FALSE
TCGA-E9-A1R0	3	57357142	G	C	DNAH12	p.L2558V	Missense_Mutation	FALSE	.	0.26	0	100	168	1	0.96385	1	0.816201248	Clonal	passenger	N	D	T	Passenger	FALSE	FALSE	FALSE
TCGA-A7-A4SC	3	17052496	CT	C	PLCL2	p.Y428Ifs*8	Frame_Shift_Del	FALSE	.	0.260869565	0	23	19	1	0.73876	1	0.440541736	Clonal	passenger	.	.	.	.	FALSE	FALSE	FALSE
TCGA-AR-A0TW	3	9974346	C	A	IL17RC	p.A552E	Missense_Mutation	FALSE	.	0.260869565	0	23	10	1	0.83804	1	0.462143739	Clonal	passenger	N	N	T	Passenger	FALSE	FALSE	FALSE
TCGA-BH-A1FM	10	101969382	T	G	CHUK	p.K366N	Missense_Mutation	FALSE	loh	0.260869565	0.017857143	23	56	0.98	0.73624	1	0.403379171	Clonal	passenger	D	N	T	Passenger	FALSE	FALSE	FALSE
TCGA-E9-A1R0	3	178936091	G	A	PIK3CA	p.E545K	Missense_Mutation	TRUE	.	0.261363636	0	88	89	1	0.96146	1	0.798378473	Clonal	likely_pathogenic	D	D	T	Driver	TRUE	TRUE	TRUE
TCGA-AR-A24O	8	133142164	G	A	KCNQ3	p.T655M	Missense_Mutation	FALSE	.	0.261904762	0	42	33	1	0.92621	1	0.667497755	Clonal	passenger	N	N	D	Passenger	FALSE	FALSE	FALSE
TCGA-D8-A1JN	19	4224160	TACAG	T	ANKRD24	p.Y1112*	Frame_Shift_Del	FALSE	.	0.261904762	0	42	20	0.61	0.22223	0.920669422	0.35111295	Subclonal	passenger	D	.	.	.	FALSE	FALSE	FALSE
TCGA-E9-A1R0	23	140270877	C	T	LDOC1	p.M110I	Missense_Mutation	FALSE	.	0.261904762	0	42	102	1	0.94593	1	0.659342804	Clonal	passenger	N	N	T	Passenger	FALSE	FALSE	FALSE
TCGA-A7-A4SC	1	181765969	G	T	CACNA1E	p.G2125V	Missense_Mutation	FALSE	.	0.263157895	0	38	32	1	0.89149	1	0.613188699	Clonal	likely_pathogenic	D	D	D	Passenger	FALSE	FALSE	FALSE
TCGA-E9-A1R0	12	48538878	G	A	PFKM	p.W757*	Nonsense_Mutation	FALSE	.	0.264367816	0	87	146	1	0.96017	1	0.799637777	Clonal	passenger	A	.	.	.	FALSE	FALSE	FALSE
TCGA-A7-A4SC	17	39346498	C	G	KRTAP9-1	p.H120Q	Missense_Mutation	FALSE	.	0.265306122	0	147	133	1	0.88951	1	0.784839947	Clonal	passenger	N	N	T	Passenger	FALSE	FALSE	FALSE
TCGA-E9-A54Y	5	36049459	T	TA	UGT3A2	p.L125Ffs*3	Frame_Shift_Ins	FALSE	.	0.266666667	0	30	38	1	0.83322	1	0.525453728	Clonal	passenger	D	.	.	.	FALSE	FALSE	FALSE
TCGA-A2-A0T6	6	28360727	C	T	ZSCAN12	p.A167T	Missense_Mutation	FALSE	.	0.267489712	0	243	258	1	0.88863	1	0.800494975	Clonal	passenger	N	N	T	Passenger	FALSE	FALSE	FALSE
TCGA-D8-A1XZ	9	17484352	TA	T	CNTLN	p.M1308*	Frame_Shift_Del	FALSE	.	0.269230769	0	52	82	0.98	0.73116	1	0.560413451	Clonal	passenger	D	.	.	.	FALSE	FALSE	FALSE
TCGA-AR-A0TW	5	175763760	C	T	SIMC1	p.R303C	Missense_Mutation	FALSE	.	0.269662921	0	178	76	1	0.92287	1	0.870296478	Clonal	passenger	D	D	T	Passenger	FALSE	FALSE	FALSE
TCGA-E2-A155	7	76943704	A	G	GSAP	p.V731A	Missense_Mutation	FALSE	.	0.270588235	0	170	157	0.67	0.00685	0.842257366	0.513725146	Subclonal	passenger	D	N	T	Passenger	FALSE	FALSE	FALSE
TCGA-BH-A1FM	1	94520718	C	A	ABCA4	p.D846Y	Missense_Mutation	FALSE	.	0.271186441	0	59	72	1	0.87109	1	0.660556635	Clonal	likely_pathogenic	D	D	D	Passenger	FALSE	FALSE	FALSE
TCGA-A7-A4SC	8	99101489	C	A	C8orf47	p.Q82K	Missense_Mutation	FALSE	.	0.272727273	0	22	21	1	0.7531	1	0.449392665	Clonal	passenger	N	N	T	Passenger	FALSE	FALSE	FALSE
TCGA-D8-A1XY	17	7847523	C	T	CNTROB	p.R510*	Nonsense_Mutation	FALSE	loh	0.272727273	0	33	31	1	0.8318	1	0.536562127	Clonal	likely_pathogenic	A	.	.	.	FALSE	FALSE	FALSE
TCGA-A2-A0T6	17	37880219	T	A	ERBB2	p.L755M	Missense_Mutation	TRUE	.	0.273381295	0	139	150	1	0.86165	1	0.746364782	Clonal	likely_pathogenic	D	N	T	Driver	TRUE	FALSE	TRUE
TCGA-A2-A0T6	17	37880220	T	G	ERBB2	p.L755W	Missense_Mutation	TRUE	.	0.273381295	0	139	150	1	0.86165	1	0.746364782	Clonal	likely_pathogenic	D	D	T	Driver	TRUE	FALSE	TRUE
TCGA-BH-A1FM	5	176885421	C	T	DBN1	p.A474T	Missense_Mutation	FALSE	.	0.273684211	0	95	145	1	0.91304	1	0.783000091	Clonal	passenger	N	N	T	Passenger	FALSE	FALSE	FALSE

TCGA-E9-A1RI	7	127963629	G	A	RBM28	p.T452M	Missense_Mutation	FALSE	.	0.273809524	0	84	78	1	0.83606	1	0.710829575	Clonal	passenger	D	D	T	Passenger	FALSE	FALSE	FALSE
TCGA-D8-A1XC	1	179063271	C	T	TOR3A	p.L288F	Missense_Mutation	FALSE	.	0.274509804	0	51	36	0.93	0.69557	1	0.543044455	Clonal	passenger	D	N	T	Passenger	FALSE	FALSE	FALSE
TCGA-E9-A1RI	3	178952085	A	T	PIK3CA	p.H1047L	Missense_Mutation	TRUE	.	0.274509804	0	51	36	1	0.79274	1	0.623103317	Clonal	likely_pathogenic	D	N	T	Driver	TRUE	TRUE	TRUE
TCGA-D8-A1JN	1	170508363	C	T	GORAB	p.P50L	Missense_Mutation	FALSE	.	0.275862069	0	58	30	0.92	0.80549	1	0.557875457	Clonal	passenger	D	D	T	Passenger	FALSE	FALSE	FALSE
TCGA-AR-A0TW	18	22805247	C	T	ZNF521	p.V879I	Missense_Mutation	FALSE	loh	0.276119403	0	134	72	1	0.91526	1	0.767111975	Clonal	passenger	D	N	T	Passenger	TRUE	FALSE	FALSE
TCGA-A8-A08J	16	23713958	C	T	ERN2	p.G355E	Missense_Mutation	FALSE	.	0.276190476	0.010869565	105	92	1	0.9563	1	0.820542812	Clonal	passenger	D	D	T	Passenger	FALSE	FALSE	FALSE
TCGA-BH-A1FM	23	30872292	C	T	TAB3	p.G497E	Missense_Mutation	FALSE	.	0.277777778	0	36	58	1	0.85188	1	0.572285556	Clonal	passenger	D	N	T	Passenger	FALSE	FALSE	FALSE
TCGA-D8-A1JN	1	158054238	G	A	KIRREL	p.G127R	Missense_Mutation	FALSE	.	0.27826087	0	115	27	0.93	0.83616	1	0.657577228	Clonal	likely_pathogenic	D	D	D	Passenger	FALSE	FALSE	FALSE
TCGA-A2-A0T6	6	37418099	G	A	CMTR1	p.D173N	Missense_Mutation	FALSE	.	0.278911565	0	147	242	1	0.87432	1	0.764328543	Clonal	passenger	D	N	.	Passenger	FALSE	FALSE	FALSE
TCGA-E2-A155	1	156907126	C	T	ARHGEF11	p.R1452H	Missense_Mutation	FALSE	.	0.279069767	0	43	46	0.69	0.38088	0.962495472	0.407566431	Subclonal	passenger	N	N	T	Passenger	FALSE	FALSE	FALSE
TCGA-AR-A0TW	3	9730630	C	T	MTMR14	p.R433*	Nonsense_Mutation	FALSE	.	0.28	0	25	25	1	0.86514	1	0.509993069	Clonal	passenger	A	.	.	.	FALSE	FALSE	FALSE
TCGA-AR-A24O	10	125514209	C	T	CPXM2	p.V663I	Missense_Mutation	FALSE	.	0.28	0	125	115	1	0.89134	1	0.866642761	Clonal	passenger	D	N	T	Passenger	FALSE	FALSE	FALSE
TCGA-D8-A1JN	19	47425022	G	C	ARHGAP35	p.E1030D	Missense_Mutation	FALSE	loh	0.28	0	25	15	0.65	0.47145	0.963931603	0.324122119	Subclonal	passenger	D	N	T	Passenger	FALSE	TRUE	TRUE
TCGA-AR-A0TW	10	8115853	T	TG	GATA3	p.S401Vfs*106	Frame_Shift_Ins	FALSE	.	0.28125	0	64	89	1	0.94556	1	0.736302365	Clonal	likely_pathogenic	D	.	.	.	TRUE	TRUE	TRUE
TCGA-E2-A155	12	114380175	C	T	RBM19	p.R564Q	Missense_Mutation	FALSE	.	0.282051282	0	39	59	0.7	0.42011	0.967095643	0.401672741	Subclonal	passenger	D	N	T	Passenger	FALSE	FALSE	FALSE
TCGA-E9-A1NI	10	8111496	T	TGGAG	GATA3	p.R330Efs*23	Frame_Shift_Ins	FALSE	.	0.282051282	0	39	72	1	0.71457	1	0.581510187	Clonal	likely_pathogenic	D	.	.	.	TRUE	TRUE	TRUE
TCGA-E9-A1R0	14	86088051	G	A	FLRT2	p.V65I	Missense_Mutation	FALSE	.	0.283018868	0	53	97	1	0.94332	1	0.731991474	Clonal	passenger	D	N	T	Passenger	FALSE	FALSE	FALSE
TCGA-A2-A0T6	23	131762942	T	C	HS6ST2	p.K416R	Missense_Mutation	FALSE	.	0.283950617	0	81	142	1	0.83482	1	0.687935497	Clonal	passenger	D	N	T	Passenger	FALSE	FALSE	FALSE
TCGA-A2-A3KD	3	185879390	A	G	DGKG	p.X759_splice	Splice_Site	FALSE	.	0.285714286	0	42	43	1	0.78787	1	0.594079245	Clonal	passenger	D	.	.	.	FALSE	FALSE	FALSE
TCGA-A7-A4SC	4	114267087	G	A	ANK2	p.R1427Q	Missense_Mutation	FALSE	.	0.285714286	0	49	39	1	0.83037	1	0.638437106	Clonal	likely_pathogenic	D	D	T	Driver	FALSE	FALSE	FALSE
TCGA-BH-A1FM	19	42753848	C	A	ERF	p.G139V	Missense_Mutation	FALSE	.	0.285714286	0	14	66	1	0.80375	1	0.373258594	Clonal	passenger	D	D	T	Passenger	FALSE	FALSE	FALSE
TCGA-D8-A1JN	12	53012235	G	A	KRT73	p.S25L	Missense_Mutation	FALSE	.	0.285714286	0	21	11	0.67	0.52441	0.969817207	0.311245263	Clonal	passenger	N	D	D	Passenger	FALSE	FALSE	FALSE
TCGA-D8-A1XZ	20	58519040	G	T	FAM217B	p.K14N	Missense_Mutation	FALSE	.	0.285714286	0	42	39	1	0.81399	1	0.61298339	Clonal	passenger	N	N	T	Passenger	FALSE	FALSE	FALSE
TCGA-D8-A1XZ	23	79278719	G	T	TBX22	p.M112I	Missense_Mutation	FALSE	error	0.285714286	0	63	62	1	0.89746	1	0.726403614	Clonal	likely_pathogenic	D	D	D	Passenger	FALSE	FALSE	FALSE
TCGA-E9-A1RI	22	18909913	C	CT	PRODH	p.S285Kfs*12	Frame_Shift_Ins	FALSE	.	0.285714286	0	28	22	1	0.83108	1	0.551209128	Clonal	likely_pathogenic	.	.	.	.	FALSE	FALSE	FALSE
TCGA-E9-A54Y	1	186086699	C	A	HMCN1	p.T3931N	Missense_Mutation	FALSE	loh	0.285714286	0	49	25	1	0.8868	1	0.726433471	Clonal	passenger	N	N	T	Passenger	FALSE	FALSE	FALSE
TCGA-E2-A15R	1	178490396	C	T	TEX35	p.R195W	Missense_Mutation	FALSE	.	0.288770053	0	561	372	0.97	0.86957	1	0.836834208	Clonal	passenger	N	D	T	Passenger	FALSE	FALSE	FALSE
TCGA-AR-A0TW	3	38596022	T	G	SCN5A	p.I1521L	Missense_Mutation	FALSE	.	0.289473684	0	38	35	1	0.89651	1	0.617191943	Clonal	passenger	N	N	D	Passenger	FALSE	FALSE	FALSE
TCGA-E9-A1QZ	6	119228579	AT	A	ASF1A	p.L140Wfs*54	Frame_Shift_Del	FALSE	loh	0.291666667	0	48	74	1	0.41238	1	0.714698573	Subclonal	likely_pathogenic	D	.	.	.	FALSE	FALSE	FALSE
TCGA-E9-A54Y	5	150920247	G	A	FAT2	p.R2974C	Missense_Mutation	FALSE	.	0.291666667	0	48	78	1	0.88515	1	0.663059872	Clonal	passenger	D	D	T	Passenger	FALSE	FALSE	FALSE
TCGA-D8-A1JN	1	149885119	C	T	SV2A	p.E92K	Missense_Mutation	FALSE	.	0.292134831	0	89	16	0.97	0.86112	1	0.654435878	Clonal	passenger	D	N	T	Passenger	FALSE	FALSE	FALSE
TCGA-E2-A15H	12	32778624	C	T	FGD4	p.R558*	Nonsense_Mutation	FALSE	.	0.292792793	0	222	152	1	0.86195	1	0.898411191	Clonal	passenger	D	.	.	.	FALSE	FALSE	FALSE
TCGA-A2-A0T6	6	42713328	C	A	TBCC	p.V162F	Missense_Mutation	FALSE	.	0.294117647	0	17	18	1	0.70926	1	0.402085122	Clonal	passenger	N	N	T	Passenger	FALSE	FALSE	FALSE
TCGA-AR-A0TW	3	49053694	C	T	DALRD3	p.R409H	Missense_Mutation	FALSE	.	0.294117647	0	51	37	1	0.9099	1	0.683933971	Clonal	passenger	D	D	T	Passenger	FALSE	FALSE	FALSE
TCGA-BH-A1FM	6	19838221	T	G	ID4	p.V79G	Missense_Mutation	FALSE	.	0.294117647	0	17	22	1	0.8266	1	0.428174695	Clonal	likely_pathogenic	D	D	D	Passenger	FALSE	FALSE	FALSE
TCGA-D8-A1XZ	18	59221741	C	T	CDH20	p.P740L	Missense_Mutation	FALSE	.	0.294117647	0	17	15	1	0.67811	1	0.389465968	Clonal	likely_pathogenic	D	D	T	Driver	FALSE	FALSE	FALSE
TCGA-E9-A1RB	1	200378052	T	C	ZNF281	p.H261R	Missense_Mutation	FALSE	loh	0.294736842	0	95	57	1	0.92559	1	0.837043709	Clonal	passenger	D	D	T	Passenger	FALSE	FALSE	FALSE
TCGA-A2-A0T6	15	67479838	C	T	SMAD3	p.A382V	Missense_Mutation	FALSE	.	0.294871795	0	78	179	1	0.84491	1	0.700972897	Clonal	likely_pathogenic	D	D	D	Passenger	TRUE	FALSE	FALSE
TCGA-D8-A1JN	11	67186395	G	T	CARNS1	p.G178V	Missense_Mutation	FALSE	.	0.296296296	0.071428571	27	14	0.69	0.5252	0.970173313	0.359226841	Clonal	passenger	D	N	T	Passenger	FALSE	FALSE	FALSE
TCGA-A2-A0T6	16	68867233	A	ATGAT	CDH1	p.S829*	Frame_Shift_Ins	FALSE	loh	0.296875	0	64	111	0.87	0.73281	0.989751607	0.565488491	Clonal	likely_pathogenic	.	.	.	.	TRUE	TRUE	TRUE
TCGA-D8-A1JN	1	226334330	C	G	ACBD3	p.R523T	Missense_Mutation	FALSE	.	0.298850575	0	87	35	0.99	0.87244	1	0.664889191	Clonal	passenger	D	D	T	Passenger	FALSE	FALSE	FALSE
TCGA-BH-A1FM	12	52284892	C	A	ANKRD33	p.Q388K	Missense_Mutation	FALSE	.	0.3	0	30	42	1	0.91421	1	0.593345446	Clonal	passenger	N	N	T	Passenger	FALSE	FALSE	FALSE
TCGA-D8-A1XZ	12	41418956	G	A	CNTN1	p.R843Q	Missense_Mutation	FALSE	.	0.3	0	20	37	1	0.70042	1	0.43107381	Clonal	passenger	D	N	T	Passenger	FALSE	FALSE	FALSE
TCGA-E9-A1R0	11	117982545	G	A	TMPRSS4	p.D225N	Missense_Mutation	FALSE	.	0.3	0	20	70	1	0.91183	1	0.527963157	Clonal	passenger	N	N	T	Passenger	FALSE	FALSE	FALSE
TCGA-E2-A15H	6	144070161	A	G	PHACTR2	p.E85G	Missense_Mutation	FALSE	loh	0.302752294	0.002570694	327	389	1	0.96623	1	0.854848772	Clonal	passenger	D	D	T	Passenger	FALSE	FALSE	FALSE
TCGA-BH-A1FM	9	139369436	C	T	SEC16A	p.D878N	Missense_Mutation	FALSE	.	0.303030303	0	66	32	1	0.4859	1	0.807647539	Subclonal	passenger	D	N	T	Passenger	FALSE	FALSE	FALSE
TCGA-A7-A4SC	22	39069234	GA	G	CBY1	p.K126Nfs*15	Frame_Shift_Del	FALSE	loh	0.303571429	0	56	92	1	0.85385	1	0.626640622	Clonal	likely_pathogenic	.	.	.	.	FALSE	FALSE	FALSE
TCGA-E9-A1RB	19	6694484	C	G	C3	p.G1038R	Missense_Mutation	FALSE	.	0.303571429	0	56	47	1	0.91785	1	0.712634539	Clonal	passenger	D	D	T	Passenger	FALSE	FALSE	FALSE
TCGA-D8-A1JN	14	44975125	C	T	FSCB	p.E356K	Missense_Mutation	FALSE	.	0.305555556	0	72	62	0.71	0.30368	0.944311823	0.485967197	Subclonal	passenger	N	N	T	Passenger	FALSE	FALSE	FALSE
TCGA-E9-A1NI	3	178936082	G	A	PIK3CA	p.E542K	Missense_Mutation	TRUE	.	0.305555556	0	36	72	1	0.74521	1	0.600771679	Clonal	likely_pathogenic	D	N	T	Driver	TRUE	TRUE	TRUE
TCGA-A8-A08J	17	7318874	C	A	NLGN2	p.P361H	Missense_Mutation	FALSE	.	0.307692308	0	39	32	1	0.91987	1	0.660883377	Clonal	likely_pathogenic	D	D	T	Driver	FALSE	FALSE	FALSE
TCGA-BH-A1FM	11	107375925	G	C	ALKBH8	p.A485G	Missense_Mutation	FALSE	.	0.307692308	0	26	32	1	0.91095	1	0.569853374	Clonal	passenger	D	D	T	Passenger	FALSE	FALSE	FALSE
TCGA-D8-A1JN	1	171527054	C	G	PRRC2C	p.Q1933E	Missense_Mutation	FALSE	.	0.307692308	0	143	52	1	0.91004	1	0.747581844	Clonal	passenger	N	N	T	Passenger	FALSE	FALSE	FALSE
TCGA-D8-A1XC	14	38060754	TGCTGCTCC	T	FOXA1	p.E410Afs*2	Frame_Shift_Del	FALSE	.	0.307692308	0	39	27	0.74	0.41494	0.974839667	0.435751142	Subclonal	likely_pathogenic	.	.	.	.	TRUE	TRUE	TRUE
TCGA-E9-A1R0	1	1163972	C	T	SDF4	p.E68K	Missense_Mutation	FALSE	.	0.307692308	0	13	34	1	0.88955	1	0.424994673	Clonal	passenger	D	N	T	Passenger	FALSE	FALSE	FALSE
TCGA-D8-A1JN	9	95086375	G	T	NOL8	p.D23E	Missense_Mutation	FALSE	.	0.309090909	0	110	64	0.72	0.1588	0.920437136	0.531113119	Subclonal	passenger	D	N	T	Passenger	FALSE	FALSE	FALSE
TCGA-D8-A1JN	1	150550932	C	T	MCL1	p.D242N	Missense_Mutation	FALSE	.	0.31	0	100	55	1	0.89632	1	0.705967968	Clonal	passenger	D	D	T	Passenger	FALSE	FALSE	FALSE
TCGA-AR-A0TW	14	37180577	G	C	SLC25A21	p.N183K	Missense_Mutation	FALSE	.	0.310160428	0	187	90	1	0.97321	1	0.90223194	Clonal	passenger	D	D	T	Passenger	FALSE	FALSE	FALSE
TCGA-D8-A1XY	15	100672248	C	T	ADAMTS17	p.G562E	Missense_Mutation	FALSE	.	0.310344828	0	29	43	1	0.91284	1	0.593921138	Clonal	likely_pathogenic	D	D	D	Passenger	FALSE	FALSE	FALSE
TCGA-BH-A1FM	4	175709965	A	C	GLRA3	p.X67_splice	Splice_Site	FALSE	.	0.311111111	0	90	70	1	0.9572	1	0.813866963	Clonal	passenger	D	.	.	.	FALSE	FALSE	FALSE
TCGA-D8-A1XZ	12	91502039	C	T	LUM	p.E240K	Missense_Mutation	FALSE	.	0.311594203	0	138	153	1	0.88631	1	0.785897898	Clonal	passenger	D	N	T	Passenger	FALSE	FALSE	FALSE
TCGA-BH-A1FM	11	87908404	A	T	RAB38	p.V50E	Missense_Mutation	FALSE	.	0.3125	0	16	40	1	0.87651	1	0.457406613	Clonal	passenger	D	D	T	Passenger	FALSE	FALSE	FALSE
TCGA-BH-A1FM	7	80418831	GCTC	G	SEMA3C	p.G381del	In_Frame_Del	FALSE	.	0.313253012	0.014492754	83	69	1	0.95746	1	0.803975039	Clonal	passenger	.	.	.	.	FALSE	FALSE	FALSE
TCGA-D8-A1XZ	5	26881405	G	A	CDH9	p.T737M	Missense_Mutation	FALSE	.	0.315315315	0	111	120	1	0.87478	1	0.763123971	Clonal	passenger	D	D	T	Passenger	FALSE	FALSE	FALSE
TCGA-BH-A1FM	16	84270709	G	A	KCNG4	p.A128V	Missense_Mutation	FALSE	.	0.315789474	0	19	38	1	0.89339	1	0.503758755	Clonal	passenger	D	N	T	Passenger	FALSE	FALSE	FALSE
TCGA-D8-A1XZ	16	49669753	C	T	ZNF423	p.V1104M	Missense_Mutation	FALSE	.	0.315789474	0	19	14	1	0.79257	1	0.479612117	Clonal	passenger	N	N	T	Passenger	FALSE	FALSE	FALSE
TCGA-D8-A1XZ	18	48255548	C	T	MAPK4	p.S363L	Missense_Mutation	FALSE	.	0.315789474	0.033333333	19	30	1	0.71753	1	0.442309213	Clonal	passenger	D	N	T	Passenger	FALSE	FALSE	FALSE
TCGA-E9-A1RB	20	41420080	C	T	PTPRT	p.G81R	Missense_Mutation	FALSE	.	0.315789474	0	57	34	1	0.95681	1	0.752078391	Clonal	passenger	D	N	T	Passenger	TRUE	FALSE	FALSE
TCGA-E2-A15H	9	114170924	C	A	KIAA0368	p.G1038V	Missense_Mutation	FALSE	.	0.317307692	0	208	172	1	0.95868	1	0.907689389	Clonal	passenger	D	D	T	Passenger	FALSE	FALSE	FALSE
TCGA-D8-A1JN	6	117997186	C	G	NUS1	p.A118G	Missense_Mutation	FALSE	.	0.32	0	25	16	0.74	0.62099	0.979135094	0.385748439	Clonal	passenger	D	D	T	Passenger	FALSE	FALSE	FALSE
TCGA-D8-A1XZ	13	31722195	G	A	HSPH1	p.P427S	Missense_Mutation	FALSE	.	0.322033898	0	59	85	1	0.83063	1	0.675635041	Clonal	passenger	D	D	T	Passenger	FALSE	FALSE	FALSE
TCGA-D8-A1XZ	2	141081533	A	G	LRP1B	p.V4148A	Missense_Mutation	FALSE	.	0.322033898	0	59	71	1	0.83063	1	0.675635041	Clonal	likely_pathogenic	D	N	D	Passenger	TRUE	FALSE	FALSE
TCGA-D8-A1JN	2	227661256	C	T	IRS1	p.M733I	Missense_Mutation	FALSE	.	0.322580645	0	31	26	0.75	0.61169	0.978465142	0.41957933	Clonal	passenger	D	N	T	Passenger	FALSE	FALSE	FALSE
TCGA-E9-A54Y	18	43667372	T	C	ATP5A1	p.M296V	Missense_Mutation	FALSE	.	0.322580645	0	31	37	1	0.8406	1	0.574768914	Clonal	passenger	D	D	T	Passenger	FALSE	FALSE	FALSE
TCGA-D8-A1JN	10	126523424	G	A	FAM175B	p.E378K	Missense_Mutation	FALSE	.	0.323529412	0	34	29	0.75	0.60584	0.978031279	0.433353796	Clonal	passenger	D	N	T	Passenger	FALSE	FALSE	FALSE
TCGA-D8-A1JN	1	158615335	C	T	SPTA1	p.E1316K	Missense_Mutation	FALSE	.	0.325396825	0	126	65	1	0.92379	1	0.763940685	Clonal	passenger	D	D	T	Passenger	FALSE	FALSE	FALSE
TCGA-D8-A1XC	23	70627446	G	A	TAF1	p.R1397Q	Missense_Mutation	FALSE	.	0.325581395	0	43	40	0.78	0.48846	0.98079275	0.4801188	Subclonal	passenger	D	D	T	Passenger	FALSE	TRUE	FALSE
TCGA-E9-A1R0	2	80874927	G	A	CTNNA2	p.R883Q	Missense_Mutation	FALSE	.	0.326086957	0	46	61	1	0.89648	1	0.744907213	Clonal	passenger	D	N	T	Passenger	FALSE	FALSE	FALSE
TCGA-D8-A1XZ	1	220791767	C	T	MARK1	p.A223V	Missense_Mutation	FALSE	.	0.327272727	0	55	102	0.87	0.71272	0.98927789	0.558612202	Clonal	likely_pathogenic	D	D	T	Driver	FALSE	FALSE	FALSE
TCGA-D8-A1XY	19	55246750	G	T	KIR3DL3	p.G327V	Missense_Mutation	FALSE	.	0.32739726	0	730	659	1	0.92682	1	0.971538848	Clonal	passenger	N	D	T	Passenger	FALSE	FALSE	FALSE
TCGA-D8-A1XC	19	9066614	C	A	MUC16	p.E6944D	Missense_Mutation	FALSE	.	0.328358209	0	67	59	0.79	0.44307	0.977721272	0.538668969	Subclonal	passenger	.	N	T	Passenger	FALSE	FALSE	FALSE
TCGA-A7-A4SC	8	141549448	G	A	AGO2	p.R714W	Missense_Mutation	FALSE	.	0.330097087	0	103	53	1	0.8057	1	0.819759367	Clonal	passenger	D	D	T	Passenger	FALSE	FALSE	FALSE
TCGA-A2-A0T6	6	131912472	G	A	MED23	p.Q1223*	Nonsense_Mutation	FALSE	loh	0.333333333	0	51	43	0.98	0.81645	1	0.600601861	Clonal	likely_pathogenic	D	.	.	.	FALSE	FALSE	TRUE
TCGA-A7-A4SC	1	151137721	G	A	LYSMD1	p.S5F	Missense_Mutation	FALSE	.	0.333333333	0	27	25	1	0.90517	1	0.620969202	Clonal	passenger	D	N	T	Passenger	FALSE	FALSE	FALSE
TCGA-BH-A1FM	16	84779261	G	T	USP10	p.V392L	Missense_Mutation	FALSE	.	0.333333333	0	15	22	1	0.8848	1	0.464886362	Clonal	passenger	D	N	T	Passenger	FALSE	FALSE	FALSE
TCGA-D8-A1JN	22	40658045	C	A	TNRC6B	p.Q109K	Missense_Mutation	FALSE	loh	0.333333333	0.047619048	9	21	0.44	0.0964	0.852349893	0.156122892	Subclonal	passenger	D	N	T	Passenger	FALSE	FALSE	FALSE
TCGA-D8-A1XZ	18	67794783	C	T	RTTN	p.C1113Y	Missense_Mutation	FALSE	.	0.333333333	0.023255814	42	43	1	0.81087	1	0.630597777	Clonal	passenger	D	D	T	Passenger	FALSE	FALSE	FALSE
TCGA-D8-A1XZ	7	38431512	G	A	AMPH	p.A572V	Missense_Mutation	FALSE	.	0.333333333	0	39	39	1	0.80419	1	0.616164467	Clonal	passenger	N	N	T	Passenger	FALSE	FALSE	FALSE
TCGA-D8-A1XZ	23	153171699	C	T	AVPR2	p.R247C	Missense_Mutation	FALSE	.	0.333333333	0	63	75	1	0.87751	1	0.752440608	Clonal	passenger	N	N	T	Passenger	FALSE	FALSE	FALSE
TCGA-E2-A15R	3	13860569	A	T	WNT7A	p.C308S	Missense_Mutation	FALSE	.	0.333333333	0	93	99	0.79	0.37697	0.971762564	0.575436862	Subclonal	likely_pathogenic	D	D	D	Driver	FALSE	FALSE	FALSE
TCGA-E9-A1RI	16	89178629	C	T	ACSF3	p.R318C	Missense_Mutation	FALSE	loh	0.333333333	0	27	47	1	0.80085	1	0.528828401	Clonal	passenger	D	D	T	Passenger	FALSE	FALSE	FALSE
TCGA-E9-A1RI	4	1019054	CCA	C	FGFRL1	p.H485Lfs*66	Frame_Shift_Del	FALSE	.	0.333333333	0	9	9	1	0.71871	1	0.321843917	Clonal	passenger	.	.	.	.	FALSE	FALSE	FALSE
TCGA-D8-A1JN	1	158576979	C	T	OR10Z1	p.H251Y	Missense_Mutation	FALSE	.	0.335714286	0	140	50	1	0.93511	1	0.793413747	Clonal	passenger	D	D	T	Passenger	FALSE	FALSE	FALSE
TCGA-D8-A1XY	21	47918525	C	G	DIP2A	p.S145C	Missense_Mutation	FALSE	.	0.338461538	0	65	51	1	0.95797	1	0.784391131	Clonal	passenger	D	D	T	Passenger	FALSE	FALSE	FALSE
TCGA-D8-A1XY	7	141755462	G	T	MGAM	p.R1140M	Missense_Mutation	FALSE	.	0.338582677	0	127	108	1	0.95534	1	0.884369248	Clonal	passenger	N	N	D	Passenger	FALSE	FALSE	FALSE
TCGA-BH-A1FM	1	236143802	C	T	NID1	p.D1127N	Missense_Mutation	FALSE	.	0.339285714	0	56	65	1	0.87879	1	0.786844493	Clonal	passenger	D	D	T	Passenger	FALSE	FALSE	FALSE
TCGA-BH-A1FM	23	53588774	G	A	HUWE1	p.R2484C	Missense_Mutation	FALSE	.	0.339805825	0	103	90	1	0.9682	1	0.853590214	Clonal	passenger	D	D	T	Passenger	FALSE	FALSE	FALSE
TCGA-E2-A15H	10	8115851	C	CA	GATA3	p.M400Nfs*107	Frame_Shift_Ins	FALSE	.	0.340101523	0.011695906	197	171	1	0.97672	1	0.914322501	Clonal	likely_pathogenic	D	.	.	.	TRUE	TRUE	TRUE
TCGA-D8-A1JN	5	94936613	G	C	ARSK	p.E387Q	Missense_Mutation	FALSE	.	0.340425532	0	141	69	0.79	0.3714	0.958519629	0.616630638	Subclonal	likely_pathogenic	D	N	D	Passenger	FALSE	FALSE	FALSE
TCGA-A8-A08J	17	66872768	C	A	ABCA8	p.X1385_splice	Splice_Site	FALSE	.	0.342105263	0	266	214	1	0.91208	1	0.942153879	Clonal	passenger	D	.	.	.	FALSE	FALSE	FALSE
TCGA-BH-A1FM	23	152807837	G	A	ATP2B3	p.E241K	Missense_Mutation	FALSE	.	0.342105263	0	38	39	1	0.94676	1	0.693058638	Clonal	likely_pathogenic	D	D	D	Passenger	TRUE	FALSE	FALSE
TCGA-E2-A155	8	56912024	G	A	LYN	p.G418R	Missense_Mutation	FALSE	.	0.342723005	0	426	361	0.85	0.20562	0.955494441	0.735972456	Subclonal	likely_pathogenic	D	D	D	Driver	FALSE	FALSE	FALSE
TCGA-BH-A1FM	7	75511159	T	G	RHBDD2	p.V64G	Missense_Mutation	FALSE	.	0.34375	0.020833333	32	48	1	0.94027	1	0.658218535	Clonal	passenger	D	D	T	Passenger	FALSE	FALSE	FALSE
TCGA-D8-A1JN	3	122667367	G	T	SEMA5B	p.T159N	Missense_Mutation	FALSE	.	0.346153846	0	26	18	0.81	0.69471	0.984771078	0.431337707	Clonal	passenger	N	N	T	Passenger	FALSE	FALSE	FALSE
TCGA-A8-A08J	7	100459398	C	A	SLC12A9	p.H526N	Missense_Mutation	FALSE	.	0.346938776	0	49	46	1	0.92383	1	0.760524625	Clonal	likely_pathogenic	D	D	D	Driver	FALSE	FALSE	FALSE
TCGA-E9-A1RB	7	75182834	G	T	HIP1	p.S738Y	Missense_Mutation	FALSE	.	0.346938776	0	49	27	1	0.95165	1	0.753143794	Clonal	passenger	N	N	T	Passenger	TRUE	FALSE	FALSE
TCGA-AR-A0TW	23	118603961	CT	C	SLC25A5	p.E151Kfs*65	Frame_Shift_Del	FALSE	loh	0.347826087	0.037037037	69	27	1	0.88527	1	0.765722684	Clonal	likely_pathogenic	D	.	.	.	FALSE	FALSE	FALSE
TCGA-E2-A15H	12	58159205	G	A	CYP27B1	p.A155V	Missense_Mutation	FALSE	.	0.347826087	0	46	39	1	0.95787	1	0.733239704	Clonal	passenger	N	N	T	Passenger	FALSE	FALSE	FALSE
TCGA-D8-A1JN	11	62398569	G	C	GANAB	p.I383M	Missense_Mutation	FALSE	.	0.35106383	0	94	59	0.82	0.61063	0.979138523	0.602384476	Clonal	likely_pathogenic	D	N	D	Passenger	FALSE	FALSE	FALSE
TCGA-D8-A1JN	13	25049681	C	T	PARP4	p.D615N	Missense_Mutation	FALSE	.	0.351351351	0	74	63	0.82	0.6511	0.981968164	0.57806122	Clonal	passenger	D	D	T	Passenger	FALSE	FALSE	FALSE
TCGA-E2-A155	3	97614915	C	T	CRYBG3	p.R615W	Missense_Mutation	FALSE	.	0.351428571	0	350	287	0.87	0.47416	0.976798544	0.74556129	Subclonal	likely_pathogenic	D	.	D	Passenger	FALSE	FALSE	FALSE
TCGA-D8-A1JN	7	100030928	G	C	MEPCE	p.E645Q	Missense_Mutation	FALSE	.	0.351851852	0	54	35	0.82	0.68498	0.984240627	0.54216814	Clonal	passenger	D	N	.	Passenger	FALSE	FALSE	FALSE
TCGA-E9-A1R0	2	27804427	G	A	C2orf16	p.R1663H	Missense_Mutation	FALSE	.	0.352112676	0	71	117	1	0.76474	1	0.834164478	Clonal	passenger	N	N	T	Passenger	FALSE	FALSE	FALSE
TCGA-E2-A155	2	216209566	ACT	A	ATIC	p.S432Cfs*29	Frame_Shift_Del	FALSE	.	0.352272727	0	176	182	0.87	0.65741	0.986795266	0.699663581	Clonal	likely_pathogenic	.	.	.	.	TRUE	FALSE	FALSE
TCGA-D8-A1JN	12	70989934	C	T	PTPRB	p.E385K	Missense_Mutation	FALSE	.	0.352380952	0	105	71	0.82	0.5987	0.978389927	0.615654077	Clonal	passenger	N	N	T	Passenger	TRUE	FALSE	FALSE
TCGA-E9-A1R0	6	117861948	G	A	DCBLD1	p.V407M	Missense_Mutation	FALSE	loh	0.352941176	0	34	72	1	0.95014	1	0.687386193	Clonal	likely_pathogenic	D	N	D	Passenger	FALSE	FALSE	FALSE
TCGA-BH-A1FM	9	139369265	C	G	SEC16A	p.E935Q	Missense_Mutation	FALSE	.	0.353982301	0	113	36	1	0.34932	1	0.898590288	Subclonal	passenger	N	N	T	Passenger	FALSE	FALSE	FALSE
TCGA-E2-A15R	1	1222976	G	A	SCNN1D	p.G467R	Missense_Mutation	FALSE	.	0.354166667	0	96	52	1	0.88164	1	0.774503409	Clonal	passenger	N	D	T	Passenger	FALSE	FALSE	FALSE
TCGA-A2-A3KD	23	70837002	G	A	CXCR3	p.P154L	Missense_Mutation	FALSE	.	0.357142857	0	56	51	1	0.8735	1	0.743777865	Clonal	passenger	D	D	T	Passenger	FALSE	FALSE	FALSE
TCGA-BH-A1FM	7	55914275	C	T	14-Sep	p.C37Y	Missense_Mutation	FALSE	.	0.357142857	0	70	83	1	0.96153	1	0.813951998	Clonal	passenger	D	N	T	Passenger	FALSE	FALSE	FALSE
TCGA-D8-A1XC	12	53825208	G	A	AMHR2	p.G558D	Missense_Mutation	FALSE	.	0.357142857	0	42	27	0.86	0.62047	0.988468494	0.531583698	Clonal	passenger	N	N	D	Passenger	FALSE	FALSE	FALSE
TCGA-D8-A1XZ	12	120287989	G	T	CIT	p.H169N	Missense_Mutation	FALSE	.	0.357142857	0.009090909	70	110	1	0.85975	1	0.751822289	Clonal	passenger	N	N	T	Passenger	FALSE	FALSE	FALSE
TCGA-D8-A1JN	19	48789311	G	C	ZNF114	p.D144H	Missense_Mutation	FALSE	loh	0.361111111	0	36	30	0.84	0.73152	0.987217948	0.503208758	Clonal	passenger	N	N	T	Passenger	FALSE	FALSE	FALSE
TCGA-A8-A08J	4	83826044	A	AGCT	THAP9	p.L80dup	In_Frame_Ins	FALSE	loh	0.361445783	0	83	111	1	0.90371	1	0.850581194	Clonal	passenger	N	N	.	.	FALSE	FALSE	FALSE
TCGA-BH-A1FM	9	107332253	G	C	OR13C8	p.D269H	Missense_Mutation	FALSE	loh	0.361702128	0	47	70	1	0.88091	1	0.694302627	Clonal	passenger	N	N	T	Passenger	FALSE	FALSE	FALSE
TCGA-E9-A1R0	16	67645418	T	C	CTCF	p.F228S	Missense_Mutation	FALSE	loh	0.361702128	0	47	70	1	0.95352	1	0.758569829	Clonal	passenger	D	D	T	Passenger	TRUE	TRUE	TRUE
TCGA-E2-A15H	12	106461239	G	A	NUAK1	p.P443S	Missense_Mutation	FALSE	.	0.3625	0	160	105	1	0.97727	1	0.907820014	Clonal	passenger	N	N	T	Passenger	FALSE	FALSE	FALSE
TCGA-E2-A155	2	32476278	G	A	NLRC4	p.Q219*	Nonsense_Mutation	FALSE	.	0.363636364	0	143	131	0.9	0.75696	1	0.704902345	Clonal	passenger	A	.	.	.	FALSE	FALSE	FALSE
TCGA-E2-A15R	2	70923448	A	G	ADD2	p.C135R	Missense_Mutation	FALSE	.	0.364806867	0	233	179	0.86	0.46905	0.980256869	0.717933765	Subclonal	passenger	D	D	T	Passenger	FALSE	FALSE	FALSE
TCGA-AR-A0TW	1	62979452	A	C	DOCK7	p.S1321A	Missense_Mutation	FALSE	.	0.365517241	0	145	89	1	0.97229	1	0.896315531	Clonal	passenger	D	N	T	Passenger	FALSE	FALSE	FALSE
TCGA-D8-A1JN	6	7578090	C	T	DSP	p.Q986*	Nonsense_Mutation	FALSE	.	0.366666667	0	60	40	0.85	0.7409	0.987823009	0.581863325	Clonal	likely_pathogenic	A	.	.	.	FALSE	FALSE	FALSE
TCGA-E2-A15H	14	52485857	C	T	NID2	p.V984M	Missense_Mutation	FALSE	.	0.368421053	0	76	53	1	0.96707	1	0.831082335	Clonal	likely_pathogenic	D	N	T	Driver	FALSE	FALSE	FALSE
TCGA-AR-A0TW	7	92732742	G	T	SAMD9	p.T890N	Missense_Mutation	FALSE	.	0.368852459	0.009090909	122	110	1	0.97132	1	0.881713921	Clonal	passenger	N	N	T	Passenger	FALSE	FALSE	FALSE
TCGA-D8-A1JN	12	6701207	T	A	CHD4	p.I989F	Missense_Mutation	FALSE	.	0.37037037	0	108	62	0.86	0.72681	0.987073554	0.654137863	Clonal	passenger	D	D	T	Passenger	TRUE	FALSE	TRUE
TCGA-AR-A0TW	11	43352111	C	G	API5	p.I425M	Missense_Mutation	FALSE	.	0.371428571	0	245	226	1	0.92335	1	0.942744366	Clonal	passenger	D	D	T	Passenger	FALSE	FALSE	FALSE
TCGA-E2-A155	1	158225973	C	T	CD1A	p.Q169*	Nonsense_Mutation	FALSE	.	0.371428571	0	175	210	0.92	0.7966	1	0.739275887	Clonal	passenger	A	.	.	.	FALSE	FALSE	FALSE
TCGA-D8-A1JN	5	38490331	G	A	LIFR	p.Q710*	Nonsense_Mutation	FALSE	.	0.373831776	0	107	98	0.87	0.74748	0.988259457	0.660180865	Clonal	likely_pathogenic	A	.	.	.	TRUE	TRUE	FALSE
TCGA-D8-A1JN	20	18429657	C	T	DZANK1	p.M200I	Missense_Mutation	FALSE	.	0.375	0	128	102	0.87	0.74154	0.987949968	0.679676971	Clonal	passenger	N	N	T	Passenger	FALSE	FALSE	FALSE
TCGA-D8-A1JN	3	62358482	G	A	FEZF2	p.S21L	Missense_Mutation	FALSE	.	0.375	0	8	11	0.87	0.7077	0.985666096	0.269360673	Clonal	passenger	D	N	T	Passenger	FALSE	FALSE	FALSE
TCGA-D8-A1XZ	20	47990860	C	T	KCNB1	p.V413I	Missense_Mutation	FALSE	.	0.375	0	32	59	1	0.90659	1	0.666455836	Clonal	likely_pathogenic	D	N	D	Passenger	FALSE	FALSE	FALSE
TCGA-BH-A1FM	5	154395548	C	T	KIF4B	p.A710V	Missense_Mutation	FALSE	.	0.375757576	0	165	92	1	0.96677	1	0.916581124	Clonal	passenger	D	N	T	Passenger	FALSE	FALSE	FALSE
TCGA-E2-A155	7	91603203	CT	C	AKAP9	p.V77*	Frame_Shift_Del	FALSE	.	0.375886525	0	141	119	0.93	0.81531	1	0.727821428	Clonal	passenger	.	.	.	.	FALSE	FALSE	FALSE
TCGA-E2-A155	3	142176483	T	C	ATR	p.M2540V	Missense_Mutation	FALSE	.	0.376569038	0	239	204	0.93	0.8228	1	0.775185276	Clonal	passenger	N	N	T	Passenger	TRUE	TRUE	FALSE
TCGA-D8-A1XC	2	179742731	G	A	CCDC141	p.T620I	Missense_Mutation	FALSE	.	0.378378378	0	37	49	0.91	0.68091	1	0.546910533	Clonal	passenger	N	N	T	Passenger	FALSE	FALSE	FALSE
TCGA-D8-A1JN	23	47307019	C	G	ZNF41	p.R717T	Missense_Mutation	FALSE	.	0.378787879	0	132	91	0.88	0.76258	0.989106193	0.690030136	Clonal	passenger	N	D	T	Passenger	FALSE	FALSE	FALSE
TCGA-D8-A1JN	1	43892848	C	T	SZT2	p.S1087L	Missense_Mutation	FALSE	.	0.379310345	0	87	55	0.88	0.78132	1	0.64866281	Clonal	passenger	N	D	.	Passenger	FALSE	FALSE	FALSE
TCGA-AR-A0TW	23	153668377	G	C	GDI1	p.G160R	Missense_Mutation	FALSE	.	0.38	0	50	27	1	0.95854	1	0.768685193	Clonal	passenger	D	D	T	Passenger	FALSE	FALSE	FALSE
TCGA-D8-A1JN	15	42743279	C	G	ZNF106	p.M374I	Missense_Mutation	FALSE	.	0.38028169	0	142	125	0.89	0.76694	0.9893415	0.69956718	Clonal	passenger	N	N	T	Passenger	FALSE	FALSE	FALSE
TCGA-A8-A08J	12	109637223	G	T	ACACB	p.G882C	Missense_Mutation	FALSE	.	0.380952381	0	147	81	1	0.3535	1	0.922080307	Clonal	likely_pathogenic	D	D	D	Passenger	FALSE	FALSE	FALSE
TCGA-A2-A0T6	5	34823772	A	T	RAI14	p.M612L	Missense_Mutation	FALSE	.	0.381578947	0	76	15	1	0.82419	1	0.803515039	Clonal	passenger	N	N	T	Passenger	FALSE	FALSE	FALSE
TCGA-E9-A1RB	5	115783352	C	G	SEMA6A	p.V684L	Missense_Mutation	FALSE	loh	0.382352941	0	34	33	1	0.91727	1	0.671375761	Clonal	passenger	N	N	T	Passenger	FALSE	FALSE	FALSE
TCGA-D8-A1JN	10	64573125	C	T	EGR2	p.E425K	Missense_Mutation	FALSE	.	0.382550336	0	149	74	0.89	0.7777	0.989903554	0.708211207	Clonal	passenger	D	N	T	Passenger	FALSE	FALSE	FALSE

TCGA-E2-A155	1	237863760	C	G	RYR2	p.D3120E	Missense_Mutation	FALSE	.	0.384146341	0	164	135	0.95	0.84902	1	0.757985599	Clonal	passenger	D	D	T	Passenger	FALSE	FALSE	FALSE
TCGA-A2-A3KD	19	47259734	G	C	FKRP	p.E343Q	Missense_Mutation	FALSE	.	0.384615385	0	13	9	1	0.8166	1	0.455379619	Clonal	likely_pathogenic	D	N	D	Passenger	FALSE	FALSE	FALSE
TCGA-D8-A1JN	1	112020326	C	G	C1orf162	p.L86V	Missense_Mutation	FALSE	.	0.384615385	0	39	29	0.9	0.78984	1	0.552596297	Clonal	passenger	N	N	T	Passenger	FALSE	FALSE	FALSE
TCGA-D8-A1XZ	1	197326056	C	A	CRB1	p.Q362K	Missense_Mutation	FALSE	.	0.384615385	0	91	140	1	0.87677	1	0.727667966	Clonal	likely_pathogenic	D	N	D	Passenger	FALSE	FALSE	FALSE
TCGA-BH-A1FM	12	56737619	C	A	STAT2	p.E801D	Missense_Mutation	FALSE	.	0.385321101	0.010869565	109	92	1	0.95723	1	0.884342961	Clonal	passenger	N	N	T	Passenger	FALSE	FALSE	FALSE
TCGA-D8-A1JN	19	52793484	C	T	ZNF766	p.S147L	Missense_Mutation	FALSE	loh	0.386666667	0	75	66	0.9	0.80768	1	0.644683546	Clonal	passenger	N	D	T	Passenger	FALSE	FALSE	FALSE
TCGA-D8-A1JN	10	96106314	C	A	NOC3L	p.X420_splice	Splice_Site	FALSE	.	0.387323944	0	142	103	0.9	0.80536	1	0.713162685	Clonal	passenger	D	.	.	.	FALSE	FALSE	FALSE
TCGA-D8-A1JN	3	122121674	G	C	FAM162A	p.L34F	Missense_Mutation	FALSE	.	0.387387387	0	111	76	0.9	0.8101	1	0.689710123	Clonal	passenger	N	N	T	Passenger	FALSE	FALSE	FALSE
TCGA-D8-A1XC	5	162891728	G	A	HMMR	p.X49_splice	Splice_Site	FALSE	.	0.388888889	0	36	68	0.93	0.70476	1	0.558716891	Clonal	passenger	D	.	.	.	FALSE	FALSE	FALSE
TCGA-D8-A1XZ	1	248685642	C	T	OR2G6	p.A232V	Missense_Mutation	FALSE	.	0.388888889	0	72	88	1	0.86873	1	0.702749864	Clonal	passenger	N	N	T	Passenger	FALSE	FALSE	FALSE
TCGA-D8-A1JN	11	58980260	C	A	MPEG1	p.D27Y	Missense_Mutation	FALSE	.	0.391304348	0	46	42	0.91	0.80898	1	0.587658339	Clonal	passenger	N	N	T	Passenger	FALSE	FALSE	FALSE
TCGA-E2-A15R	14	80669412	C	T	DIO2	p.E184K	Missense_Mutation	FALSE	.	0.391566265	0	166	156	0.93	0.76261	1	0.746227317	Clonal	likely_pathogenic	D	N	T	Driver	FALSE	FALSE	FALSE
TCGA-E9-A1RB	10	61959967	G	A	ANK3	p.H471Y	Missense_Mutation	FALSE	.	0.391891892	0	74	51	1	0.92415	1	0.847164587	Clonal	passenger	D	D	T	Passenger	FALSE	FALSE	FALSE
TCGA-D8-A1JN	19	49637900	C	T	PPFIA3	p.S461F	Missense_Mutation	FALSE	loh	0.392857143	0	56	28	0.91	0.81831	1	0.617876149	Clonal	passenger	D	D	T	Passenger	FALSE	FALSE	FALSE
TCGA-E2-A155	16	3486606	C	A	ZNF597	p.E365*	Nonsense_Mutation	FALSE	.	0.393442623	0	183	218	0.97	0.88008	1	0.784957601	Clonal	passenger	D	.	.	.	FALSE	FALSE	FALSE
TCGA-E9-A1NI	2	11355160	A	T	ROCK2	p.L581*	Nonsense_Mutation	FALSE	.	0.396551724	0	58	85	1	0.92091	1	0.789973788	Clonal	passenger	A	.	.	.	FALSE	FALSE	FALSE
TCGA-E2-A155	3	175042047	C	G	NAALADL2	p.S341R	Missense_Mutation	FALSE	.	0.397959184	0	392	323	0.99	0.91771	1	0.851977757	Clonal	passenger	N	N	T	Passenger	FALSE	FALSE	FALSE
TCGA-AR-A0TW	19	10081351	C	A	COL5A3	p.G1295C	Missense_Mutation	FALSE	.	0.4	0	5	11	1	0.80018	0.98931399	0.248168007	Clonal	likely_pathogenic	D	D	D	Passenger	FALSE	FALSE	TRUE
TCGA-D8-A1JN	22	21345981	G	A	LZTR1	p.G286R	Missense_Mutation	FALSE	loh	0.4	0	10	17	0.53	0.18153	0.897074881	0.216917529	Subclonal	passenger	D	D	T	Passenger	TRUE	FALSE	FALSE
TCGA-D8-A1XY	1	55625328	T	C	USP24	p.I342V	Missense_Mutation	FALSE	loh	0.4	0	70	114	1	0.9598	1	0.822614433	Clonal	passenger	D	N	T	Passenger	FALSE	FALSE	FALSE
TCGA-E9-A1NI	1	91182376	T	TG	BARHL2	p.Q126Pfs*20	Frame_Shift_Ins	FALSE	.	0.4	0	10	12	1	0.73657	1	0.408506306	Clonal	passenger	D	.	.	.	FALSE	FALSE	FALSE
TCGA-E9-A54Y	6	12123074	G	T	HIVEP1	p.A1016S	Missense_Mutation	FALSE	.	0.4	0	35	26	1	0.83356	1	0.739207643	Clonal	passenger	D	N	T	Passenger	FALSE	FALSE	FALSE
TCGA-E9-A54Y	8	27685656	A	G	PBK	p.F40L	Missense_Mutation	FALSE	loh	0.4	0	20	34	1	0.81328	1	0.523620065	Clonal	passenger	D	D	T	Passenger	FALSE	FALSE	FALSE
TCGA-E2-A155	10	8111501	G	GA	GATA3	p.R330Kfs*22	Frame_Shift_Ins	FALSE	.	0.402777778	0	216	204	1	0.90756	1	0.81570652	Clonal	likely_pathogenic	.	.	.	.	TRUE	TRUE	TRUE
TCGA-D8-A1JN	16	19477531	C	G	TMC5	p.T538S	Missense_Mutation	FALSE	.	0.403614458	0	166	98	0.94	0.86994	1	0.75812429	Clonal	passenger	N	N	T	Passenger	FALSE	FALSE	FALSE
TCGA-A8-A08J	1	169272390	A	C	NME7	p.F145V	Missense_Mutation	FALSE	.	0.403669725	0	109	121	1	0.65448	1	0.898219802	Clonal	passenger	D	D	T	Passenger	FALSE	FALSE	FALSE
TCGA-E9-A1R0	1	42049238	C	T	HIVEP3	p.A411T	Missense_Mutation	FALSE	.	0.404761905	0	42	124	1	0.62471	1	0.782733251	Clonal	passenger	D	N	T	Passenger	FALSE	FALSE	FALSE
TCGA-BH-A1FM	1	158669983	T	C	OR6K2	p.T154A	Missense_Mutation	FALSE	.	0.405063291	0	79	74	1	0.93835	1	0.858342408	Clonal	passenger	N	N	T	Passenger	FALSE	FALSE	FALSE
TCGA-E2-A155	4	5837643	G	A	CRMP1	p.S541L	Missense_Mutation	FALSE	.	0.405172414	0	116	119	1	0.8835	1	0.758581067	Clonal	likely_pathogenic	D	N	T	Driver	FALSE	FALSE	FALSE
TCGA-E2-A15R	17	56569882	G	A	MTMR4	p.H1134Y	Missense_Mutation	FALSE	.	0.405204461	0	269	282	0.96	0.84013	1	0.811141725	Clonal	passenger	D	D	T	Passenger	FALSE	FALSE	FALSE
TCGA-D8-A1XC	14	77767530	A	G	POMT2	p.L240S	Missense_Mutation	FALSE	.	0.407407407	0	27	29	0.98	0.71895	1	0.536012836	Clonal	likely_pathogenic	D	D	D	Passenger	FALSE	FALSE	FALSE
TCGA-E2-A155	17	76831512	G	A	USP36	p.R109*	Nonsense_Mutation	FALSE	loh	0.407407407	0	81	96	1	0.86744	1	0.718653126	Clonal	likely_pathogenic	D	.	.	.	FALSE	FALSE	FALSE
TCGA-A7-A4SC	22	36682850	C	T	MYH9	p.A1659T	Missense_Mutation	FALSE	loh	0.409090909	0	44	46	1	0.87385	1	0.722980645	Clonal	passenger	N	N	T	Passenger	TRUE	FALSE	FALSE
TCGA-E9-A1NI	17	694866	G	A	RNMTL1	p.V274M	Missense_Mutation	FALSE	loh	0.409090909	0	22	42	1	0.87871	1	0.628998334	Clonal	passenger	N	N	T	Passenger	FALSE	FALSE	FALSE
TCGA-E9-A54Y	7	134618585	G	T	CALD1	p.E355D	Missense_Mutation	FALSE	.	0.409090909	0	22	40	1	0.90343	1	0.599580767	Clonal	passenger	N	N	T	Passenger	FALSE	FALSE	FALSE
TCGA-D8-A1JN	16	4015811	C	T	ADCY9	p.E1343K	Missense_Mutation	FALSE	.	0.409836066	0	183	108	0.95	0.88996	1	0.777946339	Clonal	likely_pathogenic	D	N	D	Passenger	FALSE	FALSE	FALSE
TCGA-E2-A15R	5	133902109	G	A	JADE2	p.D425N	Missense_Mutation	FALSE	.	0.41025641	0	39	35	0.97	0.75161	1	0.598159302	Clonal	passenger	D	D	T	Passenger	FALSE	FALSE	FALSE
TCGA-E2-A155	18	28654837	A	G	DSC2	p.L567P	Missense_Mutation	FALSE	.	0.41031941	0	407	404	1	0.94308	1	0.875079765	Clonal	passenger	D	D	T	Passenger	FALSE	FALSE	FALSE
TCGA-E2-A15R	2	144193247	C	G	ARHGAP15	p.I184M	Missense_Mutation	FALSE	.	0.410526316	0	190	207	0.97	0.8479	1	0.792779646	Clonal	likely_pathogenic	D	N	D	Passenger	FALSE	FALSE	FALSE
TCGA-AR-A24O	1	55505552	A	ACTG	PCSK9	p.L23dup	In_Frame_Ins	FALSE	.	0.411764706	0.0625	17	16	1	0.69437	1	0.595567004	Clonal	passenger	.	.	.	.	FALSE	FALSE	FALSE
TCGA-D8-A1JN	20	32267771	G	GA	E2F1	p.S121Ffs*9	Frame_Shift_Ins	FALSE	.	0.411764706	0	51	30	0.96	0.85049	1	0.634581407	Clonal	passenger	D	.	.	.	FALSE	FALSE	FALSE
TCGA-E2-A15R	9	125288840	C	T	OR1N1	p.V245I	Missense_Mutation	FALSE	.	0.411764706	0	153	172	0.98	0.84151	1	0.775070714	Clonal	passenger	N	N	T	Passenger	FALSE	FALSE	FALSE
TCGA-E2-A155	4	170912804	C	T	MFAP3L	p.V319M	Missense_Mutation	FALSE	.	0.413612565	0	191	186	1	0.91977	1	0.822149782	Clonal	likely_pathogenic	D	N	D	Passenger	FALSE	FALSE	FALSE
TCGA-D8-A1JN	14	71058048	G	A	MED6	p.Q173*	Nonsense_Mutation	FALSE	.	0.413793103	0	87	70	0.96	0.87634	1	0.708703994	Clonal	passenger	D	.	.	.	FALSE	FALSE	FALSE
TCGA-E2-A155	5	172517553	C	G	CREBRF	p.S124C	Missense_Mutation	FALSE	.	0.414285714	0	210	174	1	0.92463	1	0.831639987	Clonal	likely_pathogenic	D	N	T	Driver	FALSE	FALSE	FALSE
TCGA-E2-A155	1	67206375	C	A	SGIP1	p.P757T	Missense_Mutation	FALSE	.	0.416030534	0.005952381	262	168	1	0.93541	1	0.85246402	Clonal	passenger	D	N	T	Passenger	FALSE	FALSE	FALSE
TCGA-D8-A1JN	16	8988916	C	T	USP7	p.G1004E	Missense_Mutation	FALSE	.	0.416666667	0	72	50	0.97	0.87358	1	0.689797485	Clonal	passenger	D	D	T	Passenger	FALSE	FALSE	FALSE
TCGA-D8-A1XY	3	178936091	G	A	PIK3CA	p.E545K	Missense_Mutation	TRUE	.	0.416666667	0	96	66	1	0.371	1	0.893826066	Subclonal	likely_pathogenic	D	D	T	Driver	TRUE	TRUE	TRUE
TCGA-E2-A15R	10	93021117	G	A	PCGF5	p.R170H	Missense_Mutation	FALSE	.	0.416666667	0	24	15	0.99	0.72296	1	0.522742101	Clonal	passenger	D	N	T	Passenger	FALSE	FALSE	FALSE
TCGA-E9-A1NI	2	227896715	G	A	COL4A4	p.P1255S	Missense_Mutation	FALSE	.	0.416666667	0	24	35	1	0.76365	1	0.604038119	Clonal	likely_pathogenic	D	N	D	Passenger	FALSE	FALSE	FALSE
TCGA-E9-A1NI	4	25395932	G	T	ANAPC4	p.E293*	Nonsense_Mutation	FALSE	.	0.417910448	0	67	60	1	0.93987	1	0.825873691	Clonal	passenger	A	.	.	.	FALSE	FALSE	FALSE
TCGA-D8-A1XC	11	112064318	C	T	BCO2	p.R139*	Nonsense_Mutation	FALSE	.	0.418181818	0	55	45	1	0.79638	1	0.668099927	Clonal	passenger	A	.	.	.	FALSE	FALSE	FALSE
TCGA-D8-A1JN	4	175899085	G	C	ADAM29	p.Q803H	Missense_Mutation	FALSE	.	0.419117647	0	136	57	0.98	0.90194	1	0.766247708	Clonal	passenger	N	N	T	Passenger	FALSE	FALSE	FALSE
TCGA-D8-A1JN	19	49912287	G	A	CCDC155	p.E353K	Missense_Mutation	FALSE	loh	0.420454545	0	88	50	0.98	0.88794	1	0.72074076	Clonal	passenger	N	N	T	Passenger	FALSE	FALSE	FALSE
TCGA-D8-A1XC	7	31912927	T	G	PDE1C	p.Y256S	Missense_Mutation	FALSE	.	0.421052632	0	38	29	1	0.7707	1	0.613961685	Clonal	passenger	D	D	T	Passenger	FALSE	FALSE	FALSE
TCGA-E2-A155	1	193045697	G	T	TROVE2	p.A290S	Missense_Mutation	FALSE	.	0.422222222	0	360	276	1	0.95222	1	0.884158477	Clonal	passenger	D	N	T	Passenger	FALSE	FALSE	FALSE
TCGA-E2-A155	20	52193283	A	G	ZNF217	p.C674R	Missense_Mutation	FALSE	.	0.422641509	0	265	207	1	0.94262	1	0.862663596	Clonal	passenger	N	N	T	Passenger	FALSE	FALSE	FALSE
TCGA-D8-A1XC	11	86267631	G	A	ME3	p.A144V	Missense_Mutation	FALSE	.	0.423076923	0	26	20	1	0.73969	1	0.549919833	Clonal	passenger	D	D	T	Passenger	FALSE	FALSE	FALSE
TCGA-E2-A15R	19	9407033	GT	G	ZNF699	p.H349Pfs*3	Frame_Shift_Del	FALSE	.	0.423076923	0	78	135	1	0.82733	1	0.717792811	Clonal	passenger	.	.	.	.	FALSE	FALSE	FALSE
TCGA-D8-A1JN	14	38061347	C	A	FOXA1	p.W214C	Missense_Mutation	FALSE	.	0.424242424	0	33	22	0.99	0.84453	1	0.583782906	Clonal	likely_pathogenic	D	D	D	Passenger	TRUE	TRUE	TRUE
TCGA-E2-A15R	3	62467428	G	A	CADPS	p.P1048L	Missense_Mutation	FALSE	.	0.424242424	0	264	295	1	0.90031	1	0.842388596	Clonal	passenger	D	N	T	Passenger	FALSE	FALSE	FALSE
TCGA-D8-A1JN	14	47389259	C	A	MDGA2	p.D434Y	Missense_Mutation	FALSE	.	0.424657534	0	146	67	0.99	0.91361	1	0.782303217	Clonal	passenger	D	N	T	Passenger	FALSE	FALSE	FALSE
TCGA-D8-A1XZ	13	60348934	C	T	DIAPH3	p.D1063N	Missense_Mutation	FALSE	.	0.425	0	80	91	1	0.77471	1	0.83367458	Clonal	likely_pathogenic	D	D	D	Passenger	FALSE	FALSE	FALSE
TCGA-D8-A1JN	15	64010840	C	T	HERC1	p.R1304Q	Missense_Mutation	FALSE	.	0.426356589	0	129	105	0.99	0.91171	1	0.772557633	Clonal	passenger	D	N	T	Passenger	FALSE	FALSE	FALSE
TCGA-E2-A15R	9	107457129	G	T	OR13D1	p.E143*	Nonsense_Mutation	FALSE	.	0.42816092	0	348	374	1	0.92041	1	0.868287634	Clonal	passenger	D	.	.	.	FALSE	FALSE	FALSE
TCGA-BH-A1FM	7	79082570	T	C	MAGI2	p.N23D	Missense_Mutation	FALSE	.	0.428571429	0.028571429	7	35	1	0.84898	1	0.357465835	Clonal	passenger	D	N	T	Passenger	FALSE	FALSE	FALSE
TCGA-D8-A1JN	19	9677557	G	A	ZNF121	p.H78Y	Missense_Mutation	FALSE	.	0.428571429	0	119	70	1	0.91176	1	0.767535718	Clonal	passenger	N	N	T	Passenger	FALSE	FALSE	FALSE
TCGA-D8-A1JN	19	39207792	G	A	ACTN4	p.E327K	Missense_Mutation	FALSE	loh	0.428571429	0	70	22	1	0.88919	1	0.704008077	Clonal	passenger	D	N	T	Passenger	FALSE	FALSE	FALSE
TCGA-D8-A1JN	5	122714068	C	A	CEP120	p.E720*	Nonsense_Mutation	FALSE	.	0.428571429	0	119	83	1	0.91176	1	0.767535718	Clonal	passenger	D	.	.	.	FALSE	FALSE	FALSE
TCGA-D8-A1JN	6	13977702	C	G	RNF182	p.L118V	Missense_Mutation	FALSE	.	0.428571429	0	56	38	1	0.87836	1	0.673133597	Clonal	likely_pathogenic	D	N	D	Passenger	FALSE	FALSE	FALSE
TCGA-D8-A1XC	15	100252709	CCAG	C	MEF2A	p.Q422del	In_Frame_Del	FALSE	.	0.428571429	0.05	21	20	1	0.72676	1	0.516075964	Clonal	passenger	.	.	.	.	FALSE	FALSE	FALSE
TCGA-E2-A155	19	58629723	G	A	ZSCAN18	p.A14V	Missense_Mutation	FALSE	.	0.428571429	0	7	13	1	0.70268	0.988851298	0.298561522	Clonal	passenger	N	.	.	Passenger	FALSE	FALSE	FALSE
TCGA-E2-A155	3	185990120	G	C	DGKG	p.T308S	Missense_Mutation	FALSE	.	0.428571429	0	273	287	1	0.94821	1	0.872693089	Clonal	likely_pathogenic	D	D	D	Passenger	FALSE	FALSE	FALSE
TCGA-E9-A54Y	20	48552965	C	G	RNF114	p.R6G	Missense_Mutation	FALSE	.	0.428571429	0	7	8	1	0.834	1	0.359365389	Clonal	passenger	N	N	T	Passenger	FALSE	FALSE	FALSE
TCGA-AR-A0TW	12	129373285	G	T	GLT1D1	p.A107S	Missense_Mutation	FALSE	.	0.429752066	0	121	41	1	0.47633	1	0.916168025	Clonal	passenger	D	N	T	Passenger	FALSE	FALSE	FALSE
TCGA-AR-A0TW	1	75683550	C	T	SLC44A5	p.X542_splice	Splice_Site	FALSE	.	0.429824561	0	114	124	1	0.95569	1	0.901404114	Clonal	passenger	D	.	.	.	FALSE	FALSE	FALSE
TCGA-D8-A1JN	19	23928047	C	T	ZNF681	p.R102K	Missense_Mutation	FALSE	loh	0.432692308	0	104	52	1	0.91162	1	0.759002089	Clonal	passenger	N	N	T	Passenger	FALSE	FALSE	FALSE
TCGA-D8-A1JN	2	242611609	C	T	ATG4B	p.S371F	Missense_Mutation	FALSE	.	0.432989691	0	97	61	1	0.90908	1	0.75136807	Clonal	passenger	D	N	T	Passenger	FALSE	FALSE	FALSE
TCGA-D8-A1XZ	10	31809597	A	G	ZEB1	p.N446S	Missense_Mutation	FALSE	.	0.433333333	0	30	50	1	0.84881	1	0.616842656	Clonal	passenger	N	N	T	Passenger	FALSE	FALSE	FALSE
TCGA-E2-A155	19	14015697	C	A	C19orf57	p.K3N	Missense_Mutation	FALSE	.	0.433510638	0	376	307	1	0.95969	1	0.901086651	Clonal	passenger	N	N	T	Passenger	FALSE	FALSE	FALSE
TCGA-D8-A1JN	12	6715439	C	T	CHD4	p.X34_splice	Splice_Site	FALSE	.	0.434782609	0	23	12	1	0.83323	1	0.532675007	Clonal	likely_pathogenic	D	.	.	.	TRUE	FALSE	TRUE
TCGA-D8-A1XZ	3	123038594	C	T	ADCY5	p.S728N	Missense_Mutation	FALSE	.	0.434782609	0	46	63	1	0.88314	1	0.758819083	Clonal	likely_pathogenic	D	D	D	Passenger	FALSE	FALSE	FALSE
TCGA-E9-A54Y	7	101833121	C	T	CUX1	p.A360V	Missense_Mutation	FALSE	.	0.434782609	0	23	20	1	0.92465	1	0.633463346	Clonal	likely_pathogenic	D	N	T	Driver	TRUE	FALSE	TRUE
TCGA-AR-A0TW	1	76276450	GA	G	MSH4	p.N220Ifs*8	Frame_Shift_Del	FALSE	.	0.435294118	0	170	118	1	0.94572	1	0.932377865	Clonal	passenger	D	.	.	.	FALSE	FALSE	FALSE
TCGA-E2-A15H	2	73303155	T	TG	RAB11FIP5	p.Q575Pfs*30	Frame_Shift_Ins	FALSE	.	0.435897436	0	78	83	1	0.97311	1	0.858188116	Clonal	passenger	D	.	.	.	FALSE	FALSE	FALSE
TCGA-BH-A1FM	23	27997704	G	T	DCAF8L1	p.T583N	Missense_Mutation	FALSE	.	0.4375	0.016129032	48	62	1	0.96036	1	0.794249412	Clonal	passenger	N	N	T	Passenger	FALSE	FALSE	FALSE
TCGA-D8-A1JN	23	70516865	G	A	NONO	p.R304H	Missense_Mutation	FALSE	.	0.4375	0	48	40	1	0.88023	1	0.662744953	Clonal	passenger	D	N	T	Passenger	TRUE	FALSE	FALSE
TCGA-E2-A155	15	24921813	G	T	NPAP1	p.V267F	Missense_Mutation	FALSE	.	0.4375	0	16	20	1	0.77985	1	0.47878877	Clonal	passenger	D	N	T	Passenger	FALSE	FALSE	FALSE
TCGA-E2-A15H	22	41310322	A	G	XPNPEP3	p.N350S	Missense_Mutation	FALSE	loh	0.438423645	0	203	259	1	0.00183	1	0.926360226	Clonal	passenger	D	N	T	Passenger	FALSE	FALSE	FALSE
TCGA-D8-A1JN	1	34006792	C	A	CSMD2	p.R2988I	Missense_Mutation	FALSE	.	0.43877551	0	98	54	1	0.91591	1	0.760999568	Clonal	passenger	D	D	T	Passenger	FALSE	FALSE	FALSE
TCGA-D8-A1XC	20	20036596	T	A	CRNKL1	p.K21N	Missense_Mutation	FALSE	.	0.43902439	0	41	53	1	0.80339	1	0.650782274	Clonal	passenger	N	N	T	Passenger	FALSE	FALSE	FALSE
TCGA-D8-A1JN	12	113599116	C	T	DDX54	p.R791Q	Missense_Mutation	FALSE	.	0.439189189	0	148	50	1	0.93237	1	0.805620646	Clonal	passenger	N	N	T	Passenger	FALSE	FALSE	FALSE
TCGA-D8-A1JN	19	9086981	C	T	MUC16	p.D1612N	Missense_Mutation	FALSE	.	0.44	0	175	137	1	0.93895	1	0.822646035	Clonal	passenger	.	N	T	Passenger	FALSE	FALSE	FALSE
TCGA-BH-A1FM	9	139368956	C	G	SEC16A	p.D1038H	Missense_Mutation	FALSE	.	0.441176471	0	34	20	1	0.30628	1	0.774777587	Subclonal	passenger	D	N	T	Passenger	FALSE	FALSE	FALSE
TCGA-E2-A155	15	102358597	G	A	OR4F15	p.D70N	Missense_Mutation	FALSE	.	0.441176471	0	374	363	1	0.96047	1	0.908871582	Clonal	passenger	D	D	T	Passenger	FALSE	FALSE	FALSE
TCGA-D8-A1JN	13	99460049	C	G	DOCK9	p.D1808H	Missense_Mutation	FALSE	.	0.441747573	0	206	115	1	0.94579	1	0.839659054	Clonal	passenger	D	D	T	Passenger	FALSE	FALSE	FALSE
TCGA-A2-A0T6	5	154317530	C	T	GEMIN5	p.R55Q	Missense_Mutation	FALSE	.	0.444444444	0	18	26	1	0.88959	1	0.583330447	Clonal	passenger	D	N	T	Passenger	FALSE	FALSE	FALSE
TCGA-D8-A1JN	3	137886069	G	C	DBR1	p.L190V	Missense_Mutation	FALSE	.	0.444444444	0	54	54	1	0.89327	1	0.690095677	Clonal	passenger	D	D	T	Passenger	FALSE	FALSE	FALSE
TCGA-D8-A1JN	7	150706129	G	A	NOS3	p.E742K	Missense_Mutation	FALSE	.	0.444444444	0	18	13	1	0.82505	1	0.496542179	Clonal	passenger	D	N	T	Passenger	FALSE	FALSE	FALSE
TCGA-D8-A1XZ	1	108507314	TCTC	T	VAV3	p.E59del	In_Frame_Del	FALSE	.	0.444444444	0	18	30	1	0.85865	1	0.574723042	Clonal	passenger	D	.	.	.	FALSE	FALSE	FALSE
TCGA-E9-A54Y	6	40400150	AG	A	LRFN2	p.L235Cfs*89	Frame_Shift_Del	FALSE	.	0.444444444	0	45	47	1	0.63043	1	0.809106187	Clonal	passenger	D	.	.	.	FALSE	FALSE	FALSE
TCGA-E2-A15H	23	129147755	C	T	BCORL1	p.P336L	Missense_Mutation	FALSE	.	0.445205479	0	146	147	1	0.97967	1	0.920682724	Clonal	passenger	D	N	T	Passenger	TRUE	FALSE	FALSE
TCGA-D8-A1JN	2	70486522	C	T	PCYOX1	p.S48L	Missense_Mutation	FALSE	.	0.445378151	0.01	119	100	1	0.93003	1	0.791978715	Clonal	passenger	D	D	T	Passenger	FALSE	FALSE	FALSE
TCGA-E2-A155	17	27380585	G	A	PIPOX	p.R211H	Missense_Mutation	FALSE	.	0.446808511	0	94	103	1	0.916	1	0.79114147	Clonal	passenger	N	N	D	Passenger	FALSE	FALSE	FALSE
TCGA-D8-A1JN	21	42754408	G	A	MX2	p.E217K	Missense_Mutation	FALSE	.	0.447368421	0	38	31	1	0.87631	1	0.638408008	Clonal	passenger	N	N	D	Passenger	FALSE	FALSE	FALSE
TCGA-E2-A15H	17	77768957	C	T	CBX8	p.G216D	Missense_Mutation	FALSE	.	0.448275862	0.023809524	116	42	1	0.50591	1	0.91560605	Clonal	passenger	N	N	T	Passenger	FALSE	FALSE	FALSE
TCGA-D8-A1JN	23	123514646	C	T	TENM1	p.E2647K	Missense_Mutation	FALSE	.	0.45	0	120	77	1	0.93411	1	0.799215988	Clonal	likely_pathogenic	D	D	D	Passenger	FALSE	TRUE	TRUE
TCGA-D8-A1JN	12	69656307	C	T	CPSF6	p.R542C	Missense_Mutation	FALSE	.	0.450549451	0	91	58	1	0.9234	1	0.768316689	Clonal	passenger	D	D	T	Passenger	FALSE	FALSE	FALSE
TCGA-D8-A1JN	14	50862466	C	T	CDKL1	p.D42N	Missense_Mutation	FALSE	.	0.450549451	0	91	78	1	0.9234	1	0.768316689	Clonal	passenger	D	D	T	Passenger	FALSE	FALSE	FALSE
TCGA-D8-A1JN	4	102839158	T	G	BANK1	p.F340V	Missense_Mutation	FALSE	.	0.452380952	0	84	63	1	0.92135	1	0.760930565	Clonal	passenger	N	N	T	Passenger	FALSE	FALSE	FALSE
TCGA-E2-A155	15	43122137	G	A	TTBK2	p.P144L	Missense_Mutation	FALSE	.	0.452914798	0	223	235	1	0.94945	1	0.883773399	Clonal	likely_pathogenic	D	N	T	Driver	FALSE	FALSE	FALSE
TCGA-BH-A1FM	17	12897755	C	G	ELAC2	p.E699Q	Missense_Mutation	FALSE	loh	0.453333333	0.011695906	75	171	1	0.93685	1	0.846181892	Clonal	passenger	D	N	T	Passenger	FALSE	FALSE	FALSE
TCGA-D8-A1JN	2	216284067	C	T	FN1	p.G573R	Missense_Mutation	FALSE	.	0.454545455	0	110	69	1	0.93406	1	0.795413358	Clonal	likely_pathogenic	D	D	T	Driver	FALSE	FALSE	FALSE
TCGA-D8-A1JN	6	43112230	G	A	PTK7	p.E773K	Missense_Mutation	FALSE	.	0.454545455	0	55	46	1	0.90276	1	0.705879042	Clonal	passenger	D	N	T	Passenger	FALSE	FALSE	FALSE
TCGA-D8-A1JN	2	138000073	C	T	THSD7B	p.P733S	Missense_Mutation	FALSE	.	0.455555556	0	180	73	1	0.95158	1	0.845833185	Clonal	passenger	D	D	T	Passenger	FALSE	FALSE	FALSE
TCGA-D8-A1JN	7	92120666	C	A	PEX1	p.E1120*	Nonsense_Mutation	FALSE	.	0.45631068	0	103	94	1	0.93269	1	0.790467454	Clonal	passenger	D	.	.	.	FALSE	FALSE	FALSE
TCGA-D8-A1JN	10	8115717	A	ACTATG	GATA3	p.K357Lfs*2	Frame_Shift_Ins	FALSE	.	0.456521739	0	46	35	1	0.89471	1	0.681184696	Clonal	likely_pathogenic	D	.	.	.	TRUE	TRUE	TRUE
TCGA-D8-A1XY	17	12032481	C	G	MAP2K4	p.P306R	Missense_Mutation	FALSE	loh	0.459459459	0	37	48	1	0.95538	1	0.760668203	Clonal	likely_pathogenic	D	D	T	Driver	TRUE	TRUE	TRUE
TCGA-D8-A1XZ	4	89671660	G	A	FAM13A	p.R619W	Missense_Mutation	FALSE	.	0.461538462	0	65	92	1	0.90928	1	0.830381031	Clonal	passenger	D	D	T	Passenger	FALSE	FALSE	FALSE
TCGA-D8-A1XC	19	31768399	G	A	TSHZ3	p.P767L	Missense_Mutation	FALSE	.	0.465116279	0	43	28	1	0.83612	1	0.690805267	Clonal	passenger	D	N	T	Passenger	FALSE	TRUE	FALSE
TCGA-D8-A1JN	12	94972209	C	G	TMCC3	p.K364N	Missense_Mutation	FALSE	.	0.465517241	0	58	50	1	0.91311	1	0.727399447	Clonal	passenger	D	D	T	Passenger	FALSE	FALSE	FALSE
TCGA-E2-A155	1	204214761	C	T	PLEKHA6	p.D672N	Missense_Mutation	FALSE	.	0.467391304	0	92	88	1	0.92327	1	0.811220141	Clonal	passenger	D	N	T	Passenger	FALSE	FALSE	FALSE
TCGA-E2-A155	2	200137283	C	T	SATB2	p.R618H	Missense_Mutation	FALSE	.	0.469565217	0.006802721	115	147	1	0.93149	1	0.838790788	Clonal	likely_pathogenic	D	N	D	Passenger	FALSE	FALSE	FALSE
TCGA-D8-A1JN	11	62996883	G	C	SLC22A25	p.S81*	Nonsense_Mutation	FALSE	.	0.471014493	0	138	79	1	0.95039	1	0.839151024	Clonal	passenger	N	.	.	.	FALSE	FALSE	FALSE
TCGA-D8-A1JN	11	58207576	C	T	OR5B12	p.D17N	Missense_Mutation	FALSE	.	0.473684211	0	38	50	1	0.89543	1	0.670682679	Clonal	passenger	N	N	T	Passenger	FALSE	FALSE	FALSE
TCGA-E9-A1RB	18	72173106	C	T	CNDP2	p.P76L	Missense_Mutation	FALSE	.	0.475	0	40	30	1	0.92226	1	0.791452147	Clonal	passenger	D	D	T	Passenger	FALSE	FALSE	FALSE
TCGA-D8-A1JN	19	14910637	G	GA	OR7C1	p.T105Hfs*17	Frame_Shift_Ins	FALSE	.	0.475609756	0.014285714	82	70	1	0.93398	1	0.785989527	Clonal	passenger	D	.	.	.	FALSE	FALSE	FALSE
TCGA-E2-A15H	6	149862525	G	A	PPIL4	p.R61C	Missense_Mutation	FALSE	loh	0.475862069	0	145	200	1	0.00064	1	0.916663284	Clonal	passenger	D	D	T	Passenger	FALSE	FALSE	FALSE
TCGA-E2-A155	16	31092215	C	T	ZNF646	p.Q1524*	Nonsense_Mutation	FALSE	.	0.476923077	0	65	87	1	0.90983	1	0.775677409	Clonal	passenger	D	.	.	.	FALSE	FALSE	FALSE
TCGA-E2-A15H	17	77768618	C	T	CBX8	p.G329E	Missense_Mutation	FALSE	.	0.478873239	0	71	29	1	0.47046	1	0.880117054	Subclonal	passenger	D	N	T	Passenger	FALSE	FALSE	FALSE
TCGA-E2-A155	1	149939428	C	A	OTUD7B	p.G98V	Missense_Mutation	FALSE	.	0.482142857	0	56	65	1	0.90362	1	0.759675656	Clonal	passenger	D	D	T	Passenger	FALSE	FALSE	FALSE
TCGA-D8-A1JN	12	57626352	G	A	SHMT2	p.M237I	Missense_Mutation	FALSE	.	0.483870968	0	31	24	1	0.88906	1	0.646350728	Clonal	passenger	D	N	T	Passenger	FALSE	FALSE	FALSE
TCGA-D8-A1JN	16	24166032	G	A	PRKCB	p.D365N	Missense_Mutation	FALSE	.	0.484848485	0	99	54	1	0.94389	1	0.818589824	Clonal	passenger	D	D	T	Passenger	FALSE	FALSE	FALSE
TCGA-E2-A15R	6	33374648	C	T	KIFC1	p.S658F	Missense_Mutation	FALSE	.	0.485875706	0	177	180	1	0.92842	1	0.881824548	Clonal	passenger	D	D	T	Passenger	FALSE	FALSE	FALSE
TCGA-D8-A1JN	23	63412446	G	C	AMER1	p.P241A	Missense_Mutation	FALSE	.	0.488888889	0	45	52	1	0.91244	1	0.715514345	Clonal	passenger	N	N	T	Passenger	TRUE	FALSE	FALSE
TCGA-D8-A1JN	2	179499240	C	G	TTN	p.D14090H	Missense_Mutation	FALSE	.	0.490909091	0	55	29	1	0.92291	1	0.748559046	Clonal	passenger	D	D	T	Passenger	FALSE	FALSE	FALSE
TCGA-D8-A1JN	8	70981503	G	A	PRDM14	p.T198M	Missense_Mutation	FALSE	.	0.491803279	0	61	40	1	0.92776	1	0.764278884	Clonal	passenger	D	D	T	Passenger	FALSE	FALSE	FALSE
TCGA-D8-A1JN	1	11345847	G	A	UBIAD1	p.E226K	Missense_Mutation	FALSE	.	0.49382716	0	81	44	1	0.9391	1	0.803776547	Clonal	likely_pathogenic	D	D	D	Passenger	FALSE	FALSE	FALSE
TCGA-D8-A1XZ	1	24857756	C	T	RCAN3	p.Q82*	Nonsense_Mutation	FALSE	.	0.494117647	0	85	53	1	0.29525	1	0.901872466	Clonal	passenger	D	.	.	.	FALSE	FALSE	FALSE
TCGA-E9-A1NI	1	169564091	A	G	SELP	p.V709A	Missense_Mutation	FALSE	.	0.495412844	0	109	91	1	0.31127	1	0.921460425	Clonal	passenger	N	N	T	Passenger	FALSE	FALSE	TRUE
TCGA-D8-A1JN	11	59559682	G	A	STX3	p.E154K	Missense_Mutation	FALSE	.	0.5	0	56	39	1	0.92648	1	0.760658648	Clonal	passenger	D	D	T	Passenger	FALSE	FALSE	FALSE
TCGA-D8-A1JN	11	61897900	C	G	INCENP	p.Q301E	Missense_Mutation	FALSE	.	0.5	0	18	14	1	0.86113	1	0.556353489	Clonal	passenger	N	N	T	Passenger	FALSE	FALSE	FALSE
TCGA-D8-A1JN	15	45398520	C	T	DUOX2	p.E651K	Missense_Mutation	FALSE	.	0.5	0	56	36	1	0.92648	1	0.760658648	Clonal	passenger	D	D	.	Passenger	FALSE	FALSE	FALSE
TCGA-D8-A1JN	16	31408729	C	T	ITGAD	p.R63W	Missense_Mutation	FALSE	.	0.5	0	20	8	1	0.86857	1	0.578402448	Clonal	passenger	N	D	T	Passenger	FALSE	FALSE	FALSE
TCGA-D8-A1JN	19	7166387	C	T	INSR	p.G547R	Missense_Mutation	FALSE	.	0.5	0	50	35	1	0.92153	1	0.743660685	Clonal	passenger	D	D	T	Passenger	FALSE	FALSE	FALSE
TCGA-D8-A1JN	2	179649010	C	G	TTN	p.K854N	Missense_Mutation	FALSE	.	0.5	0	48	30	1	0.91966	1	0.737437685	Clonal	passenger	N	D	T	Passenger	FALSE	FALSE	FALSE
TCGA-D8-A1JN	9	34996744	G	A	DNAJB5	p.D346N	Missense_Mutation	FALSE	.	0.5	0	34	18	1	0.90198	1	0.680399032	Clonal	likely_pathogenic	D	D	D	Passenger	FALSE	FALSE	FALSE
TCGA-D8-A1JN	23	49106129	C	G	CCDC22	p.I571M	Missense_Mutation	FALSE	.	0.5	0	30	19	1	0.89475	1	0.657724595	Clonal	passenger	D	N	.	Passenger	FALSE	FALSE	FALSE
TCGA-D8-A1XC	7	21516732	C	A	SP4	p.Q572K	Missense_Mutation	FALSE	.	0.5	0	24	25	1	0.80798	1	0.622312652	Clonal	passenger	D	N	T	Passenger	FALSE	FALSE	FALSE
TCGA-D8-A1XY	9	138378571	C	T	PPP1R26	p.R739W	Missense_Mutation	FALSE	.	0.5	0	8	9	1	0.85922	1	0.440933408	Clonal	passenger	N	D	T	Passenger	FALSE	FALSE	FALSE
TCGA-D8-A1XZ	12	13768551	C	A	GRIN2B	p.G459V	Missense_Mutation	FALSE	.	0.5	0	108	96	1	0.9532	1	0.903055036	Clonal	likely_pathogenic	D	.	D	Passenger	FALSE	FALSE	FALSE
TCGA-D8-A1XZ	6	151687962	A	T	ZBTB2	p.M80K	Missense_Mutation	FALSE	.	0.5	0	28	21	1	0.93287	1	0.716517197	Clonal	passenger	D	D	T	Passenger	FALSE	FALSE	FALSE

TCGA-E2-A15H	16	31073089	G	A	ZNF668	p.S410L	Missense_Mutation	FALSE	.	0.5	0	12	15	1	0.88398	1	0.549232031	Clonal	passenger	D	D	T	Passenger	FALSE	FALSE	FALSE
TCGA-D8-A1JN	13	26967647	G	A	CDK8	p.D264N	Missense_Mutation	FALSE	.	0.506024096	0	83	69	1	0.94081	1	0.818507096	Clonal	passenger	D	N	T	Passenger	FALSE	FALSE	FALSE
TCGA-D8-A1XY	1	75037178	C	T	C1orf173	p.E1406K	Missense_Mutation	FALSE	loh	0.506024096	0	83	59	1	0.94197	1	0.89195953	Clonal	passenger	D	N	T	Passenger	FALSE	FALSE	FALSE
TCGA-A8-A08J	4	16597474	C	T	LDB2	p.R87H	Missense_Mutation	FALSE	loh	0.510204082	0	98	51	1	0.48356	1	0.914198464	Clonal	passenger	D	D	.	Passenger	FALSE	FALSE	FALSE
TCGA-BH-A1FM	17	12897815	C	T	ELAC2	p.D679N	Missense_Mutation	FALSE	loh	0.5125	0.013422819	80	149	1	0.97409	1	0.881308447	Clonal	passenger	D	N	T	Passenger	FALSE	FALSE	FALSE
TCGA-D8-A1XZ	13	114150035	C	T	TMCO3	p.R47W	Missense_Mutation	FALSE	.	0.517241379	0	29	41	1	0.87432	1	0.69505048	Clonal	passenger	N	N	T	Passenger	FALSE	FALSE	FALSE
TCGA-D8-A1JN	9	130249962	G	A	LRSAM1	p.E423K	Missense_Mutation	FALSE	.	0.518072289	0	83	45	1	0.93883	1	0.829075916	Clonal	passenger	D	N	T	Passenger	FALSE	FALSE	FALSE
TCGA-E2-A155	19	36211898	AC	A	KMT2B	p.K553Nfs*52	Frame_Shift_Del	FALSE	.	0.518072289	0	83	83	1	0.89256	1	0.842596641	Clonal	passenger	.	.	.	.	FALSE	FALSE	FALSE
TCGA-D8-A1JN	19	40540443	G	A	ZNF780B	p.H775Y	Missense_Mutation	FALSE	loh	0.520408163	0	98	62	1	0.93883	1	0.849659334	Clonal	passenger	N	D	T	Passenger	FALSE	FALSE	FALSE
TCGA-D8-A1JN	12	120541722	G	C	RAB35	p.F45L	Missense_Mutation	FALSE	.	0.52173913	0	69	38	1	0.93466	1	0.809531296	Clonal	passenger	D	D	T	Passenger	FALSE	FALSE	FALSE
TCGA-D8-A1JN	5	140719585	G	A	PCDHGA2	p.M349I	Missense_Mutation	FALSE	.	0.522727273	0	44	33	1	0.9206	1	0.745866961	Clonal	passenger	N	N	T	Passenger	FALSE	FALSE	FALSE
TCGA-D8-A1JN	23	85236772	G	A	CHM	p.S53L	Missense_Mutation	FALSE	.	0.523076923	0	65	43	1	0.93312	1	0.802730786	Clonal	passenger	D	D	T	Passenger	FALSE	FALSE	FALSE
TCGA-D8-A1XC	7	40027851	C	T	CDK13	p.A622V	Missense_Mutation	FALSE	.	0.523809524	0	21	9	1	0.80838	1	0.618188814	Clonal	passenger	N	N	T	Passenger	FALSE	FALSE	FALSE
TCGA-BH-A1FM	2	204599579	C	T	CD28	p.R203C	Missense_Mutation	FALSE	loh	0.526315789	0	19	43	1	0.94775	1	0.654912198	Clonal	passenger	N	D	D	Passenger	FALSE	FALSE	FALSE
TCGA-D8-A1JN	15	72552860	G	A	PARP6	p.Q239*	Nonsense_Mutation	FALSE	.	0.526315789	0	19	16	1	0.87595	1	0.593418353	Clonal	passenger	A	.	.	.	FALSE	FALSE	FALSE
TCGA-D8-A1JN	3	178936091	G	A	PIK3CA	p.E545K	Missense_Mutation	TRUE	.	0.526315789	0	57	72	1	0.92944	1	0.787712418	Clonal	likely_pathogenic	D	D	T	Driver	TRUE	TRUE	TRUE
TCGA-D8-A1XZ	19	19407828	C	A	SUGP1	p.D405Y	Missense_Mutation	FALSE	error	0.526315789	0	19	23	1	0.88876	1	0.669903987	Clonal	passenger	D	N	T	Passenger	FALSE	FALSE	FALSE
TCGA-E9-A54Y	23	17746847	C	T	NHS	p.S1413F	Missense_Mutation	FALSE	loh	0.526315789	0	19	43	1	0.9013	1	0.633661741	Clonal	passenger	D	D	T	Passenger	FALSE	FALSE	FALSE
TCGA-D8-A1JN	12	53680434	C	G	ESPL1	p.S1305*	Nonsense_Mutation	FALSE	.	0.529411765	0	34	33	1	0.91023	1	0.709247803	Clonal	passenger	A	.	.	.	FALSE	FALSE	FALSE
TCGA-D8-A1XC	20	62187465	G	A	C20orf195	p.R150Q	Missense_Mutation	FALSE	.	0.533333333	0	15	14	1	0.78092	1	0.553894304	Clonal	passenger	N	N	.	Passenger	FALSE	FALSE	FALSE
TCGA-D8-A1XC	5	7835571	C	T	C5orf49	p.R63H	Missense_Mutation	FALSE	.	0.536585366	0	41	45	1	0.86246	1	0.753636064	Clonal	passenger	N	N	T	Passenger	FALSE	FALSE	FALSE
TCGA-AR-A0TW	17	46878982	C	T	TTLL6	p.G129S	Missense_Mutation	FALSE	loh	0.538461538	0	104	60	1	0.51126	1	0.922561861	Clonal	passenger	D	.	.	Passenger	FALSE	FALSE	FALSE
TCGA-D8-A1XC	3	164755728	T	C	SI	p.I796V	Missense_Mutation	FALSE	.	0.538461538	0	52	63	1	0.86976	1	0.791484875	Clonal	likely_pathogenic	D	N	D	Passenger	FALSE	FALSE	FALSE
TCGA-E9-A1RB	4	89950787	G	A	FAM13A	p.A14V	Missense_Mutation	FALSE	loh	0.53968254	0	63	84	1	0.95718	1	0.871011897	Clonal	passenger	D	N	T	Passenger	FALSE	FALSE	FALSE
TCGA-E9-A54Y	12	53448060	C	A	TENC1	p.F129L	Missense_Mutation	FALSE	.	0.542857143	0	35	41	1	0.54489	1	0.807277081	Clonal	likely_pathogenic	D	N	D	Passenger	FALSE	FALSE	FALSE
TCGA-E2-A15H	17	40180151	G	A	ZNF385C	p.R362W	Missense_Mutation	FALSE	loh	0.545454545	0	22	30	1	0.67613	1	0.695848273	Clonal	passenger	N	D	T	Passenger	FALSE	FALSE	FALSE
TCGA-E9-A54Y	12	108603944	G	A	WSCD2	p.G182S	Missense_Mutation	FALSE	.	0.55	0	40	36	1	0.4805	1	0.830259702	Subclonal	passenger	D	D	T	Passenger	FALSE	FALSE	FALSE
TCGA-BH-A1FM	7	43506115	C	T	HECW1	p.A954V	Missense_Mutation	FALSE	.	0.552845528	0	123	77	1	0.46288	1	0.937612127	Clonal	likely_pathogenic	D	N	D	Passenger	FALSE	FALSE	FALSE
TCGA-BH-A1FM	16	24788434	C	A	TNRC6A	p.P115Q	Missense_Mutation	FALSE	.	0.555555556	0	9	28	1	0.82743	1	0.509610767	Clonal	passenger	P	N	T	Passenger	FALSE	FALSE	FALSE
TCGA-E9-A54Y	22	38528924	C	T	PLA2G6	p.D331N	Missense_Mutation	FALSE	.	0.5625	0	64	147	1	0.94751	1	0.878988233	Clonal	passenger	D	N	T	Passenger	FALSE	FALSE	FALSE
TCGA-E2-A15R	1	160210035	G	A	DCAF8	p.R59C	Missense_Mutation	FALSE	.	0.565625	0	320	227	1	0.97459	1	0.970401616	Clonal	passenger	D	N	T	Passenger	FALSE	FALSE	FALSE
TCGA-E9-A54Y	11	62343518	C	T	TUT1	p.R596Q	Missense_Mutation	FALSE	loh	0.566666667	0	30	33	1	0.78254	1	0.785533281	Clonal	passenger	D	N	T	Passenger	FALSE	FALSE	FALSE
TCGA-D8-A1XC	22	43933284	CCT	C	EFCAB6	p.Q1340Rfs*43	Frame_Shift_Del	FALSE	.	0.571428571	0	28	12	1	0.83862	1	0.715495178	Clonal	passenger	.	.	.	.	FALSE	FALSE	FALSE
TCGA-E9-A1NI	17	11984789	C	CT	MAP2K4	p.Y113Lfs*19	Frame_Shift_Ins	FALSE	loh	0.571428571	0	28	44	1	0.84887	1	0.770487478	Clonal	likely_pathogenic	D	.	.	.	TRUE	TRUE	TRUE
TCGA-A8-A08J	10	8115926	G	GC	GATA3	p.S426Ifs*81	Frame_Shift_Ins	FALSE	.	0.575163399	0	153	70	1	0.43348	1	0.95276454	Clonal	likely_pathogenic	D	.	.	.	TRUE	TRUE	TRUE
TCGA-E2-A15R	1	201980286	A	G	ELF3	p.S8G	Missense_Mutation	FALSE	.	0.576576577	0.006134969	222	163	1	0.97921	1	0.960010035	Clonal	passenger	D	N	T	Passenger	FALSE	TRUE	TRUE
TCGA-E2-A15H	22	45309833	T	C	PHF21B	p.I234V	Missense_Mutation	FALSE	loh	0.577235772	0	123	158	1	0	1	0.932094885	Clonal	passenger	N	N	T	Passenger	FALSE	FALSE	FALSE
TCGA-D8-A1XY	11	121033025	G	C	TECTA	p.A1740P	Missense_Mutation	FALSE	.	0.583333333	0	12	8	1	0.61885	1	0.605338619	Clonal	passenger	D	D	T	Passenger	FALSE	FALSE	FALSE
TCGA-E9-A1RB	17	7577082	C	G	TP53	p.E286Q	Missense_Mutation	TRUE	loh	0.587301587	0	63	67	1	0.89518	1	0.885506033	Clonal	likely_pathogenic	D	D	D	Passenger	TRUE	TRUE	TRUE
TCGA-E2-A15R	17	42330650	G	T	SLC4A1	p.A716D	Missense_Mutation	FALSE	.	0.588235294	0	102	71	1	0.30386	1	0.89631085	Subclonal	passenger	N	D	T	Passenger	FALSE	FALSE	FALSE
TCGA-AR-A0TW	20	60902626	C	T	LAMA5	p.A1633T	Missense_Mutation	FALSE	.	0.6	0	25	19	1	0.9076	1	0.760564743	Clonal	passenger	D	N	T	Passenger	FALSE	FALSE	FALSE
TCGA-BH-A1FM	1	215821936	G	A	USH2A	p.T4839M	Missense_Mutation	FALSE	.	0.602564103	0	78	74	1	0.32865	1	0.918408005	Clonal	passenger	N	N	T	Passenger	FALSE	FALSE	FALSE
TCGA-D8-A1JN	16	30795272	G	A	ZNF629	p.P126L	Missense_Mutation	FALSE	.	0.615384615	0	13	12	1	0.87446	1	0.582921535	Clonal	passenger	D	N	T	Passenger	FALSE	FALSE	FALSE
TCGA-E2-A15R	14	55369216	C	T	GCH1	p.E56K	Missense_Mutation	FALSE	.	0.615384615	0	26	23	1	0.82516	1	0.731364448	Clonal	likely_pathogenic	D	N	D	Passenger	FALSE	FALSE	FALSE
TCGA-D8-A1JN	7	86468257	G	T	GRM3	p.G476V	Missense_Mutation	FALSE	.	0.617021277	0	47	29	1	0.79299	1	0.82406144	Clonal	likely_pathogenic	D	D	D	Passenger	FALSE	FALSE	FALSE
TCGA-E9-A54Y	16	28914701	TCA	T	ATP2A1	p.L975Afs*23	Frame_Shift_Del	FALSE	.	0.623188406	0	69	48	1	0.28769	1	0.913035086	Clonal	passenger	D	.	.	.	FALSE	FALSE	FALSE
TCGA-AR-A0TW	19	7682695	C	T	CAMSAP3	p.Q1195*	Nonsense_Mutation	FALSE	.	0.625	0	8	9	1	0.89312	1	0.507134079	Clonal	passenger	D	.	.	.	FALSE	FALSE	FALSE
TCGA-D8-A1XZ	17	18881643	G	A	FAM83G	p.R446C	Missense_Mutation	FALSE	loh	0.625	0	24	43	1	0.749	1	0.73527606	Clonal	passenger	D	D	T	Passenger	FALSE	FALSE	FALSE
TCGA-E9-A1NI	1	182811768	A	G	DHX9	p.I23V	Missense_Mutation	FALSE	.	0.630769231	0	195	87	1	0.25741	1	0.96762499	Clonal	passenger	D	N	T	Passenger	FALSE	FALSE	FALSE
TCGA-AR-A0TW	17	15965036	T	A	NCOR1	p.R1854*	Nonsense_Mutation	FALSE	loh	0.653846154	0	26	23	1	0.80975	1	0.789892532	Clonal	likely_pathogenic	A	.	.	.	TRUE	TRUE	TRUE
TCGA-E9-A1RB	13	98828595	C	A	RNF113B	p.G299V	Missense_Mutation	FALSE	loh	0.656716418	0	67	69	1	0.37287	1	0.907394294	Clonal	passenger	D	D	T	Passenger	FALSE	FALSE	FALSE
TCGA-E2-A155	5	141005299	G	A	HDAC3	p.L338F	Missense_Mutation	FALSE	loh	0.663120567	0.001980198	282	505	0.98	0.94462	1	0.884483852	Clonal	passenger	D	D	T	Passenger	FALSE	FALSE	FALSE
TCGA-D8-A1JN	12	109889615	C	T	KCTD10	p.E243K	Missense_Mutation	FALSE	.	0.666666667	0	12	13	1	0.88285	1	0.599760919	Clonal	passenger	D	D	T	Passenger	FALSE	FALSE	FALSE
TCGA-D8-A1XZ	14	101349520	G	A	RTL1	p.R536C	Missense_Mutation	FALSE	.	0.666666667	0	15	47	1	0.89731	1	0.681520424	Clonal	passenger	N	N	T	Passenger	FALSE	FALSE	FALSE
TCGA-E9-A1RB	1	32745462	C	A	LCK	p.F354L	Missense_Mutation	FALSE	loh	0.67	0	100	50	1	0.28088	1	0.942677252	Clonal	likely_pathogenic	D	D	D	Passenger	TRUE	FALSE	FALSE
TCGA-BH-A1FM	22	50719361	C	T	PLXNB2	p.E1269K	Missense_Mutation	FALSE	loh	0.68	0	25	69	1	0.89186	1	0.789174514	Clonal	passenger	D	N	T	Passenger	FALSE	FALSE	FALSE
TCGA-E2-A15R	13	37678978	G	T	CSNK1A1L	p.P139Q	Missense_Mutation	FALSE	loh	0.68503937	0	254	534	0.94	0.76099	1	0.85056275	Clonal	passenger	D	D	T	Passenger	FALSE	FALSE	FALSE
TCGA-D8-A1JN	16	68855966	G	GC	CDH1	p.I594Hfs*11	Frame_Shift_Ins	FALSE	loh	0.68627451	0	51	49	0.91	0.83777	1	0.719347003	Clonal	likely_pathogenic	D	.	.	.	TRUE	TRUE	TRUE
TCGA-BH-A1FM	10	70507134	C	T	CCAR1	p.Q213*	Nonsense_Mutation	FALSE	loh	0.692307692	0.009615385	52	104	1	0.70536	1	0.889883907	Clonal	likely_pathogenic	A	.	.	.	FALSE	FALSE	FALSE
TCGA-E2-A155	12	22677465	C	T	C2CD5	p.R181Q	Missense_Mutation	FALSE	loh	0.697368421	0	228	363	1	0.97279	1	0.91035921	Clonal	likely_pathogenic	D	N	T	Driver	FALSE	FALSE	FALSE
TCGA-D8-A1JN	17	60447652	G	T	EFCAB3	p.M1?	Translation_Start_Site	FALSE	loh	0.7	0	90	96	0.93	0.86394	1	0.786218542	Clonal	likely_pathogenic	D	T	T	Passenger	FALSE	FALSE	FALSE
TCGA-BH-A1FM	17	43213950	C	T	ACBD4	p.R58W	Missense_Mutation	FALSE	loh	0.705882353	0	17	38	1	0.59218	1	0.733585316	Clonal	passenger	D	D	T	Passenger	FALSE	FALSE	FALSE
TCGA-E2-A15R	7	156742517	G	A	NOM1	p.G29E	Missense_Mutation	FALSE	.	0.705882353	0	17	10	1	0.91353	1	0.723208772	Clonal	passenger	N	N	T	Passenger	FALSE	FALSE	FALSE
TCGA-D8-A1JN	22	50301365	TCA	T	ALG12	p.X331_splice	Splice_Site	FALSE	loh	0.708333333	0	24	13	0.94	0.8727	1	0.651141904	Clonal	likely_pathogenic	D	.	.	.	FALSE	FALSE	FALSE
TCGA-E2-A155	5	102432359	C	G	GIN1	p.D394H	Missense_Mutation	FALSE	loh	0.708737864	0	309	420	1	0.98063	1	0.933766882	Clonal	passenger	D	N	T	Passenger	FALSE	FALSE	FALSE
TCGA-E2-A15R	8	25292958	A	G	KCTD9	p.L245S	Missense_Mutation	FALSE	loh	0.714285714	0	63	207	0.98	0.89304	1	0.791626556	Clonal	passenger	D	D	T	Passenger	FALSE	FALSE	FALSE
TCGA-E2-A155	23	63488612	C	T	MTMR8	p.M640I	Missense_Mutation	FALSE	loh	0.726666667	0	150	232	1	0.9728	1	0.908508766	Clonal	passenger	N	N	D	Passenger	FALSE	FALSE	FALSE
TCGA-D8-A1XZ	17	7578431	G	A	TP53	p.Q167*	Nonsense_Mutation	FALSE	loh	0.739130435	0	23	50	1	0.14566	1	0.783434053	Subclonal	likely_pathogenic	A	.	.	.	TRUE	TRUE	TRUE
TCGA-E9-A1NI	16	1735531	A	G	HN1L	p.N46D	Missense_Mutation	FALSE	loh	0.762711864	0	59	55	1	0.29304	1	0.912540682	Clonal	passenger	D	D	T	Passenger	FALSE	FALSE	FALSE
TCGA-E2-A155	5	79028598	C	T	CMYA5	p.S1337L	Missense_Mutation	FALSE	loh	0.768292683	0	82	118	1	0.96123	1	0.891064126	Clonal	passenger	N	D	T	Passenger	FALSE	FALSE	FALSE
TCGA-D8-A1XZ	19	1754710	G	A	ONECUT3	p.C350Y	Missense_Mutation	FALSE	loh	0.769230769	0	13	22	1	0.49599	1	0.684119444	Subclonal	likely_pathogenic	D	D	D	Passenger	FALSE	FALSE	FALSE
TCGA-E2-A15R	1	248039412	G	A	TRIM58	p.G361E	Missense_Mutation	FALSE	loh	0.777777778	0	153	273	1	0.97017	1	0.919103262	Clonal	likely_pathogenic	D	D	D	Passenger	FALSE	FALSE	FALSE
TCGA-E2-A15R	1	29189481	G	A	OPRD1	p.A269T	Missense_Mutation	FALSE	loh	0.792682927	0	82	81	1	0.95914	1	0.886418579	Clonal	passenger	D	N	T	Passenger	FALSE	FALSE	FALSE
TCGA-D8-A1JN	2	113089006	C	G	ZC3H6	p.F837L	Missense_Mutation	FALSE	loh	0.8	0	20	30	1	0.93046	1	0.711548134	Clonal	passenger	N	N	T	Passenger	FALSE	FALSE	FALSE
TCGA-E2-A155	17	7579591	C	G	TP53	p.X33_splice	Splice_Site	FALSE	loh	0.813953488	0	43	83	1	0.94241	1	0.854221384	Clonal	likely_pathogenic	D	.	.	.	TRUE	TRUE	TRUE
TCGA-E2-A15R	11	120351050	A	T	ARHGEF12	p.D1383V	Missense_Mutation	FALSE	loh	0.816326531	0	98	201	1	0.95856	1	0.913658229	Clonal	passenger	D	N	T	Passenger	TRUE	FALSE	FALSE
TCGA-E9-A54Y	11	118359386	G	T	KMT2A	p.E1464*	Nonsense_Mutation	FALSE	loh	0.826666667	0	75	104	1	0.49382	1	0.940026372	Clonal	likely_pathogenic	A	.	.	.	TRUE	FALSE	TRUE
TCGA-D8-A1JN	17	29527590	C	T	NF1	p.Q347*	Nonsense_Mutation	FALSE	loh	0.830188679	0	53	68	1	0.9681	1	0.864594654	Clonal	likely_pathogenic	D	.	.	.	TRUE	TRUE	TRUE
TCGA-D8-A1JN	19	41931969	C	T	B3GNT8	p.V239M	Missense_Mutation	FALSE	loh	0.866666667	0	15	9	1	0.96093	1	0.750360259	Clonal	passenger	D	N	T	Passenger	FALSE	FALSE	FALSE
TCGA-D8-A1JN	16	89341577	G	A	ANKRD11	p.A2498V	Missense_Mutation	FALSE	loh	0.894736842	0	19	16	1	0.94797	1	0.774183371	Clonal	passenger	D	N	T	Passenger	FALSE	FALSE	FALSE

Supplementary Table 3. Clinico-pathologic information of the BRCA1 and BRCA2-associated breast cancers from The Cancer Genome Atlas (TCGA) and International Cancer Genome Consortium (ICGC) included in this study for comparisons.

Sample ID	Study	Age (years)	Estrogen receptor status	HER2 status	Stage	Histologic Subtype	Gene	Germline mutation	Mutation Type
TCGA-AN-A0AL	TCGA	41	Negative	Negative	IIIB	Invasive Ductal Carcinoma	*BRCA1*	c.5125_5126insC;p.Gln1709_Asp1710fs	Frameshift Insertion
TCGA-D8-A1XK	TCGA	55	Negative	Negative	IIB	Invasive Ductal Carcinoma	*BRCA1*	c.3390_3391insT;p.Phe1130_Ser1131fs	Frameshift Insertion
TCGA-E2-A14N	TCGA	37	Negative	Negative	IIB	Invasive Ductal Carcinoma	*BRCA1*	c.329_330insA;p.Lys110_Glu111fs	Frameshift Insertion
TCGA-E9-A22E	TCGA	56	Positive	Positive	IIIA	Invasive Ductal Carcinoma	*BRCA1*	c.5125_5126insC;p.Gln1709_Asp1710fs	Frameshift Insertion
TCGA-E9-A22G	TCGA	47	Negative	Positive	IIA	Invasive Ductal Carcinoma	*BRCA1*	c.5125_5126insC;p.Gln1709_Asp1710fs	Frameshift Insertion
TCGA-E9-A3QA	TCGA	33	-	-	IIA	Medullary Carcinoma	*BRCA1*	c.5125_5126insC;p.Gln1709_Asp1710fs	Frameshift Insertion
TCGA-A2-A0D2	TCGA	45	Negative	Negative	IIA	Invasive Ductal Carcinoma	*BRCA1*	c.4996delG;p.Val1666fs	Frameshift Deletion
TCGA-AN-A0FL	TCGA	62	Negative	Positive	IIA	Invasive Ductal Carcinoma	*BRCA1*	c.3894delA;p.Glu1298fs	Frameshift Deletion
TCGA-AO-A124	TCGA	38	Negative	Negative	IIA	-	*BRCA1*	c.68_69delAG;p.Glu23fs	Frameshift Deletion
TCGA-AR-A24Q	TCGA	49	Positive	Negative	IIB	Invasive Ductal Carcinoma	*BRCA1*	c.782delG;p.Ser261fs	Frameshift Deletion
TCGA-E9-A244	TCGA	54	-	-	IIA	Invasive Ductal Carcinoma	*BRCA1*	c.3894delA;p.Glu1298fs	Frameshift Deletion
TCGA-EW-A1P4	TCGA	43	Negative	Negative	IIA	Medullary Carcinoma	*BRCA1*	c.1740_1743delCAGT;p.Val580fs	Frameshift Deletion
TCGA-BH-A0BL	TCGA	35	Negative	Negative	I	Invasive Ductal Carcinoma	*BRCA1*	c.181T>G;p.Cys61Gly	Missense
TCGA-C8-A12K	TCGA	80	-	-	IIB	Invasive Ductal Carcinoma	*BRCA1*	c.5095C>T;p.Arg1699Trp	Missense
TCGA-D8-A147	TCGA	45	Negative	Negative	-	Invasive Ductal Carcinoma	*BRCA1*	c.181T>G;p.Cys61Gly	Missense
TCGA-A7-A4SE	TCGA	54	Negative	Negative	IIA	Invasive Ductal Carcinoma	*BRCA1*	c.913G>T;p.Glu305*	Nonsense
TCGA-AN-A0FX	TCGA	52	Negative	Positive	IIA	Invasive Ductal Carcinoma	*BRCA1*	c.3826C>T;p.Gln1276*	Nonsense
TCGA-E2-A14Z	TCGA	64	Positive	Negative	I	Invasive Ductal Carcinoma	*BRCA1*	c.453A>C	Splice site
PD24202	ICGC (Nik-Zainal et al.)	40	Negative	Negative	III	Invasive Ductal Carcinoma	*BRCA1*	c.5497_5507delGTGACCCGAGAinsA; p.V1833fs*7	Frameshift Insertion
PD24186	ICGC (Nik-Zainal et al.)	34	Negative	Negative	III	Invasive Ductal Carcinoma	*BRCA1*	c.4338_4339insAGAA; p.Q1447fs*16	Frameshift Insertion
PD4107	ICGC (Nik-Zainal et al.)	33	Negative	Negative	III	Invasive Ductal Carcinoma	*BRCA1*	c.1016_1017insA; p.V340fs*6	Frameshift Insertion
PD23562	ICGC (Nik-Zainal et al.)	47	Negative	Negative	III	Invasive Ductal Carcinoma	*BRCA1*	c.922_924delAGCinsT; p.S308fs*1	Frameshift Insertion
PD13296	ICGC (Nik-Zainal et al.)	61	Negative	Negative	III	Invasive Ductal Carcinoma	*BRCA1*	c.5333-36_5406+400del510; p.?	Frameshift Deletion
PD9004	ICGC (Nik-Zainal et al.)	36	Negative	Negative	III	Invasive Ductal Carcinoma	*BRCA1*	c.4391delC; p.P1464fs*2	Frameshift Deletion
PD3905	ICGC (Nik-Zainal et al.)	34	Negative	Negative	III	Invasive Ductal Carcinoma	*BRCA1*	c.4186-1642_4357+2021del3835; p.?	Frameshift Deletion
PD4005	ICGC (Nik-Zainal et al.)	39	Negative	Negative	III	Invasive Ductal Carcinoma	*BRCA1*	c.3710delT; p.I1237fs*27	Frameshift Deletion
PD11327	ICGC (Nik-Zainal et al.)	59	Negative	Negative	III	Invasive Ductal Carcinoma	*BRCA1*	c.3084_3094delTAATAACATTA; p.N1029fs*5	Frameshift Deletion
PD10014	ICGC (Nik-Zainal et al.)	64	Negative	Negative	III	Invasive Ductal Carcinoma	*BRCA1*	c.2681_2682delAA; p.K894fs*8	Frameshift Deletion
PD3890	ICGC (Nik-Zainal et al.)	41	Negative	Negative	-	Invasive Ductal Carcinoma	*BRCA1*	c.2501delG; p.G834fs*12	Frameshift Deletion
PD5930	ICGC (Nik-Zainal et al.)	30	Negative	Negative	III	Invasive Ductal Carcinoma	*BRCA1*	c.2475delC; p.D825fs*21	Frameshift Deletion
PD8980	ICGC (Nik-Zainal et al.)	79	Positive	Negative	III	Invasive Ductal Carcinoma	*BRCA1*	c.2475delC; p.D825fs*21	Frameshift Deletion
PD13299	ICGC (Nik-Zainal et al.)	41	Negative	Negative	III	Invasive Ductal Carcinoma	*BRCA1*	c.1556delA; p.K519fs*13	Frameshift Deletion
PD5945	ICGC (Nik-Zainal et al.)	33	Negative	Negative	III	Invasive Ductal Carcinoma	*BRCA1*	c.697_698delGT; p.V233fs*4	Frameshift Deletion
PD6413	ICGC (Nik-Zainal et al.)	28	Negative	Negative	III	Invasive Ductal Carcinoma	*BRCA1*	c.386delG; p.G129fs*34	Frameshift Deletion
PD6731	ICGC (Nik-Zainal et al.)	43	Negative	Negative	-	Invasive Ductal Carcinoma	*BRCA1*	c.68_69delAG; p.E23fs*17	Frameshift Deletion
PD5948	ICGC (Nik-Zainal et al.)	47	Negative	Negative	III	Invasive Ductal Carcinoma	*BRCA1*	c.442-1901_4358-1399del23768; p.?	Frameshift Deletion
PD9702	ICGC (Nik-Zainal et al.)	50	Negative	Negative	-	Invasive Ductal Carcinoma	*BRCA1*	c.5095C>T; p.R1699W	Missense
PD6406	ICGC (Nik-Zainal et al.)	34	Negative	Negative	III	Invasive Ductal Carcinoma	*BRCA1*	c.5074G>A; p.D1692N	Missense
PD8621	ICGC (Nik-Zainal et al.)	60	Negative	Negative	III	Invasive Ductal Carcinoma	*BRCA1*	c.5503C>T; p.R1835*	Nonsense
PD24337	ICGC (Nik-Zainal et al.)	32	Negative	Negative	III	Invasive Ductal Carcinoma	*BRCA1*	c.5260G>T; p.E1754*	Nonsense
PD13771	ICGC (Nik-Zainal et al.)	80	Positive	Negative	II	Invasive Ductal Carcinoma	*BRCA1*	c.3549_3550delAGinsT; p.K1183fs*26	Nonsense
PD13297	ICGC (Nik-Zainal et al.)	35	Negative	Negative	III	Invasive Ductal Carcinoma	*BRCA1*	c.3178G>T; p.E1060*	Nonsense
PD5935	ICGC (Nik-Zainal et al.)	41	Negative	Negative	III	Invasive Ductal Carcinoma	*BRCA1*	c.3178G>T; p.E1060*	Nonsense
PD14442	ICGC (Nik-Zainal et al.)	80	Positive	Negative	II	Invasive Lobular Carcinoma	*BRCA1*	c.1687C>T; p.Q563*	Nonsense
PD4006	ICGC (Nik-Zainal et al.)	39	Negative	Negative	III	Invasive Ductal Carcinoma	*BRCA1*	c.1687C>T; p.Q563*	Nonsense
PD22355	ICGC (Nik-Zainal et al.)	44	Negative	Negative	III	Invasive Ductal Carcinoma	*BRCA1*	c.390C>A; p.Y130*	Nonsense
PD23574	ICGC (Nik-Zainal et al.)	44	Negative	Negative	III	Invasive Ductal Carcinoma	*BRCA1*	c.390C>A; p.Y130*	Nonsense
PD23578	ICGC (Nik-Zainal et al.)	55	Negative	Negative	III	Invasive Ductal Carcinoma	*BRCA1*	c.390C>A; p.Y130*	Nonsense
PD13627	ICGC (Nik-Zainal et al.)	28	Negative	Negative	III	Invasive Ductal Carcinoma	*BRCA1*	c.4185-1643_4357+2032del3647; p?	Splice site
TCGA-A2-A0T0	TCGA	59	Negative	Negative	IIB	Invasive Ductal Carcinoma	*BRCA2*	c.6942_6945delAATA;p.Thr2314fs	Frameshift Deletion
TCGA-A8-A097	TCGA	65	Positive	Positive	IIB	Invasive Ductal Carcinoma	*BRCA2*	c.657_658delTG;p.Thr219fs	Frameshift Deletion
TCGA-AO-A03V	TCGA	41	Positive	Negative	I	Invasive Ductal Carcinoma	*BRCA2*	c.5946delT;p.Ser1982fs	Frameshift Deletion
TCGA-AR-A2LN	TCGA	65	Positive	Negative	IIA	Invasive Lobular Carcinoma	*BRCA2*	c.8533_8534delAG;p.Arg2845fs	Frameshift Deletion
TCGA-B6-A0I8	TCGA	46	-	-	X	Invasive Ductal Carcinoma	*BRCA2*	c.7683_7684delGT;p.Gln2561fs	Frameshift Deletion
TCGA-BH-A1FN	TCGA	34	Positive	NA	IIA	Invasive Ductal Carcinoma	*BRCA2*	c.2806_2809delAAAC;p.Lys936fs	Frameshift Deletion
TCGA-C8-A8HR	TCGA	49	-	-	IIIA	Invasive Lobular Carcinoma	*BRCA2*	c.8996_8997delTG;p.Leu2999fs	Frameshift Deletion
TCGA-D8-A1JD	TCGA	41	Positive	Negative	IIB	Invasive Ductal Carcinoma	*BRCA2*	c.6847delC;p.Pro2283fs	Frameshift Deletion
TCGA-E2-A15S	TCGA	34	Positive	Negative	IIB	Invasive Ductal Carcinoma	*BRCA2*	c.1262delA;p.Gln421fs	Frameshift Deletion
TCGA-EW-A1PD	TCGA	61	Positive	Positive	IIA	Invasive Ductal Carcinoma	*BRCA2*	c.767_771delCAAAT;p.Thr256fs	Frameshift Deletion
TCGA-HN-A2NL	TCGA	56	Negative	Negative	IIA	Invasive Lobular Carcinoma	*BRCA2*	c.6642delT;p.Thr2214fs	Frameshift Deletion
TCGA-AR-A24L	TCGA	26	Positive	Negative	IIB	Invasive Ductal Carcinoma	*BRCA2*	c.1806_1807insA;p.Gly602_Lys603fs	Frameshift Insertion
TCGA-BH-A0B4	TCGA	65	Positive	Positive	IIIA	Invasive Ductal Carcinoma	*BRCA2*	c.8203_8204insC;p.Pro2735_Leu2736fs	Frameshift Insertion
TCGA-BH-A0BZ	TCGA	59	Positive	Negative	IIIA	Invasive Ductal Carcinoma	*BRCA2*	c.9090_9091insA;p.Thr3030_Lys3031fs	Frameshift Insertion
TCGA-BH-A8FZ	TCGA	58	Positive	-	IA	Invasive Lobular Carcinoma	*BRCA2*	c.1806_1807insA;p.Gly602_Lys603fs	Frameshift Insertion
TCGA-E9-A1NE	TCGA	28	Positive	Negative	IIB	Invasive Ductal Carcinoma	*BRCA2*	c.3975_3976insTGCT;p.Thr1325_Ala1326fs	Frameshift Insertion
TCGA-A2-A25E	TCGA	34	Positive	Equivocal	IIIA	Invasive Ductal Carcinoma	*BRCA2*	c.9294C>G;p.Tyr3098*	Nonsense
TCGA-A8-A09A	TCGA	40	Positive	Negative	X	Invasive Ductal Carcinoma	*BRCA2*	c.5857G>T;p.Glu1953*	Nonsense
TCGA-A8-A09W	TCGA	70	Positive	Negative	IIIB	Invasive Lobular Carcinoma	*BRCA2*	c.4552G>T;p.Glu1518*	Nonsense
TCGA-BH-A0AZ	TCGA	47	Positive	Negative	IIIA	Invasive Ductal Carcinoma	*BRCA2*	c.5864C>A;p.Ser1955*	Nonsense
TCGA-LL-A740	TCGA	61	Negative	Negative	IA	Invasive Ductal Carcinoma	*BRCA2*	c.517A>G	Splice site
TCGA-D8-A1Y0	TCGA	65	Positive	Negative	IIIA	Invasive Ductal Carcinoma	*BRCA2*	c.314_317delTAGG	Splice site
TCGA-A2-A0SU	TCGA	66	Positive	Negative	IIA	Invasive Ductal Carcinoma	*BRCA2*	Y3308*, K3326*	Nonsense
PD22358	ICGC (Nik-Zainal et al.)	38	Negative	Negative	III	Invasive Ductal Carcinoma	*BRCA2*	c.8295_8296insAC; p.P2767fs*11	Frameshift Insertion
PD8984	ICGC (Nik-Zainal et al.)	62	Negative	Negative	III	Invasive Ductal Carcinoma	*BRCA2*	c.9089_9090insA; p.T3033fs*11	Frameshift Insertion
PD4874	ICGC (Nik-Zainal et al.)	64	Negative	Negative	III	Invasive Ductal Carcinoma	*BRCA2*	c.767_771delCAAAT; p.N257fs*17	Frameshift Deletion
PD4875	ICGC (Nik-Zainal et al.)	45	Negative	Negative	III	Invasive Ductal Carcinoma	*BRCA2*	c.767_771delCAAAT; p.N257fs*17	Frameshift Deletion
PD4955	ICGC (Nik-Zainal et al.)	49	Negative	Negative	II	Invasive Ductal Carcinoma	*BRCA2*	c.767_771delCAAAT; p.N257fs*17	Frameshift Deletion
PD4956	ICGC (Nik-Zainal et al.)	55	Negative	Negative	III	Invasive Ductal Carcinoma	*BRCA2*	c.767_771delCAAAT; p.N257fs*17	Frameshift Deletion
PD6416	ICGC (Nik-Zainal et al.)	42	Negative	Negative	III	Invasive Ductal Carcinoma	*BRCA2*	c.767_771delCAAAT; p.N257fs*17	Frameshift Deletion
PD4872	ICGC (Nik-Zainal et al.)	40	Positive	Negative	-	Invasive Ductal Carcinoma	*BRCA2*	c.767_771delCAAAT; p.N257fs*17	Frameshift Deletion
PD4876	ICGC (Nik-Zainal et al.)	30	Positive	Negative	-	Invasive Ductal Carcinoma	*BRCA2*	c.767_771delCAAAT; p.N257fs*17	Frameshift Deletion
PD4951	ICGC (Nik-Zainal et al.)	39	Positive	Negative	II	Invasive Ductal Carcinoma	*BRCA2*	c.767_771delCAAAT; p.N257fs*17	Frameshift Deletion
PD4952	ICGC (Nik-Zainal et al.)	44	Positive	Negative	III	Invasive Ductal Carcinoma	*BRCA2*	c.767_771delCAAAT; p.N257fs*17	Frameshift Deletion
PD4953	ICGC (Nik-Zainal et al.)	67	Positive	Negative	II	Invasive Ductal Carcinoma	*BRCA2*	c.767_771delCAAAT; p.N257fs*17	Frameshift Deletion
PD4954	ICGC (Nik-Zainal et al.)	51	Positive	Negative	II	Invasive Ductal Carcinoma	*BRCA2*	c.767_771delCAAAT; p.N257fs*17	Frameshift Deletion
PD4957	ICGC (Nik-Zainal et al.)	38	Positive	Negative	II	Invasive Ductal Carcinoma	*BRCA2*	c.767_771delCAAAT; p.N257fs*17	Frameshift Deletion
PD4958	ICGC (Nik-Zainal et al.)	48	Positive	Negative	II	Invasive Ductal Carcinoma	*BRCA2*	c.767_771delCAAAT; p.N257fs*17	Frameshift Deletion
PD8610	ICGC (Nik-Zainal et al.)	38	Positive	Negative	III	Invasive Ductal Carcinoma	*BRCA2*	c.1889delC; p.T630fs*14	Frameshift Deletion
PD24191	ICGC (Nik-Zainal et al.)	29	Negative	Negative	III	Invasive Ductal Carcinoma	*BRCA2*	c.5574_5577delAATT; p.I1859fs*3	Frameshift Deletion
PD4959	ICGC (Nik-Zainal et al.)	73	Positive	Negative	III	Invasive Ductal Carcinoma	*BRCA2*	c.6482_6485delACAA; p.K2162fs*5	Frameshift Deletion
PD3904	ICGC (Nik-Zainal et al.)	39	Positive	Negative	II	Invasive Ductal Carcinoma	*BRCA2*	c.6482_6485delACAA; p.K2162fs*5	Frameshift Deletion
PD8978	ICGC (Nik-Zainal et al.)	61	Positive	Negative	III	Invasive Ductal Carcinoma	*BRCA2*	c.8276_8288delTGGGCTCTCCTGA; p.G2760fs*13	Frameshift Deletion
PD24333	ICGC (Nik-Zainal et al.)	80	Negative	Negative	III	Invasive Ductal Carcinoma	*BRCA2*	c.9274_9275delTA; p.Y3092fs*18	Frameshift Deletion
PD8969	ICGC (Nik-Zainal et al.)	48	Negative	Negative	III	Invasive Ductal Carcinoma	*BRCA2*	c.5609T>A; p.F1870Y	Missense
PD8832	ICGC (Nik-Zainal et al.)	53	Negative	Negative	III	Invasive Ductal Carcinoma	*BRCA2*	c.3362C>G; p.S1121*	Nonsense
PD4116	ICGC (Nik-Zainal et al.)	32	Positive	Negative	III	Invasive Ductal Carcinoma	*BRCA2*	c.3455T>G; p.L1152*	Nonsense
PD7217	ICGC (Nik-Zainal et al.)	61	Negative	Negative	III	Invasive Ductal Carcinoma	*BRCA2*	c.5286T>A; p.Y1762*	Nonsense
PD3945	ICGC (Nik-Zainal et al.)	59	Positive	Negative	III	Invasive Ductal Carcinoma	*BRCA2*	c.6065C>G; p.S2022*	Nonsense
PD4604	ICGC (Nik-Zainal et al.)	49	Positive	Negative	III	Invasive Lobular Carcinoma	*BRCA2*	c.8067T>A; p.C2689*	Nonsense
PD23558	ICGC (Nik-Zainal et al.)	56	Positive	Negative	III	Invasive Ductal Carcinoma	*BRCA2*	c.8314G>T; p.E2772*	Nonsense
PD4115	ICGC (Nik-Zainal et al.)	54	Positive	Negative	III	Invasive Ductal Carcinoma	*BRCA2*	c.9294C>A; p.Y3098*	Nonsense
PD13418	ICGC (Nik-Zainal et al.)	46	Negative	Negative	III	Invasive Ductal Carcinoma	*BRCA2*	c.7007+2T>C; p.?	Splice site
PD18259	ICGC (Nik-Zainal et al.)	48	Positive	Negative	III	Invasive Lobular Carcinoma	*BRCA2*	c.7618-1G>A; p.?	Splice site
